# A Zincke-Inspired Cycloreversion/Cyclization Sequence with Arrested Rearomatization: Synthesis of 2‑Aminodihydropyridinium Complexes

**DOI:** 10.1021/acs.organomet.5c00199

**Published:** 2025-08-15

**Authors:** Jonathan D. Dabbs, Caleb C. Taylor, Benjamin F. Livaudais, Alvin Q. Meng, Brian T. Quillin, Diane A. Dickie, W. Dean Harman

**Affiliations:** Department of Chemistry, 2358 University of Virginia, Charlottesville, Virginia 22904, United States

## Abstract

The Zincke reaction combines a pyridinium salt bearing an *N*-withdrawing group and a primary aliphatic amine to form an alkylated pyridinium salt through a ring-opening/ring-closing sequence. Herein, we explore the analogous reaction sequence for a pyridinium salt η^2^-bound to a transition metal. We find that the *N*-sulfonylated pyridinium ligand (pyR^1^, where R^1^ = mesyl or tosyl) of [WTp­(NO)­(PMe_3_)­(η^2^-pyR^1^)]­OTf selectively reacts with a primary amine, and the resulting 2-aminodihydropyridine complex then undergoes a tungsten-stabilized ring-scission to form the corresponding η^2^-azatriene complex. Subsequent ring-closure between the newly installed amine and the sulfonylated imine results in a new aminodihydropyridinium species. This dihydropyridinium resists rearomatization due to a stabilizing influence of the tungsten fragment. Subsequent displacement of the sulfonamide by pendent heteroatoms leads to the formation of new heterocyclic frameworks. Herein the syntheses of 30 heterocyclic complexes are described (3 characterized by SC-XRD) including 7 examples of multicyclic systems.

## Graphical abstract

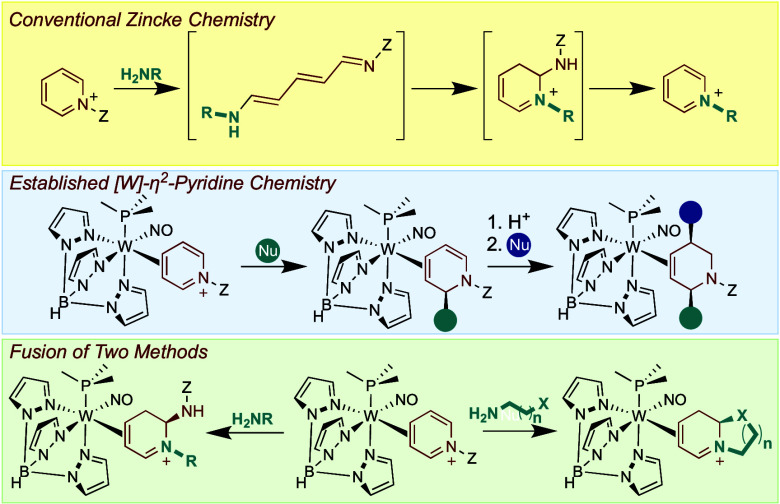

## Introduction

For over a century, the Zincke reaction has been an important tool in *N*-heterocycle synthesis.^1,2^ An *N*-dinitrobenzene (DNB)-substituted pyridinium salt and an amine are utilized to form a 2-aminodihydropyridine, which then can undergo a spontaneous ring-scission (Figure 1A). While secondary amines generate aminoazatrienes that readily convert into Zincke aldehydes,^3^ the product of the analogous reaction with primary amines can undergo a subsequent ring-closure, expelling the amino-DNB group to form a new pyridinium salt. This reaction sequence has been optimized and successfully applied to the syntheses of various alkaloids including strychnine, porothramycin, and norfluorocurarine.^4–10^

**Figure 1:**
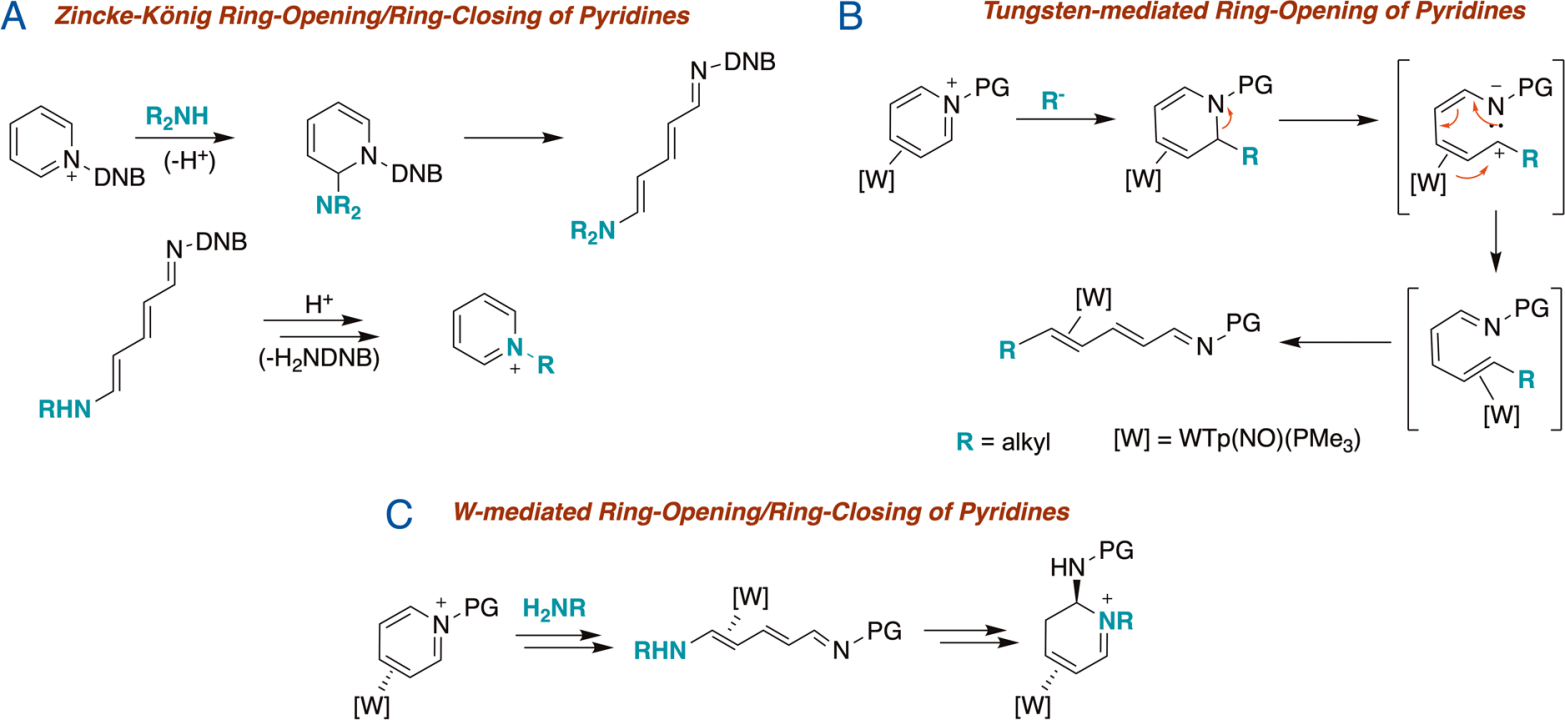
(A) Reaction reported by Zincke with *N*-DNB pyridinium salts and amines. (B) Previously reported reactivity of η^2^-bound alkylated DHPs. (C) Proposed Zincke-like formation of new N-heterocyclic complexes of tungsten.

In a previous report, we described the preparation of a series of dihydropyridine complexes (DHPs) η^2^-bound to {WTp­(NO)­(PMe_3_)} ([W]; Tp = hydridotris­(pyrazolyl)­borate; Figure 1B).^11^ These compounds were prepared from the reaction of an η^2^-*N*-tosylpyridinium precursor (**4D**) and various nucleophilic alkylating agents. We hypothesized that the replacement of the alkyl nucleophile by a primary amine might initiate a Zincke-like ring-opening/ring-closing process, thereby providing access to new types of heterocyclic organometallic complexes. This strategy would circumvent direct ligand substitution reactions, which often are incompatible with desired functional groups.^12^

## Results and Discussion

The decagram-scale synthesis of the sulfonylated pyridinium complex **4D** from an η^2^-anisole complex (**1**) was previously described via η^2^-pyridinium intermediates (**2**, **3**; Figure 2A).^13^ Subsequent *N*-mesylation generates **4D**, which after trituration is isolated with a coordination diastereomer ratio (cdr) greater than 20:1, favoring the distal isomer.^13^ When **4D** was allowed to react with a solution of methylamine at −40 °C over a period of 5 min, the η^2^-5,6-dihydro*-*1-methyl-pyridinium complex **6a** was obtained in good yield (73%). The structural identity of **6a** was confirmed by 2D NMR and high-resolution mass spectrometry (HRMS) analyses (Figure 2B,C). NMR data indicate the presence of two methyl groups, one on the heterocyclic nitrogen and the other now incorporated into the sulfonamide functional group at C6. The stereochemical assignment of C6 was supported by a strong nuclear Overhauser effect (NOE) interaction between the methine proton of C6 and the C5 proton *syn* to the [W] fragment. The latter was in turn assigned through its strong NOE interaction with the PMe_3_ ligand.

**Figure 2:**
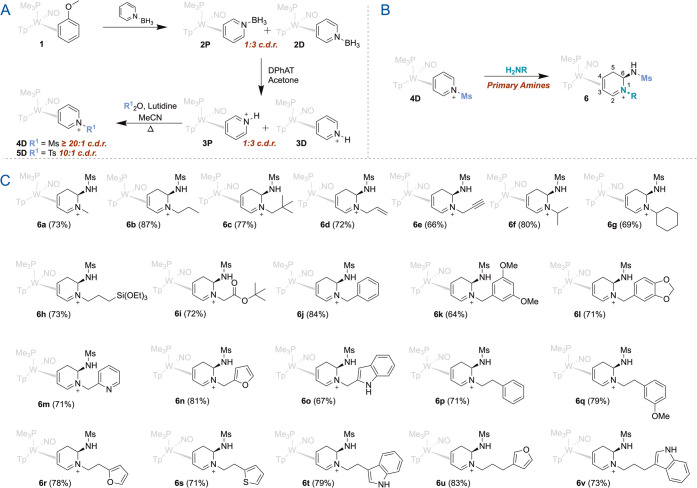
(A) The preparation of **4D** and **5D** from anisole complex 1 and pyridine borane. (B) The reaction of primary amines with mesylpyridinium complex **4D**. (C) Scope of dihydroaminopyridinium complexes accessible from **4D**. All cations depicted represent triflate salts.

An extensive array of analogous dihydropyridinium complexes (**6a**-**6v**) was generated via this approach by adding various aliphatic primary amines to **4D** under similar reaction conditions. The range of groups tethered to the dihydropyridinium nitrogen includes various alkyl, allyl, propargyl, silyl, and aromatic fragments (Figure 2B,C). These complexes were all obtained as single constitutional isomers and diastereomers (constitutional isomer ratio (ir) > 20:1; diastereomer ratio (dr) > 20:1) in yields ranging from 64 to 87%. The tertiary adamantyl- and t-butylamine failed to react, as did aryl amines and amides. Ammonia and zwitterionic amino acids reacted but failed to afford clean products. Similar reactions were observed when the tosylated derivative **5** was substituted for **4**, but in many cases, the recovered products were a mixture of the desired dihydropyridinium complex and the corresponding rearomatized material.

### Intramolecular Variations

The ability to isolate compounds **6a**-**6v** prior to them rearomatizing provided an opportunity: We posited that tethered nucleophiles might be particularly effective at displacing the sulfonamidyl group, thereby providing a route to secondary ring-closures. Although the desired η^2^-dihydroimidazopyridinium complex **7a** was generated after subjecting **4D** to ethylenediamine (Figure 3), subsequent experiments determined that **5D**, the *N*-tosylated analog of **4D**, was a more suitable precursor for generating these polycyclic compounds. The increased electron-withdrawing ability of the tosyl group over the mesyl group renders the sulfonamide a better leaving group, and while maintaining the Ms group was desired in **6a**–**6v**, a better leaving group would seem beneficial for the formation of bicyclics (i.e., **7a**-**7g**; *vide infra*). While reaction conditions varied, the general method utilized a 1:1 MeCN/DCM solution of **5D** stirred with ∼ 3 equiv of the bifunctional amine (H_2_N–X in Figure 3). Subsequent addition of diethyl ether induced a precipitation of the desired product. The polycyclic complexes **7a**-**7g** that were successfully produced via this method included 5, 6, and 7-membered ring closures with carbon, nitrogen, and sulfur pendent nucleophiles. The ring-closures occur stereoselectively, with the bridgehead hydrogen oriented toward the metal. This configuration is supported by NOE and single crystal X-ray diffraction (SC-XRD) data (**7a** and **7b**; Figure 4). Formation of the exocyclic ring through an electrophilic aromatic substitution (EAS) reaction is also possible, as **7g** was produced via an EAS reaction with a pendent indole moiety. This reaction is particularly noteworthy owing to the pharmacological relevance of **7g**, which shares structural similarity to the α_1_-adrenergic receptor antagonist yohimbine, its diastereomers rauhimbine and rauwolscine, and its derivatives spegatrine, ajmalicine, and reserpine.^14,15^

**Figure 3:**
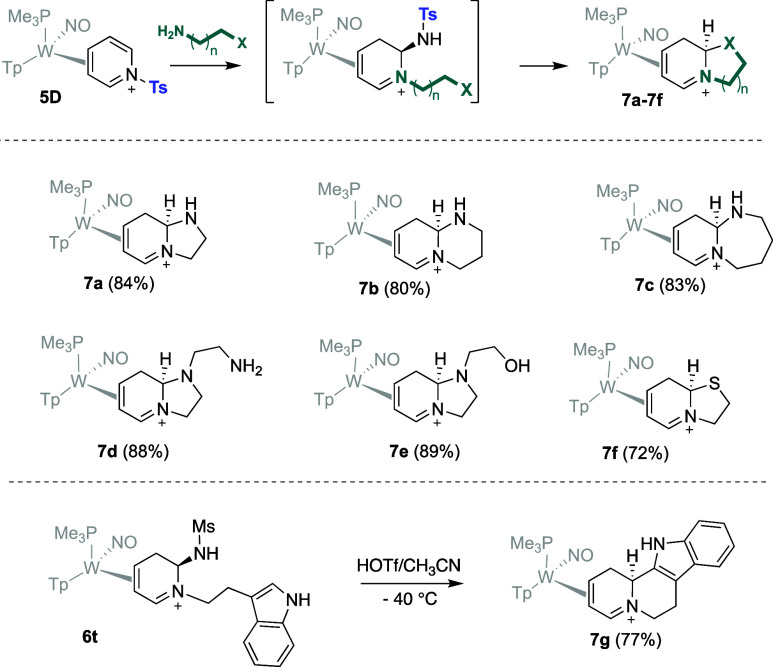
Polycyclic-fused dihydropyridinium [W] complexes **7a**–**7f** generated from a single-pot reaction of **5D** with a primary aliphatic amine tethered to a second nucleophile (X), and the ring-closure of the tryptamine derivative **6t**.

**Figure 4:**
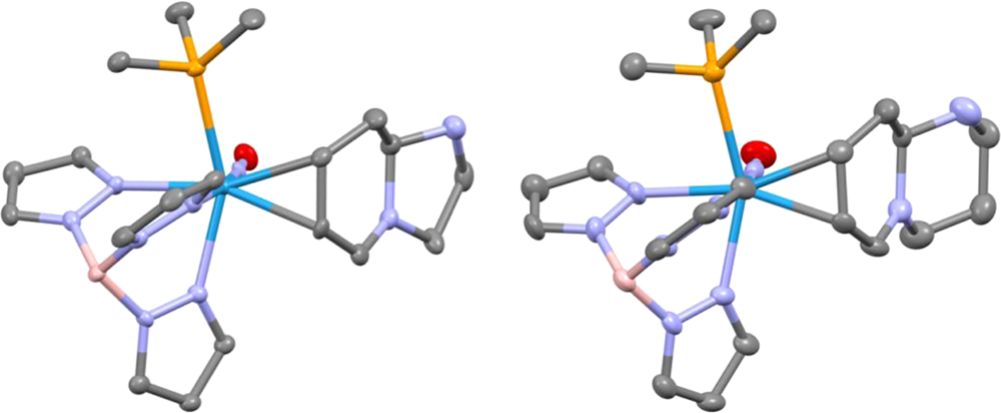
ORTEP/ellipsoid diagrams of **7a** (left) and **7b** (right). Hydrogens and triflate counteranions omitted.

### Control Reactions

Compound **4D** was combined with the secondary amine morpholine at −30 °C in a 50/50 mixture of dichloromethane (DCM) and propionitrile (EtCN). After reacting for 30 min, the mixture was added to a stirring solution of diethyl ether (Et_2_O), which immediately formed a light tan powder that was collected, washed, and dried. ^1^H NMR data revealed a mixture of 2 isomers with nearly identical features. 2D NMR data indicate that **8** is formed as a mixture of E/Z isomers as shown in Figure 5. Proton–proton coupling data for the major isomer of **8** closely resemble those of an analogous compound derived from an indole.^11^ When **5D** was subjected to a much higher concentration of methylamine than reported for the formation of **6a**, a single product was precipitated in diethyl ether and observed via ^1^H NMR, which was determined via 2D NMR analysis and HRMS data to be a [W]-dihydropyridinium complex bearing 2 equiv of methylamine (**9** in Figure 5).

**Figure 5:**
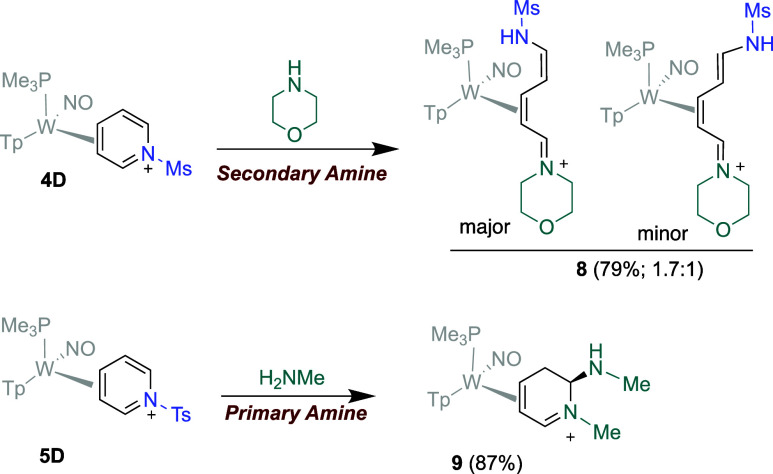
(Top) aminoazatriene [W] complex formed from adding a secondary amine (morpholine) to **4D**. (Bottom) a one-pot Zincke heteroatom substitution and sulfonamide substitution observed when adding methylamine to **5D**.

### Observed Rearomatization

While acquiring 2D NMR spectra on various samples of **6**, we noticed the partial rearomatization of these dihydropyridine complexes. An intentional rearomatization procedure was developed and applied to **6b**. A DCM solution of this compound was treated with 2 equiv of TEA (Figure 6). The desired product (**10**) was then precipitated in stirring diethyl ether. Interestingly, both the NMR samples and the synthesis procedure yielded a ∼1:1 cdr mixture of the rearomatized product (**10D** and **10P**).

**Figure 6:**
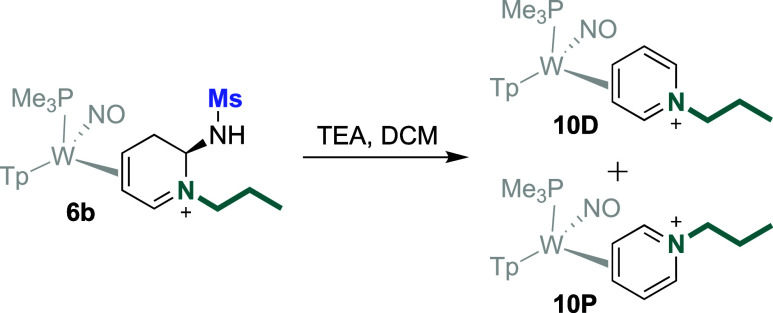
Rearomatization of **6b** occurs in the presence of base at room temperature and produces a mixture of distal (**10D**) and proximal (**10P**) coordination diastereomers.

### Proposed Mechanism

The syntheses of compounds **6**, **7**, **8**, and **9** all begin the same: The addition of an amine to **4D**/**5D** to form an amino-DHP complex (**I**). Aided by the π-backbonding interaction of the tungsten,^16^ **I** then ring-opens to an aminoazatriene **8**. With a secondary amine (e.g., mopholine), the reaction stops at this point (**8**). Alternatively, when a primary amine is used (e.g., methylamine), **8** undergoes an isomerization to form a conjugated sulfonylimine (**II**). A subsequent cyclization occurs with the sulfonamidyl group oriented anti to the tungsten (**III**; dr >20:1). Compounds **6a**-**6v** are then formed, we posit, via the protonation of **III** at C5 to generate the α,β-unsaturated iminium **6**, then 6 is protonated on the sulfonamide to facilitate its removal to form **IV**. This purported dicationic intermediate (**IV**; Figure 7) has been modeled with DFT (SI), and the structure can be interpreted as two conformers (**p** and **d**) of a distorted π-allyl cation complex of W(0), conjugated to an iminium. Using this formalism, the dicationic ligand would be stabilized by the π-donating W(0) fragment, as has been observed for carbocyclic “dicationic ligands” bound to {WTp­(NO)­PMe_3_}.^17–19^ Alternatively, this complex could be regarded as a W­(II)-allyl anion complex (Figure 7). Regardless of the formalism, the ligand is highly electrophilic and can react with a second nucleophile (H_2_NR^4^) to form the final aminodihydropyridinium salt (**7**, **9**). In the absence of a suitable nucleophile, the protonation at C5 reverses, and rearomatization occurs, resulting in a mixture of [W]-*N*-alkylated pyridinium coordination diastereomers (**11D** and **11P**). Once rearomatization occurs, these *N*-alkylpyridinium complexes (i.e., **10** or **11**) show no further reactivity with primary amines.

**Figure 7:**
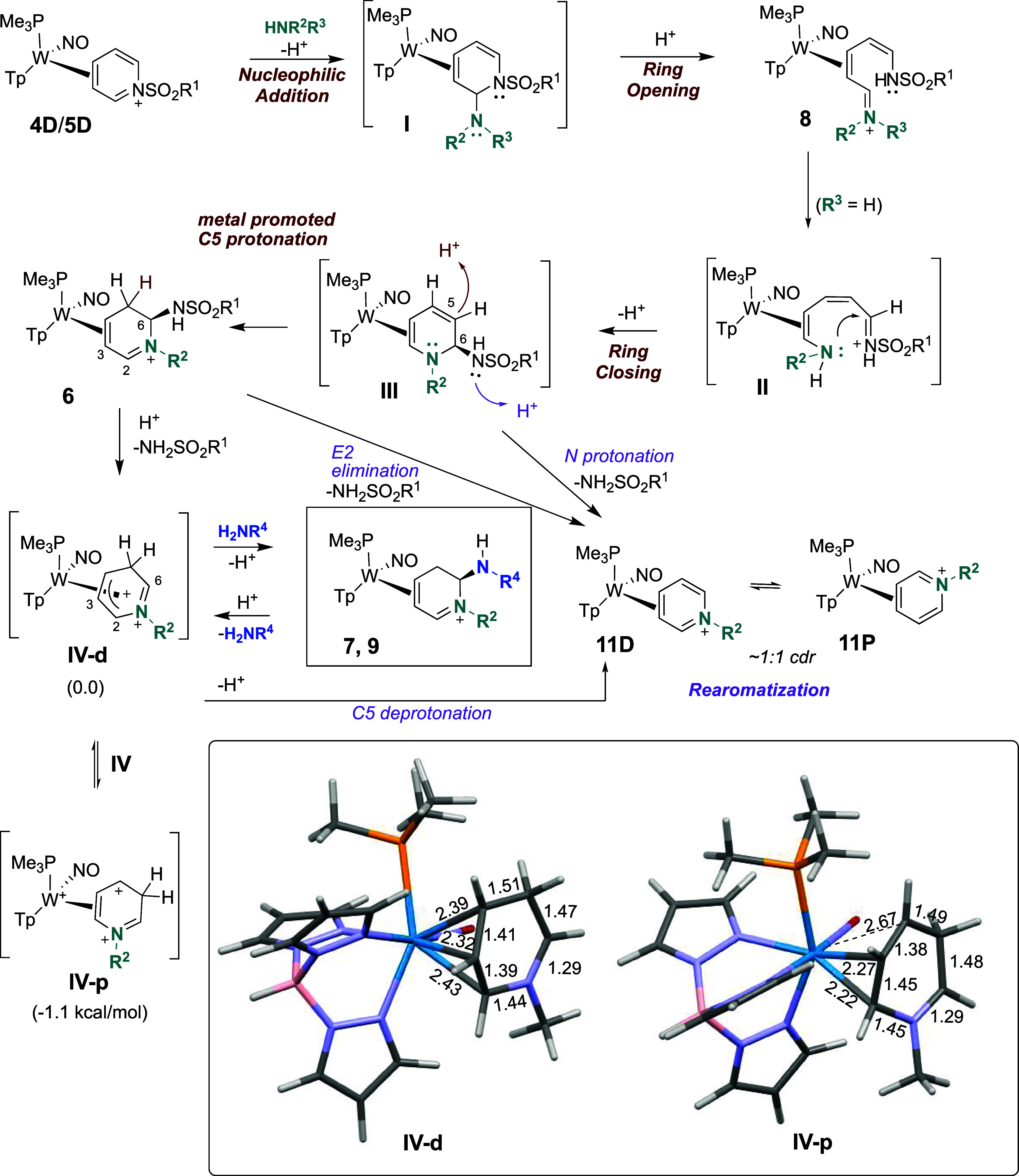
Proposed mechanism for the tungsten-promoted cycloreversion/cyclization process. Inset: optimized structure for dication **IV** in both proximal (p) and distal (d) conformations. R^1^ = Me, p-tol.

The mechanism for formation of polycyclic compounds (**7a**-**7g**) and double-amine incorporation (**9**) purportedly involves **III**, being protonated in the presence of acid, followed by the loss of the sulfonamidyl group (Figure 8). The resulting dicationic intermediate (**IV-d**; Figures 7 and 8) then undergoes ring-closure at the iminium C6 carbon anti to the metal followed by an allyl shift^16^ to generate a dihydropyridinium product (**7**). The proposed 5-endo-trig cyclization for **7a**, and **7d**–**f** would be considered contrary to Baldwin’s rules. However, 5-endo-trig cyclizations are commonly observed for cationic intermediates such as in cyclic acetal formation.^20^ The alternative 5-exo-tet cyclization, in which S_N_2 addition would likely result in addition syn to the metal, provides a bicyclic with the opposite stereochemistry relative to what is observed experimentally by crystallographic and NOESY data (see Figure 4).

**Figure 8:**
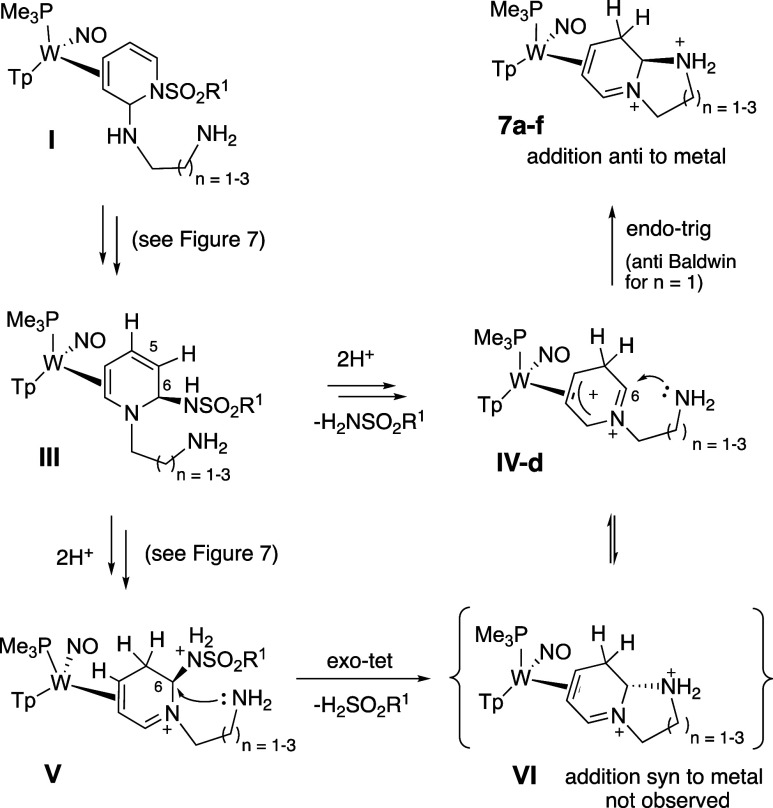
Intramolecular formation of bicyclic compounds **7a**–**f**.

In the course of optimizing the syntheses of **6a**-**6v**, correlations were observed between reaction conditions and various impurities proposed in the above mechanism (Figure 7; Tables S4–S6). For example, the syntheses of **6a** under varying conditions revealed the presence of a ring-opened impurity (similar to **8**), a double-addition impurity (**9**), and rearomatization impurities (similar to **11** *D* **/11P**). While colder temperatures, decreased nucleophile concentration, and shorter reaction times increased the presence of the ring-opening impurities, warmer temperatures, longer reaction times, and increased nucleophile concentration increased the prevalence of rearomatization and double-addition impurities. These trends generally hold across the various compounds **6a**-**6v**. A full summary of these optimizations is provided in the Supporting Information.

## Conclusions

Herein, we have described Zincke-like reactivity of an η^2^-bound *N*-sulfonylpyridinium complex of tungsten with a wide array of aliphatic amines. This one-pot, low-temperature ring-opening/ring-closing sequence is initiated by the addition of an amine to the pyridinium C2 carbon and is followed by the exchange of the sulfonylated ring nitrogen by the aliphatic amine nitrogen external to the heterocycle. Normally, the aminodihydropyridinium species resulting from this reaction sequence would eliminate back to a pyridinium salt. However, the influence of the tungsten dearomatization agent inhibits this process, allowing for the isolation of wide range of η^2^-(*N*-alkyl)­dihydropyridinium complexes. Furthermore, the sulfonamidyl group can be replaced with an array of pendant nucleophiles resulting in a secondary stereoselective ring-closure. The continued presence of the metal is critical for stabilizing these complexes and thus preventing their otherwise facile rearomatization. Preliminary attempts to decomplex the dihydropyridinium salts of type **6** and **7** have been unsuccessful, owing to the resistance of these compounds to being oxidized (the W­(I)/W(0) reduction potential is well over 1 V vs NHE).^12^ Experiments are underway to further elaborate compounds such as **7g** into quinolizidine complexes via nucleophilic addition to C2. These neutral complexes have much lower reduction potentials than the dihydropyridiniums, but present their own challenges as they are allylamine complexes.^12,13^ Efforts are also underway to effect intramolecular ring closures with a wider array of pendent electron-rich aromatics.

## Supplementary Material





## Notes

^1^H and ^13^C NMR spectra of selected compounds, crystallographic information for compounds **7a**, **7b** and **7e**, and DFT calculations for **IV** (PDF)
Atomic coordinates for the three structures (XYZ)
