# Supplementary material for: A Zincke-Inspired Cycloreversion/Cyclization Sequence with Arrested Rearomatization: Synthesis of 2‑Aminodihydropyridinium Complexes
Source: Organometallics. 2025 Aug 15;44(17):1920–5. doi: 10.1021/acs.organomet.5c00199 (PMC12421682; doi:10.1021/acs.organomet.5c00199)
Supplement: Supplementary file 1 [file om5c00199_si_001.pdf]

Supporting Information

A Zincke-Inspired Cycloreversion/Cyclization Sequence with Arrested Rearomatization: Synthesis of 2-Aminodihydropyridinium Complexes

Jonathan D. Dabbs,# Caleb C. Taylor,# Benjamin F. Livaudais, Alvin Q. Meng, Brian T. Quillin, Diane A. Dickie, and W. Dean Harman*

Department of Chemistry, University of Virginia, Charlottesville, Virginia, 22904

# authors contributed equally

Table of Contents

General Info (S1-S2)

**S1-S2**	**Supplementary Title Page, Table of Contents**
**S3**	**General Methods**
**S4**	**Abbreviations**

Compound Synthetic Methodologies and Characterizations (S5-S24)

**S5**	**Synthesis and Characterization of 4D and 5D**
**S6-S16**	**Synthesis and Characterization of 6a-6v**
**S17-S21**	**Synthesis and Characterization of 7a-7g**
**S22**	**Synthesis and Characterization of 8**
**S23**	**Synthesis and Characterization of 9**
**S24**	**Synthesis and Characterization of 10**

NMR Spectroscopy (S25-S57)

**S25**	**6a**	**^{1}H & ^{13}C NMRs (Figures S1 and S2)**
**S26**	**6b**	**^{1}H & ^{13}C NMRs (Figures S3 and S4)**
**S27**	**6c**	**^{1}H & ^{13}C NMRs (Figures S5 and S6)**
**S28**	**6d**	**^{1}H & ^{13}C NMRs (Figures S7 and S8)**
**S29**	**6e**	**^{1}H & ^{13}C NMRs (Figures S9 and S10)**
**S30**	**6f**	**^{1}H & ^{13}C NMRs (Figures S11 and S12)**
**S31**	**6g**	**^{1}H & ^{13}C NMRs (Figures S13 and S14)**
**S32**	**6h**	**^{1}H & ^{13}C NMRs (Figures S15 and S16)**
**S33**	**6i**	**^{1}H & ^{13}C NMRs (Figures S17 and S18)**
**S34**	**6j**	**^{1}H & ^{13}C NMRs (Figures S19 and S20)**
**S35**	**6k**	**^{1}H & ^{13}C NMRs (Figures S21 and S22)**
**S36**	**6l**	**^{1}H & ^{13}C NMRs (Figures S23 and S24)**
**S37**	**6m**	**^{1}H & ^{13}C NMRs (Figures S25 and S26)**
**S38**	**6n**	**^{1}H & ^{13}C NMRs (Figures S27 and S28)**
**S39**	**6o**	**^{1}H & ^{13}C NMRs (Figures S29 and S30)**
**S40**	**6p**	**^{1}H & ^{13}C NMRs (Figures S31 and S32)**
**S41**	**6q**	**^{1}H & ^{13}C NMRs (Figures S33 and S34)**
**S42**	**6r**	**^{1}H & ^{13}C NMRs (Figures S35 and S36)**
**S43**	**6s**	**^{1}H & ^{13}C NMRs (Figures S37 and S38)**
**S44**	**6t**	**^{1}H & ^{13}C NMRs (Figures S39 and S40)**
**S45**	**6u**	**^{1}H & ^{13}C NMRs (Figures S41 and S42)**
**S46**	**6v**	**^{1}H & ^{13}C NMRs (Figures S43 and S44)**
**S47**	**7a**	**^{1}H & ^{13}C NMRs (Figures S45 and S46)**

**S48**	**7b**	**^{1}H & ^{13}C NMRs (Figures S47 and S48)**
**S49**	**7c**	**^{1}H & ^{13}C NMRs (Figures S49 and S50)**
**S50**	**7d**	**^{1}H & ^{13}C NMRs (Figures S51 and S52)**
**S51**	**7e**	**^{1}H & ^{13}C NMRs (Figures S53 and S54)**
**S52**	**7f**	**^{1}H & ^{13}C NMRs (Figures S55 and S56)**
**S53**	**7g**	**^{1}H & ^{13}C NMRs (Figures S57 and S58)**
**S54**	**8**	**^{1}H & ^{13}C NMRs (Figures S59 and S60)**
**S55**	**9**	**^{1}H & ^{13}C NMRs (Figures S61 and S62)**
**S56**	**10**	**^{1}H & ^{13}C NMRs (Figures S63 and S64)**

SC-XRD Data (S57-S59)

**S57**	**7a**	**ORTEP Diagram & Data Table (Figure S65 and Table S1)**
**S58**	**7b**	**ORTEP Diagram & Data Table (Figure S66 and Table S2)**
**S59**	**7e**	**ORTEP Diagram & Data Table (Figure S67 and Table S3)**

Nitrogen Substitution Optimizations (S60-S63)

**S60**		**Overview and Mechanism (Figure S68)**
**S61**		**Conditions Screened for 6a-6e (Table S4)**
**S62**		**Conditions Screened for 6g-6p (Table S5)**
**S63**		**Conditions Screened for 6q-6v (Table S6)**
**S64**		**DFT calculations (Figure S69)**

References (S65)

General Methods:

NMR spectra were obtained on an 800 MHz spectrometer. Chemical shifts are referenced to tertramethylsilane (TMS) utilizing residual ^{1}H or ^{13}C signals of the deuterated solvents as internal standards. Chemical shifts are reported in ppm and coupling constants (*J*) are reported in hertz (Hz). Infrared Spectra (IR) were recorded on a spectrometer as a glaze on a diamond anvil ATR assembly, with peaks reported in cm^{-1}. Electrochemical experiments were performed under a nitrogen atmosphere. Most cyclic voltammetric (CV) data were recorded at ambient temperature at 100 mV/s, unless otherwise noted, with a standard three electrode cell from +1.25 V to -1.25 V with a platinum working electrode, *N,N*-dimethylacetamide (DMA) or acetonitrile solvent, and tetrabutylammonium hexafluorophosphate (TBAH) electrolyte (~1.0 M). All potentials are reported versus the normal hydrogen electrode (NHE) using cobaltocenium hexafluorophosphate ($E_{1/2}$ = -0.78 V, -1.75 V) or ferrocene ($E_{1/2}$ = 0.55 V) as an internal standard. Peak separation of all reversible couples was less than 100 mV. All synthetic reactions were performed in a glovebox under a dry nitrogen atmosphere unless otherwise noted. All solvents were purged with nitrogen prior to use. Deuterated solvents were used as received from Cambridge Isotopes. NMR samples prepared in a glovebox will typically have small toluene impurities in their NMR spectra owing to a -10 °C to -80 °C bath in which toluene is the coolant.

NMR assignments of all compounds were determined using 2D NMR methods including NOESY, COESY, HMBC, and HSQC. When possible, pyrazole (Pz) protons of the (trispyrazolyl) borate (Tp) ligand were uniquely assigned (e.g., “PzB3”) using two-dimensional NMR data (see Figure S1). If unambiguous assignments were not possible, Tp protons were labeled as “Pz3/5 or Pz4”. All *J* values for Pz protons are 2 (±0.4) Hz. BH peaks (around 4-5 ppm) in the ^{1}H NMR spectra are not assigned due to their quadrupole broadening; however, confirmation of the BH group is provided by IR data (around 2500 cm^{-1}).

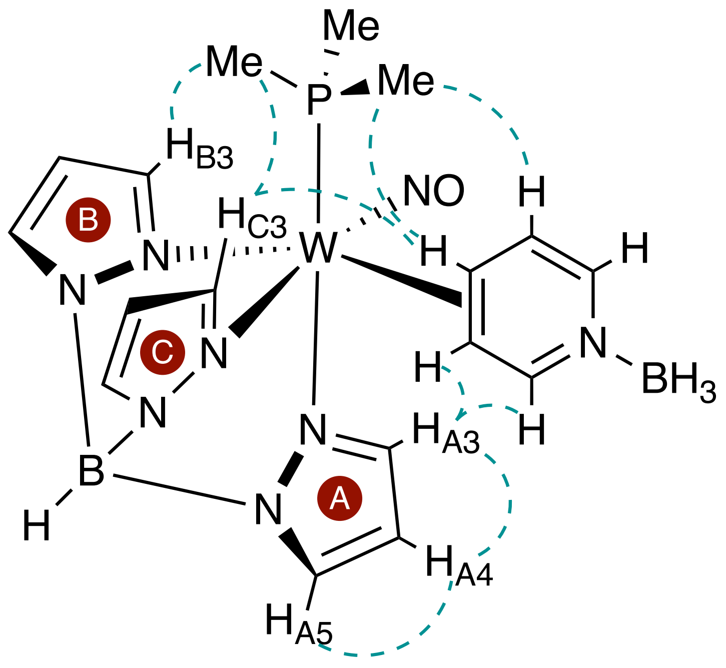

Abbreviations:

Anhydrous Magnesium Sulfate	$MgSO_4$
Acetonitrile	MeCN
Dichloromethane	DCM
Diethyl Ether	Et_2O
Dimethoxyethane	DME
Dimethylacetamide	DMA
Methanol	MeOH
Nitrosyl	NO
Normal Hydrogen Electrode	NHE
Propionitrile	EtCN
Saturated Aqueous Sodium Bicarbonate	$NaHCO_3$
Sodium Carbonate	Na_2CO_3
Sodium Cyanoborohydride	$NaCNBH_3$
Sodium Sulfonate	Na_2SO_4
Tetrahydrofuran	THF
Tetrabutylammonium Hexafluorophosphate	TBAH
Triethylamine	TEA
Trimethylphosphine	PMe_3
(Trispyrazolyl)borate	Tp
{TpW(NO)(PMe_3)}	[W]

COMPOUND SYNTHETIC METHODOLOGIES AND CHARACTERIZATIONS

Synthesis and characterization of WTp(NO)(PMe₃)(η^2-(*N*-mesyl)-pyridinium) (OTf) (4D)

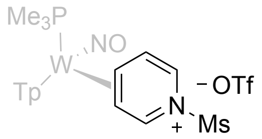

Reported Previously on S5 of Dabbs et al.[1]

Synthesis and characterization of WTp(NO)(PMe₃)(η^2-(*N*-tosyl)-pyridinium) (OTf) (5D)

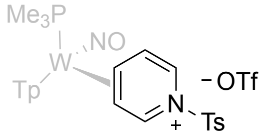

Reported Previously on S4 of Dabbs et al.[2]

Synthesis and Characterization of [W] Monosubstituted Complexes (6a-6v)

General Procedure

To an oven-dried 10 mL tube was added a stir pea, **4D** (50 mg), EtCN (1 mL), and DCM (3 mL). In a separate oven-dried 10 mL tube was added the desired primary amine (1.5 equivalents) and DCM (1 mL). Both solutions were chilled at -40 °C for 15 minutes, and then the nucleophile was added to the solution of **4D** dropwise. This solution was then stirred at -40 °C for 5 minutes, then precipitated into 100 mL of stirring Et_2O. A brown powder was precipitated, collected on a 15 mL F frit, washed 3x with 10 mL of Et_2O, and dried in a desiccator (64-87% yield).

WTp(NO)(PMe_3)(η^2-(*N*-methyl)-(*anti*-6-methanesulfonyl)-5,6-dihydropyridinium) (OTf) (6a)

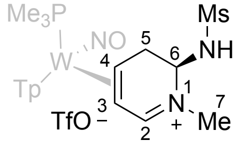

Prepared according to the General Procedure (40 mg, 73% yield).

^{1}H NMR (CD_3CN, δ, 25 °C): 8.84 (d, *J* = 5.1 Hz, 1H), 8.10 (d, *J* = 2.0 Hz, 1H), 7.99 (d, *J* = 2.0 Hz, 1H), 7.96 (m, 2H), 7.78 (d, *J* = 2.4 Hz, 1H), 7.73 (d, *J* = 2.1 Hz, 1H), 6.47 (t, *J* = 2.3 Hz, 1H), 6.41 (t, *J* = 2.3 Hz, 1H), 6.36 (t, *J* = 2.3 Hz, 1H), 5.24 (dd, *J* = 11.3, 6.4 Hz, 1H), 4.25 (m, 1H), 3.66 (s, 3H), 3.31 (m, 2H), 3.08 (s, 3H), 2.39 (dd, *J* = 5.8, 8.3 Hz, 1H), 1.20 (d, J_{PH} = 9.3 Hz, 9H).
^{13}C NMR (CD_3CN, δ, 25 °C): 182.4, 149.0, 145.4, 142.5, 139.3, 138.9, 137.7, 108.7, 107.9, 107.9, 67.3, 59.6 (d, J_{PC} = 15.4 Hz), 51.3, 42.5, 41.2, 38.1, 12.8 (d, J_{PC} = 31.5 Hz, 3C).
HRMS (ESI) Calcd for $C_{19}H_{32}BN_9O_3PSW^+$ ($[M]^+$) *m/z*: 692.1683, Found: 692.1689

WTp(NO)(PMe$_3$)(η^2-(*N*-propyl)-(*anti*-6-methanesulfonyl)-5,6-dihydropyridinium) (OTf) (6b)

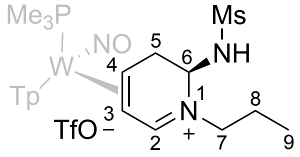

Prepared according to the General Procedure (44 mg, 82% yield).

^{1}H NMR (CD$_2$Cl$_2$, δ, 25 °C): 8.75 (d, *J* = 5.1 Hz, 1H), 8.06 (d, *J* = 1.9 Hz, 1H), 7.87 (m, 2H), 7.82 (d, *J* = 2.0 Hz, 1H), 7.78 (m, 1H), 6.45 (t, *J* = 2.2 Hz, 1H), 6.40 (t, *J* = 2.3 Hz, 1H), 6.34 (t, *J* = 2.3 Hz, 1H), 5.33 (m, 1H), 4.37 (m, 1H), 4.07 (m, 1H), 3.61 (m, 1H), 3.34 (m, 2H), 3.12 (s, 3H), 2.49 (dd, *J* = 5.3, 3.9 Hz, 1H), 2.00 (m, 1H), 1.82 (m, 1H), 1.23 (d, J_{PH} = 9.1 Hz, 9H), 1.05 (t, *J* = 7.4 Hz, 3H).
^{13}C NMR (CD$_2$Cl$_2$, δ, 25 °C): 180.4, 147.5, 144.3, 141.5, 138.4, 138.0, 136.8, 108.1, 107.5, 107.4, 65.8, 58.9 (d, J_{PC} = 15.6 Hz), 56.0, 51.4, 42.3, 37.8, 24.8, 13.0 (d, J_{PC} = 30.7 Hz, 3C), 11.5.
HRMS (ESI) Calcd for $C_{21}H_{36}BN_9O_3PSW^+$ ($[M]^+$) *m/z*: 720.1996, Found: 720.1995

WTp(NO)(PMe$_3$)(η^2-(*N-tert*butylmethyl)-(*anti*-6-methanesulfonyl)-5,6-dihydropyridinium) (OTf) (6c)

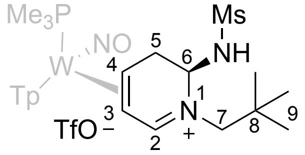

Prepared according to the General Procedure (41 mg, 77% yield).

^{1}H NMR (CD$_3$CN, δ, 25 °C): 8.77 (d, *J* = 4.1 Hz, 1H), 8.14 (d, *J* = 2.0 Hz, 1H), 7.97 (m, 2H), 7.89 (d, *J* = 2.0 Hz, 1H), 7.80 (d, *J* = 2.3 Hz, 1H), 7.71 (d, *J* = 2.1 Hz, 1H), 6.48 (t, *J* = 2.3 Hz, 1H), 6.43 (t, *J* = 2.3 Hz, 1H), 6.36 (t, *J* = 2.3 Hz, 1H), 5.02 (m, 1H), 3.82 (d, *J* = 13.8 Hz, 1H), 3.62 (dt, *J* = 4.9, 14.9 Hz, 1H), 3.60 (d, *J* = 13.8 Hz, 1H), 3.38 (m, 1H), 3.29 (m, 1H), 3.10 (s, 3H), 2.47 (dd, *J* = 4.2, 6.9 Hz, 1H), 1.18 (d, J_{PH} = 9.3 Hz, 9H), 1.10 (s, 9H).
^{13}C NMR (CD$_3$CN, δ, 25 °C): 184.5, 148.2, 145.4, 142.4, 139.1, 139.0, 137.9, 108.8, 108.1, 108.0, 68.4, 65.6, 54.4, 52.6 (d, J_{PC} = 15.7 Hz), 42.5, 35.3, 34.0, 27.7 (3C), 13.0 (d, J_{PC} = 31.5 Hz, 3C).
HRMS (ESI) Calcd for $C_{23}H_{40}BN_9O_3PSW^+$ ($[M]^+$) *m/z*: 748.2309, Found: 748.2290

WTp(NO)(PMe$_3$)(η^2-(*N*-allyl)-(*anti*-6-methanesulfonyl)-5,6-dihydropyridinium) (OTf) (6d)

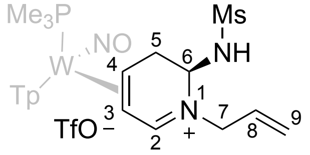

Prepared according to the General Procedure (39 mg, 72% yield).

^{1}H NMR (CD_2Cl_2, δ, 25 °C): 8.69 (d, *J* = 5.1 Hz, 1H), 8.06 (d, *J* = 1.9 Hz, 1H), 7.87 (d, *J* = 2.0 Hz, 1H), 7.86 (d, *J* = 2.3 Hz, 1H), 7.77 (d, *J* = 2.0 Hz, 1H), 7.69 (d, *J* = 2.1 Hz, 1H), 7.68 (d, *J* = 2.5 Hz, 1H), 6.45 (t, *J* = 2.3 Hz, 1H), 6.40 (t, *J* = 2.2 Hz, 1H), 6.31 (t, *J* = 2.3 Hz, 1H), 6.17 (m, 1H), 5.56 (d, *J* = 17.2 Hz, 1H), 5.50 (d, *J* = 10.1 Hz, 1H), 5.27 (dd, *J* = 6.0 Hz, 1H), 4.69 (dd, *J* = 6.2, 15.3 Hz, 1H) 4.42 (m, 2H), 3.36 (m, 2H), 3.11 (s, 3H), 2.58 (dd, *J* = 5.2, 7.2 Hz, 1H), 1.24 (d, J_{PH} = 9.1 Hz, 9H).
^{13}C NMR (CD_2Cl_2, δ, 25 °C): 179.7, 147.7, 144.4, 141.5, 138.4, 138.0, 136.9, 129.7, 122.7, 108.1, 107.5, 107.4, 66.4, 58.3 (d, J_{PC} = 15.3 Hz), 55.4, 52.2, 42.3, 37.6, 13.1 (d, J_{PC} = 30.8 Hz, 3C).
HRMS (ESI) Calcd for $C_{21}H_{34}BN_9O_3PSW^+$ ($[M]^+$) *m/z*: 718.1840, Found: 718.1848

WTp(NO)(PMe$_3$)(η^2-(*N*-propargyl)-(*anti*-6-methanesulfonyl)-5,6-dihydropyridinium) (OTf) (6e)

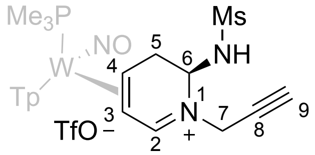

Prepared according to the General Procedure (33 mg, 66% yield).

^{1}H NMR (CD_3CN, δ, 25 °C): 9.15 (d, *J* = 5.4 Hz, 1H), 8.10 (d, *J* = 2.0 Hz, 1H), 8.04 (d, *J* = 2.0 Hz, 1H), 7.98 (d, *J* = 2.2 Hz, 1H), 7.97 (d, *J* = 2.3 Hz, 1H), 7.79 (d, *J* = 2.34 Hz, 1H), 7.76 (d, *J* = 2.1 Hz, 1H), 6.47 (t, *J* = 2.3 Hz, 1H), 6.43 (t, *J* = 2.3 Hz, 1H), 6.38 (t, *J* = 2.3 Hz, 1H), 5.32 (dd, *J* = 6.4, 11.0 Hz, 1H), 4.79 (dd, *J* = 2.5, 18.1 Hz, 1H), 4.69 (dd, *J* = 2.5, 18.1 Hz, 1H), 4.28 (dddd, *J* = 1.2, 4.5, 11.1, 14.5 Hz, 1H), 3.43 (m, 3.41-3.45, 1H), 3.36 (dd, *J* = 6.5, 14.5 Hz, 1H), 3.12 (s, 3H), 3.07 (t, *J* = 2.5 Hz, 1H), 2.58 (dd, *J* = 5.5, 8.1 Hz, 1H), 1.21 (d, J_{PH} = 9.4 Hz, 9H).
^{13}C NMR (CD_3CN, δ, 25 °C): 181.8, 149.1, 145.4, 142.4, 139.3, 139.1, 137.8, 108.7, 108.0 (2C), 79.3, 77.6, 67.0, 60.2 (d, J_{PC} = 15.3 Hz), 53.1, 42.4, 42.0, 38.5 (d, J_{PC} = 2.3 Hz), 12.8 (d, J_{PC} = 31.6 Hz, 3C).
HRMS (ESI) Calcd for $C_{21}H_{32}BN_9O_3PSW^+$ ($[M]^+$) *m/z*: 716.1689, Found: 716.1691

WTp(NO)(PMe$_3$)(η^2-(*N*-isopropyl)-(*anti*-6-methanesulfonyl)-5,6-dihydropyridinium) (OTf) (6f)

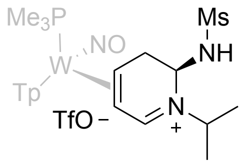

Prepared according to the General Procedure (86 mg, 80% yield).

^{1}H NMR (CD_3CN, δ, 25 °C): 8.97 (d, *J* = 5.2 Hz, 1H), 8.12 (d, *J* = 2.0 Hz, 1H), 7.98-7.97 (m, 2H), 7.85 (d, *J* = 2.0 Hz, 1H), 7.78 (d, *J* = 2.3 Hz, 1H), 7.76 (d, *J* = 2.1 Hz, 1H), 6.48 (t, *J* = 2.3 Hz, 1H), 6.42 (t, *J* = 2.3 Hz, 1H), 6.37 (t, *J* = 2.3 Hz, 1H), 5.25 (dd, *J* = 6.2, 10.9 Hz, 1H), 4.50 (spt, *J* = 6.7 Hz, 1H), 4.25 (m, 1H), 3.33 (m, 1H), 3.31 (m, 1H), 3.10 (s, 3H), 2.50 (dd, *J* = 5.7, 8.2 Hz, 1H), 1.58 (d, *J* = 6.8 Hz, 3H), 1.43 (d, *J* = 6.8 Hz, 3H), 1.19 (d, J_{PH} = 9.3 Hz, 9H).
^{13}C NMR (CD_3CN, δ, 25 °C): 178.8, 148.8, 145.2 (d, J_{PC} = 1.8 Hz), 142.5, 139.3, 138.9, 137.7, 108.7, 108.1, 107.9, 66.9, 59.1 (d, J_{PC} = 15.3 Hz), 52.6, 51.6, 42.4, 38.7, 24.7, 22.3, 12.7 (d, J_{PC} = 31.4 Hz, 3C).
HRMS (ESI) Calcd for $C_{21}H_{36}BN_9O_3PSW^+$ ($[M]^+$) *m/z*: 720.2002, Found: 720.1988

WTp(NO)(PMe$_3$)(η^2-(*N*-cyclohexyl)-(*anti*-6-methanesulfonyl)-5,6-dihydropyridinium) (OTf) (6g)

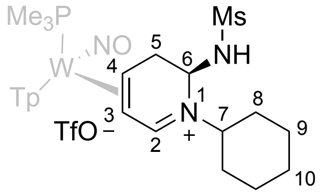

Prepared according to the General Procedure (41 mg, 69% yield).

^{1}H NMR (CD_3CN, δ, 25 °C): 8.93 (d, *J* = 5.2 Hz, 1H), 8.12 (d, *J* = 1.8 Hz, 1H), 7.97 (m, 2H), 7.86 (d, *J* = 1.8 Hz, 1H), 7.78 (d, *J* = 2.2 Hz, 1H), 7.75 (d, *J* = 1.8 Hz, 1H), 6.47 (t, *J* = 2.2 Hz, 1H), 6.42 (t, *J* = 2.2 Hz, 1H), 6.37 (t, *J* = 2.1 Hz, 1H), 5.24 (dd, *J* = 6.2, 10.6 Hz, 1H), 4.25 (ddd, *J* = 5.3, 11.4, 13.8 Hz, 1H), 4.06 (tt, *J* = 3.3, 12.2 Hz, 1H), 3.31 (m, 2H), 3.10 (s, 3H), 2.47 (dd, *J* = 5.8, 7.5 Hz), 2.39 (m, 1H), 2.00 (m, 1H), 1.90 (m, 2H), 1.66 (m, 3H), 1.37 (m, 3H), 1.18 (d, J_{PC} = 9.2 Hz, 9H).
^{13}C NMR (CD_3CN, δ, 25 °C): 179.1, 148.8, 145.2, 142.5, 139.3, 138.9, 137.8, 108.7, 108.1, 107.9, 67.0, 60d.3, 59.1 (d, J_{PC} = 15.4 Hz), 51.4, 42.4, 38.7, 35.9, 33.5, 26.8, 26.5, 24.9, 12.6 (d, *J* = 31.4 Hz, 3C).
^{31}P NMR (CD_3CN, δ, 25 °C): -8.20 (J_{WP} = 277 Hz).
HRMS (ESI) Calcd for $C_{24}H_{40}BN_9O_3PSW^+$ ($[M]^+$) *m/z*: 760.2309, Found: 760.2305

WTp(NO)(PMe$_3$)(η^2-(*N*-(triethoxysilyl)propyl)-(*anti*-6-methanesulfonyl)-5,6-dihydropyridinium) (OTf) (6h)

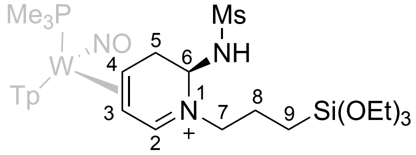

Prepared according to the General Procedure (47 mg, 73% yield).

^{1}H NMR (CDCl$_3$, δ, 25 °C): 8.77 (1H, d, *J* = 4.2 Hz), 8.06 (1H, d, *J* = 1.4 Hz), 7.89 (1H, d, *J* = 1.5 Hz), 7.82 (1H, d *J* = 1.7 Hz), 7.81 (1H, d, *J* = 1.4 Hz), 7.74 (1H, d, *J* = 1.7 Hz), 7.62 (1H, d, *J* = 1.7 Hz), 6.42 (1H, t *J* = 1.9 Hz), 6.39 (1H, t, *J* = 1.9 Hz), 6.28 (1H, t, *J* = 1.8 Hz), 5.28 (1H, dd, *J* = 1.3, 8.8 Hz), 4.34 (1H, m), 4.18 (1H, m), 3.81 (6H, q, *J* = 6.9 Hz), 3.72 (1H, m), 3.38 (2H, m), 3.17 (3H, s), 2.47 (1H, dd, *J* = 5.0, 7.2 Hz), 2.08 (1H, m), 1.93 (1H, m), 1.22 (9H, d, J_{PH} = 8.9 Hz), 1.19 (9H, t, *J* = 7.0 Hz), 0.77 (2H, t, *J* = 7.8 Hz).
^{13}C NMR (CDCl$_3$, δ, 25 °C): 180.2, 147.4, 144.1, 141.6, 137.9, 137.5, 136.4, 107.8, 107.4, 107.2, 65.3, 58.7 (3C), 57.5 (d, J_{PC} = 15.5 Hz), 55.3, 51.4, 42.1, 37.0, 24.6, 18.4 (3C), 13.1 (d, J_{PC} = 30.6 Hz, 3C), 7.6.
HRMS (ESI) Calcd for $C_{27}H_{50}BN_9O_3PSSiW^+$ ([M]$^+$) *m/z*: 882.2709, Found: 882.2717

WTp(NO)(PMe$_3$)(η^2-(*N*-2-(tert-butoxy)-2-oxoethyl)-(*anti*-6-methanesulfonyl)-5,6-dihydropyridinium) (OTf) (6i)

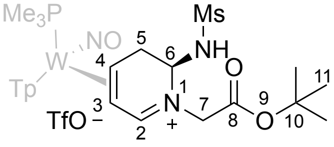

Prepared according to the General Procedure (46 mg, 72% yield).

^{1}H NMR (CD$_3$CN, δ, 25 °C): 8.89 (d, *J* = 5.6 Hz, 1H), 8.17 (d, *J* = 1.9 Hz, 1H), 8.11 (d, *J* = 1.8 Hz, 1H), 7.98 (m, 2H), 7.78 (d, *J* = 2.4 Hz, 1H), 7.75 (d, *J* = 2.1 Hz, 1H), 6.48 (t, *J* = 2.2 Hz, 1H), 6.44 (t, *J* = 2.2 Hz, 1H), 6.33 (t, *J* = 2.2 Hz, 1H), 5.26 (dd, *J* = 11.7, 6.2 Hz, 1H), 4.60 (d , *J* = 17.4 Hz, 1H), 4.52 (d, *J* = 17.4 Hz, 1H), 4.20 (m, 1H), 3.48 (m, 1H), 3.30 (dd, *J* = 14.6, 6.2 Hz, 1H), 3.06 (s, 3H), 2.53 (t, *J* = 6.56, 1H), 1.49 (s, 9H), 1.19 (d, J_{PH} = 9.3, 9H).
^{13}C NMR (CD$_3$CN, δ, 25 °C): 185.3, 168.5, 149.6, 145.2, 142.4, 139.3, 139.1, 137.8, 108.8, 108.1, 107.8, 84.0, 67.2, 61.7 (d, J_{PC} = 15.7 Hz), 54.2, 52.7, 42.0, 38.2, 28.1 (3C), 12.6 (d, J_{PC} = 31.1 Hz, 3C).
HRMS (ESI) Calcd for $C_{21}H_{36}BN_9O_3PSW^+$ ([M]$^+$) *m/z*: 792.2213, Found: 792.2211

WTp(NO)(PMe$_3$)(η^2-(*N*-benzyl)-(*anti*-6-methanesulfonyl)-5,6-dihydropyridinium) (OTf) (6j)

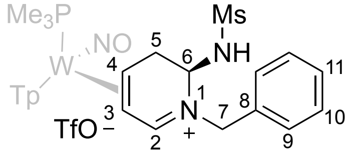

Prepared according to the General Procedure (44 mg, 84% yield).

^{1}H NMR (CD$_3$CN, δ, 25 °C): 8.62 (d, *J* = 5.3 Hz, 1H), 8.11 (d, *J* = 1.9 Hz, 1H), 7.94 (d, *J* = 2.3 Hz, 1H), 7.93 (d, *J* = 2.2 Hz, 1H), 7.72 (d, *J* = 1.9 Hz, 1H), 7.69 (d, *J* = 2.3 Hz, 1H), 7.57 (d, *J* = 7.7 Hz, 2H), 7.47 (m, 3H), 6.89 (d, *J* = 1.8 Hz, 1H), 6.46 (t, *J* = 2.2 Hz, 1H), 6.39 (t, *J* = 2.3 Hz, 1H), 6.09 (t, *J* = 2.2 Hz, 1H), 5.41 (dd, *J* = 6.2, 10.8 Hz, 1H), 5.21 (d, *J* = 14.7 Hz, 1H), 4.98 (d, *J* = 14.7 Hz, 1H), 4.31-4.35 (m, 1H), 3.35-3.39 (m, 2H), 3.07 (s, 3H), 2.37 (dd, *J* = 5.8, 7.4 Hz, 1H), 1.20 (d, J_{PH} = 9.3 Hz, 9H).
^{13}C NMR (CD$_3$CN, δ, 25 °C): 181.8, 148.7, 145.3, 142.5, 139.3, 139.0, 137.6, 135.8, 131.3 (2C), 130.4 (2C), 130.1, 108.7, 108.0, 107.6, 67.9, 59.6, 55.7, 52.0, 42.4, 38.5, 12.8 (d, J_{PC} = 31.6 Hz, 3C).
HRMS (ESI) Calcd for $C_{25}H_{36}BN_9O_3PSW^+$ ([M]$^+$) *m/z*: 768.1996, Found: 768.2002

WTp(NO)(PMe$_3$)(η^2-(*N*-3,5-dimethoxybenzyl)-(*anti*-6-methanesulfonyl)-5,6-dihydropyridinium) (OTf) (6k)

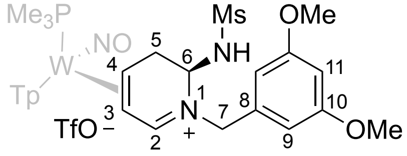

Prepared according to the General Procedure (37 mg, 64% yield).

^{1}H NMR (CD$_3$CN, δ, 25 °C): 8.65 (d, *J* = 5.3 Hz, 1H), 8.11 (d, *J* = 1.9 Hz, 1H), 7.95 (d, *J* = 2.3 Hz, 1H), 7.94 (d, *J* = 2.2 Hz, 1H), 7.71 (d, *J* = 2.0 Hz, 1H), 7.70 (d, *J* = 2.4 Hz, 1H), 6.96 (d, *J* = 1.8 Hz, 1H), 6.73 (d, *J* = 2.2 Hz, 2H), 6.56 (t, *J* = 2.2 Hz, 1H), 6.48 (t, *J* = 2.3 Hz, 1H), 6.40 (t, *J* = 2.3 Hz, 1H), 6.11 (t, *J* = 2.2 Hz, 1H), 5.42 (dd, *J* = 6.2, 10.9 Hz, 1H), 5.11 (d, *J* = 14.5 Hz, 1H), 4.80 (d, *J* = 14.6 Hz, 1H), 4.24-4.29 (m, 2H), 3.76 (s, 6H), 3.33-3.39 (m, 2H), 3.10 (s, 3H), 2.40 (dd, *J* = 5.7, 7.8 Hz, 1H), 1.19 (d, J_{PC} = 9.3 Hz).
^{13}C NMR (CD$_3$CN, δ, 25 °C): 181.9, 162.6 (2C), 148.8, 145.2, 142.4, 139.3, 139.0, 138.0, 137.6, 109.1 (2C), 108.7, 108.0, 107.5, 101.4, 67.8, 59.5 (d, J_{PC} = 15.9 Hz), 56.1 (2C), 55.6, 52.0, 42.5, 38.6, 12.7 (d, J_{PC} = 31.5 Hz).
HRMS (ESI) Calcd for $C_{27}H_{40}BN_9O_5PSW^+$ ([M]$^+$) *m/z*: 828.2208, Found: 828.2207

WTp(NO)(PMe$_3$)(η^2-(*N*-methylbenzodioxolyl)-(*anti*-6-methanesulfonyl)-5,6-dihydropyridinium) (OTf) (6l)

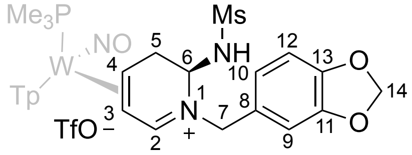

Prepared according to the General Procedure (42 mg, 71% yield).

^{1}H NMR (CD$_2$Cl$_2$, δ, 25 °C): 8.33 (d, J = 5.0 Hz, 1H), 8.06 (d, J = 1.9 Hz, 1H), 7.85 (d, J = 2.0 Hz, 1H), 7.83 (d, J = 2.2 Hz, 1H) 7.65 (d, J = 2.1 Hz, 1H), 7.59 (d, J = 2.4 Hz, 1H), 7.11 (d, J = 1.7 Hz, 1H), 7.08 (dd, J = 1.7, 7.8 Hz, 1H), 6.97 (d, J = 1.8 Hz, 1H), 6.93 (d, J = 7.8 Hz, 1H), 6.46 (t, J = 2.2 Hz, 1H), 6.37 (t, J = 2.3 Hz, 1H), 6.08 (t, J = 2.3 Hz, 1H), 6.02 (d, J = 1.3 Hz, 1H), 6.00 (d, J = 1.3 Hz, 1H), 5.38 (dd, J = 6.6, 9.8 Hz, 1H), 5.09 (d, J = 14.5 Hz, 1H), 5.04 (d, J = 14.5 Hz, 1H), 4.50 (ddd, J = 4.7, 10.5, 14.7 Hz, 1H), 3.37 (dd, J = 6.3, 14.5 Hz, 1H), 3.28 (m, 1H), 3.11 (s, 3H), 2.45 (dd, J = 5.4, 8.2 Hz, 1H), 1.24 (d, J_{PH} = 8.9 Hz, 9H).
^{13}C NMR (CD$_2$Cl$_2$, δ, 25 °C): 178.5, 149.2, 149.1, 147.4, 144.6, 141.5, 138.4, 138.0, 136.7, 126.9, 125.7, 111.5, 109.4, 108.1, 107.4, 106.9, 102.2, 67.6, 57.8 (d, J_{PC} = 15.3 Hz), 55.7, 51.3, 42.3, 37.9, 13.2 (d, J_{PC} = 30.8 Hz, 3C).
HRMS (ESI) Calcd for $C_{26}H_{36}BN_9O_5PSW^+$ ($[M]^+$) *m/z*: 812.1895, Found: 812.1909

WTp(NO)(PMe$_3$)(η^2-(*N*-methylpyridyl)-(*anti*-6-methanesulfonyl)-5,6-dihydropyridinium) (OTf) (6m)

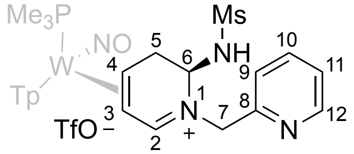

Prepared according to the General Procedure (52 mg, 91% yield).

^{1}H NMR (CD$_3$CN, δ, 25 °C): 9.18 (1H, d, J = 5.4 Hz), 8.61 (1H, d, J = 1.9 Hz), 8.56 (1H, d, J = 4.5 Hz), 8.03 (1H, d, J = 1.9 Hz), 7.98 (2H, m), 7.78 (1H, d, J = 2.3 Hz), 7.76 (1H, td, J = 1.8, 7.7 Hz), 7.74 (1H, d, J = 2.1 Hz), 7.48 (1H, d, J = 7.7 Hz), 7.32 (1H, dd, J = 4.8, 7.4 Hz), 6.46 (1H, t, J = 2.3 Hz), 6.41 (1H, t, J = 2.2 Hz), 6.38 (1H, t, J = 2.3 Hz), 5.27 (1H, d, J = 15.2 Hz), 5.21 (1H, dd, J = 6.2, 11.3 Hz), 4.95 (1H, d, J = 15.2 Hz), 4.24 (1H, dd, J = 1.2, 4.8 Hz), 3.41 (1H, m), 3.34 (1H, dd, J = 6.3, 14.5 Hz), 3.01 (3H, s), 2.49 (2H, m), 1.16 (9H, d, J_{PH} = 9.2 Hz).
^{13}C NMR (CD$_3$CN, δ, 25 °C): 185.0, 155.0, 150.3, 149.2, 145.0, 142.3, 139.2, 138.9, 138.3, 137.6, 124.6, 124.5, 108.6, 107.9, 107.8, 66.6, 61.2 (d, J_{PC} = 15.5 Hz), 56.8, 52.0, 42.3, 38.9, 12.6 (d, J_{PC} = 31.3 Hz, 3C).
HRMS (ESI) Calcd for $C_{24}H_{35}BN_{10}O_3PSW^+$ ($[M]^+$) *m/z*: 769.1955, Found: 769.1954

WTp(NO)(PMe$_3$)(η^2-(*N*-methylfuranyl)-(*anti*-6-methanesulfonyl)-5,6-dihydropyridinium) (OTf) (6n)

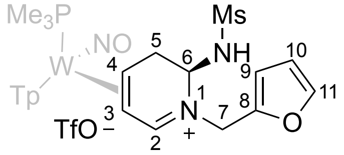

Prepared according to the General Procedure (48 mg, 84% yield).

^{1}H NMR (CD$_3$CN, δ, 25 °C): 8.81 (d, *J* = 5.4 Hz, 1H), 8.06 (d, *J* = 2.1 Hz, 1H), 7.96 (d, *J* = 2.4 Hz, 1H), 7.95 (d, *J* = 2.5 Hz, 1H), 7.76 (d, *J* = 2.4 Hz, 1H), 7.75 (d, *J* = 2.1 Hz, 1H), 7.74 (d, *J* = 2.1 Hz, 1H), 7.59 (dd, *J* = 1.8, 0.7 Hz, 1H), 6.63 (d, *J* = 3.3 Hz, 1H), 6.47 (dd, *J* = 1.9, 3.2 Hz, 1H), 6.45 (t, *J* = 2.3 Hz, 1H), 6.41 (t, *J* = 2.2 Hz, 1H), 6.35 (t, *J* = 2.4 Hz, 1H), 5.31 (dd, *J* = 10.8, 6.4 Hz, 1H), 5.20 (d, *J* = 15.7 Hz, 1H), 4.97 (d, *J* = 15.7 Hz, 1H), 4.28 (dd, *J* = 1.9, 5.2 Hz, 1H), 3.38 (m, 2H), 3.11 (s, 3H), 2.46 (dd, *J* = 5.9, 7.7 Hz, 1H), 1.18 (d, J_{PH} = 9.3 Hz, 9H).
^{13}C NMR (CD$_3$CN, δ, 25 °C): 182.4, 148.9, 148.8, 145.3, 145.2, 142.5, 139.3, 139.0, 137.7, 113.0, 112.2, 108.7, 108.0, 107.9, 66.9, 60.3 (d, J_{PC} = 15.6 Hz), 52.4, 48.4, 42.5, 38.7, 12.8 (d, J_{PC} = 31.3 Hz, 3C).
HRMS (ESI) Calcd for $C_{23}H_{34}BN_9O_4PSW^+$ ([M]$^+$) *m/z*: 758.1789, Found: 758.1788

WTp(NO)(PMe$_3$)(η^2-(*N*-methylindolyl)-(*anti*-6-methanesulfonyl)-5,6-dihydropyridinium) (OTf) (6o)

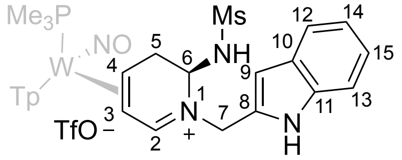

Prepared according to the General Procedure (40 mg, 67% yield).

^{1}H NMR (CD$_3$CN, δ, 25 °C): 9.82 (br s, 1H), 8.69 (d, *J* = 5.2 Hz, 1H), 8.09 (d, *J* = 2.0 Hz, 1H), 7.91 (d, *J* = 2.3 Hz, 2H), 7.69 (d, *J* = 2.3 Hz, 1H), 7.64 (d, *J* = 7.8 Hz, 1H), 7.58 (d, *J* = 2.5 Hz, 1H), 7.33 (dd, *J* = 0.7, 8.2 Hz, 1H), 7.16 (m, 1H), 7.09 (td, *J* = 0.9, 7.7 Hz, 1H), 6.78 (s, 1H), 6.75 (d, *J* = 1.7 Hz, 1H), 6.46 (t, *J* = 2.4 Hz, 1H), 6.39 (t, *J* = 2.3 Hz, 1H), 5.67 (t, *J* = 2.2 Hz, 1H), 5.44 (dd, *J* = 6.5, 9.9 Hz, 1H), 5.34 (d, *J* = 15.3 Hz, 1H), 5.1 (d, *J* = 15.3 Hz, 1H), 4.32 (m, 1H), 3.38 (dd, *J* = 1.6, 4.5 Hz, 1H), 3.35 (dd, *J* = 6.5, 15.1 Hz, 1H), 3.15 (s, 3H), 2.43 (dd, *J* = 5.6, 7.4 Hz, 1H), 1.20 (d, J_{PH} = 9.4 Hz, 9H).
^{13}C NMR (CD$_3$CN, δ, 25 °C): 181.6, 148.6, 145.4, 142.4, 139.3, 138.9, 138.1, 137.5, 132.7, 128.8, 123.8, 121.6, 121.0, 112.6, 108.7, 108.0, 107.3, 106.0, 67.5, 59.5 (d, J_{PC} = 15.4 Hz), 52.5, 48.5, 42.5, 38.3, 12.9 (d, J_{PC} = 31.5 Hz, 3C).
HRMS (ESI) Calcd for $C_{27}H_{37}BN_{10}O_3PSW^+$ ([M]$^+$) *m/z*: 807.2105, Found: 807.2073

WTp(NO)(PMe$_3$)(η^2-(*N*-phenethyl)-(*anti*-6-methanesulfonyl)-5,6-dihydropyridinium) (OTf) (6p)

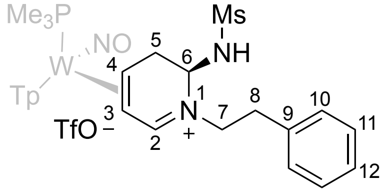

Prepared according to the General Procedure (44 mg, 71% yield).

^{1}H NMR (CD$_3$CN, δ, 25 °C): 8.88 (d, *J* = 5.3 Hz, 1H), 8.13 (d, *J* = 2.0 Hz, 1H), 7.96 (m, 2H), 7.76 (d, *J* = 2.4 Hz, 1H), 7.73 (d, *J* = 2.2 Hz, 1H), 7.70 (d, *J* = 2.0 Hz, 1H), 7.37 (d, *J* = 7.5 Hz, 2H), 7.34 (t, *J* = 7.6 Hz, 2H), 7.15 (t, *J* = 7.3 Hz, 1H), 6.48 (t, *J* = 2.3 Hz, 1H), 6.42 (t, *J* = 2.3 Hz, 1H), 6.31 (t, *J* = 2.3 Hz, 1H), 5.38 (dd, *J* = 6.3, 11.2 Hz, 1H), 4.24 (ddd, *J* = 4.7, 11.4, 13.9 Hz, 1H), 4.18 (ddd, *J* = 5.7, 10.3, 14.0 Hz, 1H), 3.99 (ddd, *J* = 6.2, 10.5, 13.9 Hz, 1H), 3.35 (m, 2H), 3.26 (ddd, *J* = 5.6, 10.4, 13.6 Hz, 1H), 3.13 (s, 3H), 3.08 (m, 1H), 2.39 (dd, *J* = 5.6, 7.6 Hz ,1H), 1.19 (d, J_{PH} = 9.3 Hz, 9H).
^{13}C NMR (CD$_3$CN, δ, 25 °C): 182.3, 149.3, 145.3, 142.4, 139.3, 139.0, 138.6, 130.5 130.0 (2C), 129.9, 129.7 (2C), 108.8, 108.0, 108.0, 66.4, 60.3 (d, J_{PC} = 15.4 Hz), 54.5, 51.8, 42.8, 38.4, 37.6, 12.8 (d, J_{PC} = 31.2 Hz, 3C).
HRMS (ESI) Calcd for $C_{26}H_{38}BN_9O_3PSW^+$ ([M]$^+$) *m/z*: 782.2153, Found: 782.2159

WTp(NO)(PMe$_3$)(η^2-(*N*-methoxyphenethyl)-(*anti*-6-methanesulfonyl)-5,6-dihydropyridinium) (OTf) (6q)

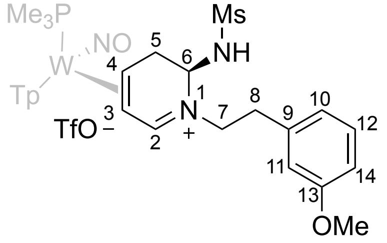

Prepared according to the General Procedure (47 mg, 83% yield).

^{1}H NMR (CD$_3$CN, δ, 25 °C): 8.84 (d, *J* = 5.4 Hz, 1H), 8.13 (d, *J* = 1.9 Hz, 1H), 7.96 (d, *J* = 2.2 Hz, 1H), 7.96 (d, *J* = 2.3 Hz, 1H), 7.75 (d, *J* = 2.3 Hz, 1H), 7.73 (d, *J* = 2.0 Hz, 1H), 7.60 (d, *J* = 1.9 Hz, 1H), 7.24 (m, 1H), 6.98 (s, 1H), 6.94 (d, *J* = 7.7 Hz, 1H), 6.81 (dd, *J* = 2.5, 8.3 Hz, 1H), 6.47 (t, *J* = 2.2 Hz, 1H), 6.41 (t, *J* = 2.2 Hz, 1H), 6.29 (t, *J* = 2.2 Hz, 1H), 5.39 (dd, *J* = 6.1, 10.9 Hz, 1H), 4.24 (m, 1H), 4.20 (ddd, *J* = 6.6, 10.0, 13.1 Hz, 1H), 3.98 (ddd *J* = 6.5, 9.7, 14.1 Hz, 1H), 3.73 (s, 3H), 3.32 (m, 2H), 3.22 (ddd *J* = 6.6, 9.6, 13.6 Hz, 1H), 3.14 (3H, s), 3.04 (ddd *J* = 5.8, 9.8, 13.5 Hz, 1H), 2.36 (dd, *J* = 5.8. 7.3 Hz, 1H), 1.19 (d, J_{PH} = 9.2 Hz, 9H).
^{13}C NMR (CD$_3$CN, δ, 25 °C): 182.3, 160.9, 149.2, 145.1, 142.4, 140.2, 139.3, 138.9, 137.6, 130.7, 122.0, 115.7, 113.5, 108.7, 107.9, 107.9, 66.3, 60.3 (d, J_{PC} = 15.5 Hz), 55.7, 54.5, 51.6, 42.8, 38.3, 37.5, 12.7 (d, J_{PC} = 31.5 Hz, 3C).
HRMS (ESI) Calcd for $C_{27}H_{40}BN_9O_4PSW^+$ ([M]$^+$) *m/z*: 812.2258, Found: 812.2251
CV (MeCN; 50 mV/s; FeCp$_2$ standard): $E_{p,c}$ = -1403 mV (NHE)

WTp(NO)(PMe$_3$)(η^2-(*N*-ethylfuranyl)-(*anti*-6-methanesulfonyl)-5,6-dihydropyridinium) (OTf) (6r)

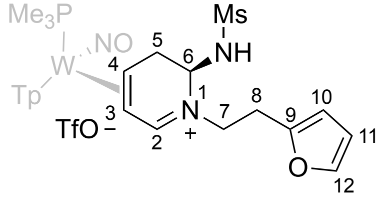

Prepared according to the General Procedure (43 mg, 78% yield).

^{1}H NMR (CD$_3$CN, δ, 25 °C): 8.80 (d, *J* = 5.3 Hz, 1H), 8.13 (d, *J* = 1.8 Hz, 1H), 7.97 (d, *J* = 2.2 Hz, 1H), 7.96 (d, *J* = 2.1 Hz, 1H), 7.76 (d, *J* = 2.6 Hz, 1H), 7.74 (d, *J* = 1.9 Hz, 1H), 7.71 (d, *J* = 1.8 Hz, 1H), 7.46 (d, *J* = 1.2 Hz, 1H), 6.48 (t, *J* = 2.3 Hz, 1H), 6.41 (t, *J* = 2.5 Hz, 1H), 6.36 (dd, *J* = 1.9, 2.9 Hz, 1H), 6.31 (d, *J* = 2.2 Hz, 1H), 6.26 (d, *J* = 3.0 Hz, 1H), 5.32 (dd, *J* = 10.5, 16.8 Hz, 1H), 4.25 (m, 2H), 4.21 (m, 1H), 4.05 (ddd, *J* = 6.3, 8.6, 14.0 Hz, 1H), 3.35 (m, 1H), 3.30 (dd, *J* = 6.4, 15.0 Hz, 1H), 3.25 (ddd, *J* = 6.0, 8.7, 15.8 Hz, 1H), 3.15 (ddd, *J* = 6.8, 8.4, 15.2 Hz, 1H), 3.12 (s, 3H), 2.41 (t, *J* = 6.5, 1H), 1.19 (d, J_{PH} = 9.3 Hz, 9H).
^{13}C NMR (CD$_3$CN, δ, 25 °C): 182.6, 152.5, 149.3, 145.3, 143.2, 142.5, 139.4, 139.0, 137.7, 111.7, 108.8, 108.5, 108.0 (2C), 66.4, 60.5 (d, J_{CP} = 15.6 Hz), 51.9 (2C), 42.7, 38.5, 30.3, 12.7 (d, J_{PC} = 31.5 Hz),
HRMS (ESI) Calcd for $C_{24}H_{36}BN_9O_4PSW^+$ ($[M]^+$) *m/z*: 772.1951, Found: 772.1953

WTp(NO)(PMe$_3$)(η^2-(*N*-ethylthienyl)-(*anti*-6-methanesulfonyl)-5,6-dihydropyridinium) (OTf) (6s)

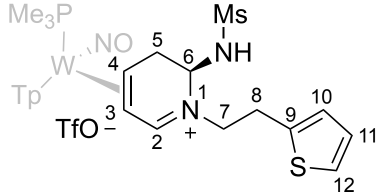

Prepared according to the General Procedure (43 mg, 78% yield).

^{1}H NMR (CD$_3$CN, δ, 25 °C): 8.86 (d, *J* = 5.5 Hz, 1H), 8.13 (d, *J* = 1.9 Hz, 1H), 7.97 (d, *J* = 2.1 Hz, 1H), 7.96 (d, *J* = 2.3 Hz, 1H), 7.75 (d, *J* = 2.2 Hz, 1H), 7.74 (d, *J* = 1.8 Hz, 1H), 7.64 (d, *J* = 1.9 Hz, 1H), 7.28 (d, *J* = 5.1 Hz, 1H), 7.02 (d, *J* = 3.3 Hz, 1H), 6.98 (dd, *J* = 3.7, 5.0 Hz, 1H), 6.48 (t, *J* = 2.3 Hz, 1H), 6.42 (t, *J* = 2.3 Hz, 1H), 6.30 (t, *J* = 2.3 Hz, 1H), 5.35 (dd, *J* = 5.4, 10.7 Hz, 1H), 4.24 (m, 2H), 4.02 (ddd, *J* = 6.5, 9.4, 14.1 Hz, 1H), 3.45 (ddd, *J* = 5.8, 9.5, 15.0 Hz, 1H), 3.34 (m, 3H), 3.12 (s, 3H), 2.41 (t, *J* = 6.6 Hz, 1H), 1.19 (d, J_{PH} = 9.9 Hz, 9H).
^{13}C NMR (CD$_3$CN, δ, 25 °C): 182.5, 149.3, 145.3, 142.5, 140.6, 139.3, 139.0, 137.7, 128.4, 127.4, 125.8, 108.7, 108.0 (2C), 66.4, 60.5 (d, J_{PC} = 15.5 Hz), 54.6, 51.8, 42.7, 38.4, 31.9, 12.7 (d, J_{PC} = 31.3 Hz).
HRMS (ESI) Calcd for $C_{24}H_{36}BN_9O_3PS_2W^+$ ($[M]^+$) *m/z*: 788.1717, Found: 788.1708

WTp(NO)(PMe$_3$)(η^2-(*N*-ethylindolyl)-(*anti*-6-methanesulfonyl)-5,6-dihydropyridinium) (OTf) (6t)

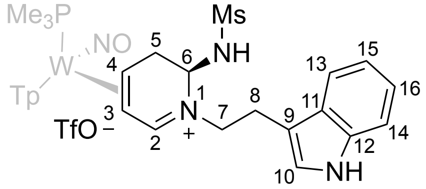

Prepared according to the General Procedure (47 mg, 79% yield).

^{1}H NMR (CD$_3$CN, δ, 25 °C): 8.73 (d, *J* = 5.2 Hz, 1H), 8.14 (d, *J* = 2.0 Hz, 1H), 7.96 (d, *J* = 2.4 Hz, 1H), 7.94 (d, *J* = 2.3 Hz, 1H), 7.71 (d, *J* = 2.4 Hz, 1H), 7.70 (d, *J* = 2.2 Hz, 1H), 7.69 (ddd, *J* = 0.77, 1.07, 7.88 Hz, 1H), 7.42 (dd, *J* = 0.9, 8.2 Hz,1H), 7.30 (d, *J* = 1.8 Hz, 1H), 7.26 (m, 1H), 7.18 (m, 1H), 7.09 (ddd, *J* = 0.9, 7.1, 8.0 Hz, 1H), 6.49 (t, *J* = 2.2 Hz, 1H), 6.40 (t, *J* = 2.2 Hz, 1H), 6.13 (t, *J* = 2.3 Hz, 1H), 5.40 (dd, *J* = 10.9, 5.8 Hz, 1H), 4.33 (ddd, *J* = 6.3, 8.4, 14.0 Hz, 1H), 4.19 (m, 1H), 4.10 (ddd, *J* = 7.2, 8.2, 13.9 Hz, 1H), 3.34 (m, 3H), 3.26 (m, 1H), 3.12 (s, 3H), 2.30 (dd, *J* = 5.3, 8.1 Hz, 1H), 1.20 (d, J_{PH} = 9.2 Hz, 9H).
^{13}C NMR (CD$_3$CN, δ, 25 °C): 182.3, 149.0, 145.2, 142.4, 139.2, 139.0, 139.0, 130.5, 129.9, 124.6, 122.9, 120.2, 119.6, 112.6, 111.4, 108.8, 108.0, 107.7, 66.4, 60.4 (d, J_{PC} = 15.4 Hz), 53.6, 51.4, 42.7, 38.7, 27.4, 12.7 (d, J_{PC} = 31.6 Hz, 3C).
^{31}P NMR (CD$_3$CN, δ, 25 °C): -8.33 (J_{WP} = 276 Hz).
HRMS (ESI) Calcd for $C_{28}H_{39}BN_{10}O_3PSW^+$ ($[M]^+$) *m/z*: 821.2262, Found: 821.2270

WTp(NO)(PMe$_3$)(η^2-(*N*-propylfuranyl)-(*anti*-6-methanesulfonyl)-5,6-dihydropyridinium) (OTf) (6u)

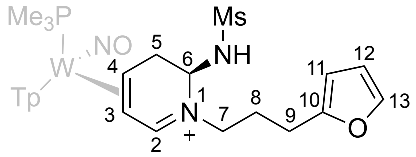

Prepared according to the General Procedure (42 mg, 67% yield)

^{1}H NMR (CD$_3$CN, δ, 25 °C): 8.91 (d, *J* = 5.2 Hz, 1H), 8.12 (d, *J* = 1.7 Hz, 1H), 7.96-7.98 (m, 2H), 7.93 (d, *J* = 1.9 Hz, 1H), 7.78 (d, *J* = 2.1 Hz, 1H), 7.74 (d, *J* = 1.9 Hz, 1H), 7.38 (d, *J* = 1.1 Hz, 1H), 6.47 (t, *J* = 2.3 Hz, 1H), 6.42 (t, *J* = 2.4 Hz, 1H), 6.35 (t, *J* = 2.2 Hz, 1H), 6.32 (dd, *J* = 2.0, 2.9 Hz, 1H), 6.09 (d, *J* = 2.9 Hz, 1H), 5.22-5.28 (b, 1H), 4.21 (ddd, *J* = 3.9, 11.1, 15.1 Hz, 1H), 4.03 (ddd, *J* = 5.2, 10.6, 13.9 Hz, 1H), 3.75 (ddd, *J* = 6.1, 10.4, 13.9 Hz, 1H), 3.28-3.35 (m, 2H), 3.05 (s, 3H), 2.73-277 (m, 2H), 2.43 (dd, *J* = 5.5, 7.8 Hz, 1H), 2.22-2.28 (m, 1H), 2.09-2.13 (m, 1H), 1.18 (d, J_{PC} = 9.3 Hz, 9H).
^{13}C NMR (CD$_3$CN, δ, 25 °C): 182.2, 155.6, 149.0, 145.2, 142.4, 142.3, 139.3, 138.9, 137.7, 111.3, 108.7, 107.9, 106.4, 66.2, 59.8 (d, J_{PC} = 15.5 Hz), 53.2, 51.7, 42.6, 38.4, 303, 25.6, 12.6 (d, J_{PC} = 31.4 Hz, 3C).
HRMS (ESI) Calcd for $C_{25}H_{38}BN_9O_4PSW^+$ ($[M]^+$) *m/z*: 786.2102, Found: 786.2095

WTp(NO)(PMe$_3$)(η^2-(*N*-propylindolyl)-(*anti*-6-methanesulfonyl)-5,6-dihydropyridinium) (OTf) (6v)

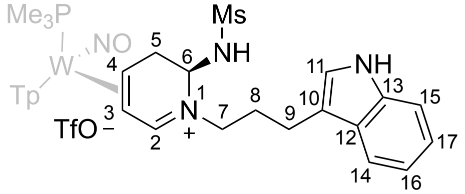

Prepared according to the General Procedure (50 mg, 81% yield).

^{1}H NMR (CD_3CN, δ, 25 °C): 9.11 (br, 1H), 8.89 (d, *J* = 5.2 Hz, 1H), 8.10 (d, *J* = 1.9 Hz, 1H), 7.96 (m, 2H), 7.87 (d, *J* = 2.0 Hz, 1H), 7.76 (d, *J* = 2.3 Hz, 1H), 7.71 (d, *J* = 1.9 Hz, 1H), 7.58 (d, *J* = 8.1 Hz, 1H) 7.37 (d, *J* = 8.2 Hz, 1H), 7.11 (td, *J* = 1.0, 7.6 Hz, 1H), 7.08 (d, *J* = 2.0 Hz, 1H), 7.02 (td, *J* = 0.7, 7.3 Hz, 1H), 6.46 (t, *J* = 2.2 Hz, 1H), 6.41 (t, *J* = 2.2, 1H), 6.32 (t, *J* = 2.2 Hz, 1H), 5.24 (dd, *J* = 6.4, 10.3 Hz, 1H), 4.16 (m, 1H), 4.06 (ddd, *J* = 5.1, 10.8, 13.6 Hz, 1H), 3.77 (ddd, *J* = 5.8, 10.9, 13.7 Hz, 1H), 3.29 (m, 2H), 2.92 (s, 3H), 2.85 (m, 2H), 2.38 (dd, *J* = 5.6, 7.7 Hz, 1H), 2.36 (m, 1H), 1.17 (d, J_{PH} = 9.3 Hz, 9H).
^{13}C NMR (CD_3CN, δ, 25 °C): 182.0, 149.0, 145.2, 142.4, 138.9, 137.7, 137.6, 129.2, 128.2, 123.2, 122.4, 119.7, 119.4, 115.0, 112.3, 108.7, 107.9 (2C), 66.1, 59.7 (d, J_{PC} = 15.5 Hz), 53.7, 51.6, 42.3, 38.6, 32.8, 23.0, 12.7 (J_{PC} = 31.5 Hz, 3C).
HRMS (ESI) Calcd for $C_{29}H_{41}BN_{10}O_3PSW^+$ ($[M]^+$) *m/z*: 835.2418, Found: 835.2407

Synthesis and Characterization of [W] Multicyclic Complexes (7a-7g)

WTp(NO)(PMe$_3$)(η^2-(5,6,8,9-tetrahydroimidazo[1,6]pyridin-2-ium) (OTf) (7a)

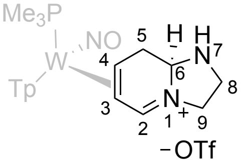

To an oven-dried 4-dram vial was added a stir pea, **5D** (202 mg, 0.23 mmol), ethylenediamine (58 mg, 0.97 mmol), EtCN (2 mL), and DCM (3 mL). The solution was stirred for 5 minutes and then added to a 50 mL of stirring Et_2O. A tan powder was precipitated, collected on a 15 mL F frit, washed 2x with 10 mL of Et_2O, and dried in a desiccator (149 mg, 84% yield).

^{1}H NMR (CD_3CN, δ, 25 °C): 9.04 (d, J = 5.6 Hz, 1H), 8.08 (d, J = 2.0 Hz, 1H), 7.99 (d, J = 2.0 Hz, 1H), 7.96 (d, J = 2.3 Hz, 1H), 7.75-7.77 (m, 2H), 6.46 (t, J = 2.3 Hz, 1H), 6.40 (t, J = 2.3 Hz, 1H), 6.33 (t, J = 2.3 Hz, 1H), 4.39 (dd, J = 5.8, 11.2 Hz, 1H), 4.19 (dd, J = 8.2, 12.4 Hz, 1H), 3.80-3.84 (m, 1H), 3.69-3.74 (m, 1H), 3.36-3.41 (m, 2H), 3.28-3.32 (m, 1H), 3.05 (dt, J = 8.8, 12.4 Hz, 1H), 2.33 (dd, J = 5.6, 9.2 Hz, 1H), 1.19 (d, J_{PH} = 9.2 Hz, 9H).
^{13}C NMR (CD_3CN, δ, 25 °C): 177.2, 148.9, 145.4, 142.5, 139.2, 138.7, 137.4, 122.1 (q, J_{FC} = 321.0 Hz), 108.6, 107.8 (2C), 75.5, 61.9 (d, J_{PC} = 15.4 Hz), 51.5, 50.0, 44.8, 34.5, 12.82 (d, J_{PC} = 30.9 Hz, 3C).
^{31}P NMR (CD_3CN, δ, 25 °C): -8.87 (J_{WP} = 278 Hz).
HRMS (ESI) Calcd for $C_{19}H_{30}BN_9OPW^+$ 626.1908; Found: 626.1928

SCXRD Data on page **S59**

WTp(NO)(PMe$_3$)(η^2-(5,6,7,8,9,10-hexahydropyrimido[1,6]pyridin-2-ium) (OTf) (7b)

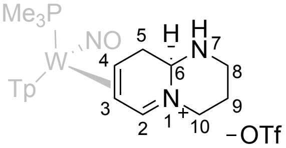

To an oven-dried 4-dram vial was added a stir pea, **5D** (217 mg, 0.24 mmol), diaminopropane (75 mg, 1.01 mmol), EtCN (2 mL), and DCM (3 mL). The solution was stirred for 10 minutes and then added to a 50 mL of stirring Et_2O. A tan powder was precipitated, collected on a 15 mL F frit, washed 2x with 10 mL of Et_2O, and dried in a desiccator (155 mg, 80% yield).

^{1}H NMR (CD_3CN, δ, 25 °C): 8.61 (d, J = 4.8 Hz, 1H), 8.05 (d, J = 2.1 Hz, 1H), 8.00 (d, J = 2.1 Hz, 1H), 7.94-7.95 (m, 2H), 7.78 (d, J = 2.4 Hz, 1H), 7.72 (d, J = 2.2 Hz, 1H), 6.45 (t, J = 2.3 Hz, 1H), 6.40 (t, J = 2.3 Hz, 1H), 6.36 (t, J = 2.3 Hz, 1H), 4.67 (dd, J = 6.5, 10.8 Hz, 1H), 4.48 (td, J = 3.6, 13.3 Hz, 1H), 4.07-4.12 (m, 1H), 4.03-4.06 (m, 1H), 3.23-3.27 (m, 2H), 3.12 (dq, J = 2.1, 14.1 Hz, 1H), 2.85 (td, J = 2.8, 11.9 Hz, 1H), 2.26 (dd, J = 4.8, 8.5 Hz, 1H), 1.76-1.83 (m, 1H), 1.71 (d, J = 13.3 Hz, 1H), 1.18 (d, J_{PH} = 9.2 Hz, 9H).
^{13}C NMR (CD_3CN, δ, 25 °C): 178.4, 148.8, 145.3, 142.5, 139.2, 138.8, 137.7, 122.1 (q, J_{FC} = 321.5 Hz), 108.6, 107.9, 107.8, 71.0, 59.0 (d, J_{PC} = 15.0 Hz), 53.8, 50.9, 44.4, 37.0, 27.8, 12.9 (d, J_{PC} = 31.0 Hz, 3C).
HRMS (ESI) Calcd for $C_{20}H_{32}BN_9OPW^+$ 640.2064; Found: 640.2072
SCXRD Data on **S60**

WTp(NO)(PMe$_3$)(η^2-5,6,8,9,10,11-hexahydro[1,7]diazepino[1,6]-pyridin-2-ium) (OTf) (7c)

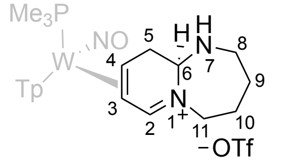

To an oven-dried 4-dram vial was added a stir pea, **5D** (251 mg, 0.28 mmol), diaminobutane (112 mg, 1.27 mmol), EtCN (2 mL), and DCM (3 mL). The solution was stirred for 5 minutes and then added to a 50 mL of stirring Et$_2$O. A tan powder was precipitated, collected on a 15 mL F frit, washed 2x with 10 mL of Et$_2$O, and dried in a desiccator (189 mg, 83% yield).

^{1}H NMR (CD$_3$CN, δ, 25 °C): 8.90 (d, J = 5.2 Hz, 1H), 8.10 (d, J = 2.0 Hz, 1H), 7.96 (d, J = 2.0 Hz, 1H), 7.94-7.95 (m, 2H), 7.77 (d, J = 2.4 Hz, 1H), 7.72 (d, J = 2.2 Hz, 1H), 6.45 (t, J = 2.3 Hz, 1H), 6.39 (t, J = 2.3 Hz, 1H), 6.33 (d, J = 2.3 Hz, 1H), 4.69-4.72 (m, 1H), 4.02-4.07 (m, 2H), 3.97 (dd, J = 7.3, 13.7 Hz, 1H), 3.16-3.20 (m, 2H), 3.00-3.06 (m, 2H), 2.82-2.86 (m, 1H), 2.45 (br s, 1H), 2.22 (dd, J = 5.4, 8.0 Hz, 1H), 1.86-1.91 (m, 1H), 1.78-1.84 (m, 1H), 1.62-1.68 (m, 1H), 1.57-1.62 (m, 1H), 1.20 (d, J_{PH} = 9.2 Hz, 9H).
^{13}C NMR (CD$_3$CN, δ, 25 °C): 180.0, 148.8, 145.4, 142.5, 139.2, 138.7, 137.5, 108.5, 107.8, 74.5, 60.9 (d, J_{PC} = 15.3 Hz), 56.7, 49.6, 47.0, 37.4, 32.3, 29.7, 12.9 (d, J_{PC} = 25.1 Hz, 3C).
HRMS (ESI) Calcd for $C_{21}H_{34}BN_9OPW^+$ ([M]$^+$) *m/z*: 654.2221, Found: 654.2227

WTp(NO)(PMe$_3$)(η^2-(*N*-aminoethyl)-5,6,8,9-tetrahydroimidazo[1,6]pyridin-2-ium) (OTf) (7d)

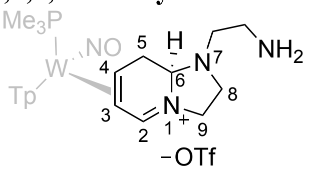

To an oven-dried 4-dram vial was added a stir pea, 5D (83 mg, 0.10 mmol), diethylene triamine (0.05 mL, 0.46 mmol), DCM (2 mL), and EtCN (2 mL). This solution was stirred for 30 minutes and then added to 100 mL of stirring Et$_2$O. A light brown powder was precipitated, collected on a 15 mL F frit, washed 3x with 15 mL of Et$_2$O, and dried in a desiccator overnight (61 mg, 88% yield).

^{1}H NMR (CD$_3$CN, δ, 25 °C): 8.95 (d, J = 5.4 Hz, 1H), 8.09 (d, J = 2.0 Hz, 1H), 7.97 (d, J = 2.0 Hz, 1H), 7.96 (d, J = 2.3 Hz, 1H), 7.96 (d, J = 2.4 Hz, 1H), 7.77 (d, J = 2.4 Hz, 1H), 7.76 (d, J = 2.4 Hz, 1H), 6.46 (t, J = 2.3 Hz, 1H), 6.41 (t, J = 2.3 Hz, 1H), 6.33 (t, J = 2.3 Hz, 1H), 4.31 (dd, J = 8.5, 12.5 Hz, 1H), 3.97(2H, m), 3.66(m, 1H), 3.44 (m, 1H), 3.29-3.36 (m, 2H), 2.96 (m, 1H), 2.83 (m, 3H), 2.42 (dt, J = 5.1, 12.0 Hz, 1H), 2.37 (dd, J = 6.0, 8.0 Hz, 1H), 1.19 (d, J_{PH} = 9.2 Hz, 9H).
^{13}C NMR (CD$_3$CN, δ, 25 °C): 178.5, 149.0, 145.4, 142.5, 139.2, 138.8, 137.5, 108.6, 107.8, 107.8, 77.5, 61.9 (d, J_{PC} = 15.6), 55.9, 50.6, 50.3, 50.1, 40.8, 34.7, 12.8 (d, J_{PC} = 30.0 Hz, 3C).
HRMS (ESI) Calcd for $C_{21}H_{35}BN_{10}OPW^+$ 669.2330; Found: 669.2388

WTp(NO)(PMe$_3$)(η^2-(*N*-hydroxyethyl)-5,6,8,9-tetrahydroimidazo[1,6]pyridin-2-ium) (OTf) (7e)

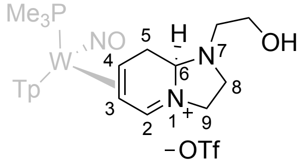

To an oven-dried 4-dram vial was added a stir pea, **4D** (100 mg, 0.1234 mmol), EtCN (2 mL), and DCM (2 mL). 2-(2-aminoethylamino)ethanol (51.41 mg, 0.4936 mmol) was then added dropwise to the reaction mixture. The solution was stirred at 25°C for 30 minutes and then added to 150 mL of stirring Et_2O. A tan powder was precipitated, collected on a 15 mL F frit, washed 2x with 10 mL of Et_2O, and dried in a desiccator (90 mg, 89% yield).

^{1}H NMR (CD$_3$CN, δ, 25 °C): 8.96 (d, *J* = 5.6 Hz, 1H), 8.09 (d, *J* = 2.0 Hz, 1H), 7.98 (d, *J* = 2.0 Hz, 1H), 7.96 (d, *J* = 2.3 Hz, 1H), 7.956 (d, *J* = 2.4 Hz, 1H), 7.77 (d, *J* = 2.1 Hz, 1H), 7.76 (d, *J* = 2.4 Hz, 1H), 6.46 (t, *J* = 2.3 Hz, 1H), 6.41 (t, *J* = 2.3 Hz, 1H), 6.33 (t, *J* = 2.3 Hz, 1H), 4.31 (dd, *J* = 8.2, 12.4 Hz, 1H), 4.03 (dd, *J* = 5.7, 11.0 Hz, 1H), 3.97 (m, 1H), 3.72-3.63 (m, 3H), 3.46 (m, 1H), 3.36-3.31 (m, 2H), 3.06 (m, 1H), 2.57 (m, 1H), 2.54 (m, 1H), 2.38 (dd, *J* = 5.9, 8.2 Hz, 1H), 1.20 (d, J_{PH} = 9.2 Hz, 9H).
^{13}C NMR (CD$_3$CN, δ, 25 °C): 178.5, 149.0, 145.4 (d, *J* = 2.3 Hz), 142.5, 139.2, 138.8, 137.4, 108.6, 107.81, 107.79, 77.3, 61.9 (d, J_{PC} = 15.8 Hz), 60.4, 54.9, 50.6, 50.5, 50.1, 34.5 (d, J_{PC} = 2.7 Hz), 12.8 (d, J_{PC} = 31.7 Hz, 3C).
HRMS (ESI) Calcd for $C_{21}H_{34}BN_9O_2PW^+$ 670.2170; Found: 670.2178
SCXRD Data on **S61**

WTp(NO)(PMe$_3$)(η^2-(5,6,8,9-tetrahydrothiazolo[1,6]pyridin-2-ium) (OTf) (7f)

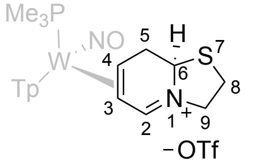

To an oven-dried 4-dram vial was added a stir pea, **4D** (100 mg, 0.1234 mmol), basic alumina, EtCN (2 mL), and DCM (2 mL). To a separate vial was added cysteamine (38.03 mg, 0.493 mmol), EtCN (1 mL), DCM (1 mL), and MeOH (1 mL). The cysteamine solution was then added dropwise to **4D**. The combined reaction mixture was stirred at -20°C for 18 hr, after which it was filtered through a 15 mL M frit charged with celite. MeOH (15 mL) was used to wash the product off the celite into a filter flask. The solution of product was then evaporated in vacuo to leave a brown film. This film was redissolved in EtCN (1 mL) and DCM (1 mL), and then added to 150 mL of stirring Et_2O. A tan powder was precipitated, collected on a 15 mL F frit, washed 2x with 10 mL of Et_2O, and dried in a desiccator (70 mg, 72% yield).

^{1}H NMR (CD$_3$CN, δ, 25 °C): 9.03 (d, *J* = 5.4 Hz, 1H), 8.08 (d, *J* = 1.9 Hz, 1H), 8.00 (d, *J* = 1.9 Hz, 1H), 7.97 (d, *J* = 2.3 Hz, 1H), 7.96 (d, *J* = 2.4 Hz, 1H), 7.78 (d, *J* = 2.0 Hz, 1H), 7.77 (d, *J* = 2.3 Hz, 1H), 6.47 (t, *J* = 2.3 Hz, 1H), 6.42 (t, *J* = 2.3 Hz, 1H), 6.34 (t, *J* = 2.3 Hz, 1H), 5.01 (dd, *J* = 6.2, 11.9 Hz, 1H), 4.48 (m, 1H), 4.33 (p, *J* = 6.1 Hz, 1H), 4.00 (m, 1H), 3.48 (m, 1H), 3.42 (m, 1H), 3.19 (m, 1H), 3.11 (m, 1H), 2.47 (dd, *J* = 6.0, 8.3 Hz, 1H), 1.17 (d, J_{PH} = 9.34 Hz, 9H).
^{13}C NMR (CD$_3$CN, δ, 25 °C): 178.7, 149.1, 145.3 (d, *J* = 2.6 Hz), 142.5, 139.3, 138.9, 137.5, 108.7, 107.9, 107.8, 64.0 (d, J_{PC} = 16.4 Hz), 61.3, 57.5, 50.7, 36.4 (d, J_{PC} = 1.8 Hz), 28.6, 12.7 (d, J_{PC} = 31.8 Hz, 3C).
HRMS (ESI) Calcd for $C_{19}H_{29}BN_8OPSW^+$ 643.1520; Found: 643.1526

WTp(NO)(PMe$_3$)(η^2-(5,6,9,10-tetrahydroindolo[7,8]quinolin-2-ium) (OTf) (7g)

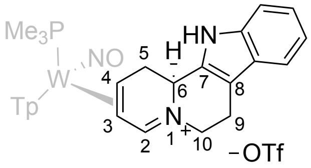

To an oven-dried test tube, **6t** (120 mg, 0.124 mmol) was added and dissolved in 2 mL of MeCN. In a separate oven-dried test tube, HOTf (0.04 mL, 0.452 mmol) was added and diluted with 2 mL of MeCN. These solutions were both cooled to -40 °C for 5 minutes, and then HOTf was added dropwise to the stirring **6s**/MeCN solution. This reaction was allowed to progress for 5 days at -40 °C. Reaction was then diluted with 30 mL of sat. aq. $NaHCO_3$ (30 mL) and DCM (30 mL). Organic layer was rinsed with sat. aq. $NaHCO_3$ (30 mL) two more times and the aqueous layer was back extracted with DCM (30 mL) three times. Combined organic fractions were dried with Na_2SO_4, which was removed via gravity filtration. Dried organic layer was concentrated under high vacuum to an oil and then precipitated in Et_2O (100 mL). A pale brown solid was isolated on a 15 mL F frit, washed with 10 mL of Et_2O, and dried. (84 mg, 77% Yield)

^{1}H NMR (CD$_3$CN, δ, 25 °C): 9.59 (br s, 1H), 9.00 (d, J = 5.3 Hz, 1H), 8.12 (d, J = 2.0 Hz, 1H), 8.06 (d, J = 2.0 Hz, 1H), 7.98 (d, J = 2.4 Hz, 2H), 7.80 (d, J = 2.3 Hz, 1H), 7.78 (d, J = 2.1 Hz, 1H), 7.50 (d, J = 7.9 Hz, 1H), 7.42 (d, J = 8.1, 1H), 7.18 (td, J = 0.9, 7.5 Hz, 1H), 7.10 (t, J = 7.9 Hz, 1H), 6.48 (t, J = 2.2 Hz, 1H), 6.42 (t, J = 2.2 Hz, 1H), 6.39 (t, J = 2.2 Hz, 1H), 5.27 (dd, J = 6.6, 12.4 Hz, 1H), 4.42 (td, J = 4.4, 12.6 Hz, 1H), 4.36 (dd, J = 5.4, 12.6 Hz, 1H), 4.08 (dddd, J = 1.5, 4.0, 12.0, 14.7 Hz, 1H), 3.61 (dd, J = 6.9, 14.2 Hz, 1H), 3.50 (m, 1H), 3.03 (dddd, J = 2.5, 5.2, 12.0, 15.3 Hz, 1H), 2.93 (dd, J = 4.3, 15.2 Hz, 1H), 2.41 (dd, J = 5.3, 8.0 Hz, 1H), 1.28 (d, J_{PH} = 9.2 Hz, 9H).
^{13}C NMR (CD$_3$CN, δ, 25 °C): 180.8, 148.9, 145.3, 142.6, 139.3, 138.9, 137.8, 137.7, 131.7, 127.2, 123.0, 120.5, 119.1, 112.2, 108.7, 108.0, 107.9, 107.9, 62.9 (d, J_{PC} = 15.7 Hz), 54.9, 54.7, 50.5, 35.9, 22.3, 13.0 (d, J_{PC} = 29.9 Hz, 3C).
HRMS (ESI) Calcd for $C_{27}H_{34}BN_9OPW^+$ 726.2221; Found: 726.2196

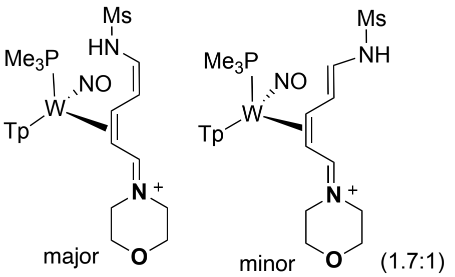

Synthesis and characterization of WTp(NO)(PMe$_3$)(η^2-(*N*-mesyl)-pyridinium) (OTf) (8)
To an oven-dried 10 mL test tube was added, **4D** (56 mg, 0.06 mmol) and DCM (2 mL). To a separate dried 10 mL tube was added morpholine (45 mg, 0.52 mmol) and DCM. Both solutions were chilled at -20 °C for 15 minutes, and then the amine solution was added to the solution of **4D**. The solution was then manually swirled, sat in the bath for 15 minutes, and then added to a 50 mL of stirring Et$_2$O. A tan powder was precipitated, collected on a 15 mL F frit, washed 2x with 10 mL of Et$_2$O, and dried in a desiccator (45 mg, 79% yield).
^{1}H NMR (CD$_3$CN, δ, 25 °C): *Major*: 8.04-8.06 (m, 1H, Tp3/5), 7.95 – 7.98 (m, 2H, Tp3/5), 7.92 (d, *J* = 2.4 Hz, 1H, Tp3/5), 7.88 (d, *J* = 2.4 Hz, 1H, Tp3/5), 7.66 (d, *J* = 11.8 Hz, 1H), 7.49 (d, *J* = 1.9 Hz, 1H, Tp3/5), 7.28-7.31 (m, 2H), 6.91-6.95 (m, 2H), 6.41-6.46 (m, 3H, Tp4), 6.12 (d, *J* = 8.6 Hz, 1H), 5.67 (dd, *J* = 8.6, 10.7 Hz, 1H), 3.99-4.03 (m, 1H), 3.89-3.97 (m, 2H), 3.68-3.75 (m, 1H), 3.58-3.62 (m, 1H), 3.46-3.52 (m, 1H), 3.25 (d, *J* = 13.5 Hz, 1H), 3.15 (t, *J* = 5.1 Hz, 1H), 3.09 (s, 3H), 2.71 (ddd, *J* = 3.9, 9.6, 23.2 Hz, 1H), 1.17 (d, J_{PH} = 9.4 Hz, 9H, PMe$_3$). *Minor*: 8.04-8.06 (m, 1H, Tp3/5), 7.95 – 7.98 (m, 1H, Tp3/5), 7.91 (d, *J* = 2.4 Hz, 1H, Tp3/5), 7.87 (d, *J* = 2.4 Hz, 1H, Tp3/5), 7.73 (d, *J* = 11.9 Hz, 1H, Tp3/5), 7.48 (d, *J* = 1.9 Hz, 1H, Tp3/5), 7.28-7.31 (m, 2H), 6.91-6.95 (m, 2H), 6.41-6.46 (m, 3H, Tp4), 6.27 (d, *J* = 13.8 Hz, 1H), 6.16 (dd, *J* = 10.8, 13.8 Hz, 1H), 4.05-4.10 (m, 1H), 3.99-4.03 (m, 1H), 3.89-3.97 (m, 1H), 3.82-3.85 (m, 1H), 3.68-3.75 (m, 1H), 3.58-3.62 (m, 1H), 3.46-3.52 (m, 1H), 3.20 (d, *J* = 13.5 Hz, 1H), 3.02 (s, 3H), 2.66 (ddd, *J* = 3.9, 9.8, 23.2 Hz, 1H), 1.20 (d, J_{PH} = 9.4 Hz, 9H, PMe$_3$).
^{13}C NMR (CD$_3$CN, δ, 25 °C): (*Major & Minor combined due to substantial overlap*) 173.0, 173.0, 144.6, 144.4, 144.3, 142.1, 141.7, 138.0, 138.0, 137.9, 137.9, 129.5, 125.2, 123.7, 120.5, 115.9, 113.8, 110.7, 107.5, 107.4, 107.2, 107.1, 106.8, 106.8, 68.4, 66.6, 66.1, 65.3, 64.2, 63.4, 57.7, 57.5, 55.9, 55.8, 54.7, 49.4, 47.1, 47.0, 43.6, 42.6, 40.9, 40.2, 39.0, 11.8, (d, *J* = 31.6 Hz, 3C), 11.1 (d, *J* = 31.6 Hz, 3C).
HRMS (ESI) Calcd for $C_{22}H_{36}BN_9O_4PSW^+$ ([M]$^+$) *m/z*: 748.1945, Found: 748.1952

Synthesis and characterization of WTp(NO)(PMe$_3$)(η^2-(*N*-methyl)-(6-methylamino)-5,6-dihydropyridinium) (OTf) (9)

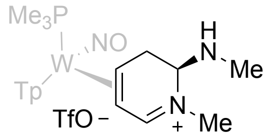

To an oven-dried 4-dram vial was added a stir pea, **5D** (101 mg, 0.11 mmol), 2.0M methylamine in THF (0.2 mL, 0.40 mmol), EtCN (1 mL), and DCM (2 mL). The solution was stirred for 5 minutes and then added to a 50 mL of stirring Et_2O. A tan powder was precipitated, collected on a 15 mL F frit, washed 2x with 10 mL of Et_2O, and dried in a desiccator (77 mg, 87% yield).

^{1}H NMR (CD$_3$CN, δ, 25 °C): 8.82 (d, J = 5.0 Hz, 1H), 8.07 (d, J = 2.0 Hz, 1H), 7.99 (d, J = 2.1 Hz, 1H), 7.95 (d, J = 2.3 Hz, 2H), 7.78 (d, J = 2.4 Hz, 1H), 7.73 (d, J = 2.2 Hz, 1H), 6.48 (t, J = 2.3 Hz, 1H), 6.42 (t, J = 2.3 Hz, 1H), 6.38 (t, J = 2.3 Hz, 1H), 4.54 (dd, J = 6.4, 11.1 Hz, 1H), 4.19-4.24 (m, 1H), 3.61 (s, 3H), 3.25-3.29 (m, 1H), 3.18 (dd, J = 6.4, 14.7 Hz, 1H), 2.48 (s, 3H), 2.29 (dd, J = 5.0, 8.1 Hz, 1H), 1.22 (d, J_{PH} = 9.2 Hz, 9H).

^{13}C NMR (CD$_3$CN, δ, 25 °C): 181.5, 148.8, 145.4, 142.5, 139.2, 138.8, 137.7, 108.6, 107.9, 107.8, 74.0, 59.6, 50.8, 40.3, 35.1, 31.5, 12.9 (d, J_{PC} = 31.3 Hz, 3C).

HRMS (ESI) Calcd for $C_{19}H_{32}BN_9OPW^+$ ([M]$^+$) *m/z*: 628.2064, Found: 628.2064

Synthesis and characterization of WTp(NO)(PMe_3)(η^2-(*N*-propyl)-pyridinium) (OTf) (10)

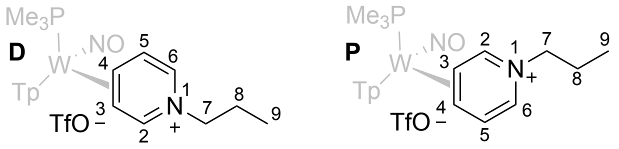

To an oven-dried 10 mL test tube, a stir bar, **6b** (51 mg, 0.059 mg) were added, and 3 mL of DCM were added. To this stirring solution, Et_3N (0.02 mL, 0.143 mmol) was added. Reaction was allowed to stir for one hour at room temperature. This mixture was concentrated to an oil and then precipitated into Et_2O (100 mL). The powder was then collected on a frit, washed with 10 mL of Et_2O, and dried. (38 mg, 83% yield)

^{1}H NMR (CD_3CN, δ, 25 °C): **major**: 8.72 (d, J = 5.3 Hz, 1H), 8.00 (overlaps, m, 1H), 7.99 (overlaps, m, 2H), 7.96 (d, J = 1.7 Hz, 1H), 7.85 (d, J = 2.2 Hz, 1H), 7.44 (d, J = 1.8 Hz, 1H), 6.88 (t, J = 6.2 Hz, 1H), 6.42 (overlaps, m, 1H), 6.38 (overlaps, m, 1H), 6.35 (t, J = 2.2 Hz, 1H), 6.12 (d, J = 7.3 Hz, 1H), 3.97 (ddd, J = 5.7, 9.3, 13.3 Hz, 1H), 3.90 (overlaps, m, 1H), 3.83 (ddd, J = 5.7, 7.7, 12.2 Hz, 1H), 2.35 (overlaps, m, 1H), 1.87 (overlaps, m, 2H), 1.18 (d, J_{PH} = 9.1 Hz, 9H), 1.02 (overlaps, m, 3H)
minor: 8.68 (d, J = 4.7 Hz, 1H), 8.01 (d, J = 1.6 Hz, 1H), 8.00 (overlaps, m, 1H), 7.99 (overlaps, m, 2H), 7.86 (d, J = 2.1 Hz, 1H), 7.36 (d, J = 1.8 Hz, 1H), 7.09 (t, J = 6.7 Hz, 1H), 6.42 (overlaps, m, 1H), 6.38 (overlaps, m, 1H), 6.36 (t, J = 2.2 Hz, 1H), 6.06 (d, J = 7.2 Hz, 1H), 4.03 (ddd, J = 5.7, 9.3, 13.3 Hz, 1H), 3.90 (overlaps, m, 1H), 3.53 (m, 1H), 2.35 (overlaps, m, 1H), 1.87 (overlaps, m, 2H), 1.25 (d, J_{PC} = 8.8 Hz, 9H), 1.02 (overlaps, m, 3H).
^{13}C NMR (CD_3CN, δ, 25 °C): **major**: 172.0, 146.2, 145.3, 142.3, 139.0, 138.6, 137.8, 129.3, 120.4, 108.3, 107.9 (overlaps), 107.7, 65.8 (d, J_{PC} = 11.9 Hz), 60.0, 59.0, 25.2, 12.5 (d, J_{PC} = 30.9 Hz, 3C), 11.1
minor: 167.3, 146.0, 142.2, 142.1, 138.9, 138.4, 137.7, 132.3, 118.3 (overlaps solvent), 108.2, 107.9 (overlaps), 107.3, 62.9, 61.4 (d, J_{PC} = 5.8 Hz), 60.1, 25.3, 13.2 (d, J_{PC} = 30.3 Hz, 3C), 11.0.
HRMS (ESI) Calcd for $C_{21}H_{36}BN_9O_3PW^+$ ($[M]^+$) *m/z*: 625.1961, Found: 625.1961

NMR Spectroscopy:

Figure S1: ^{1}H NMR, 800 MHz, CD_3CN, 25 °C, **6a**

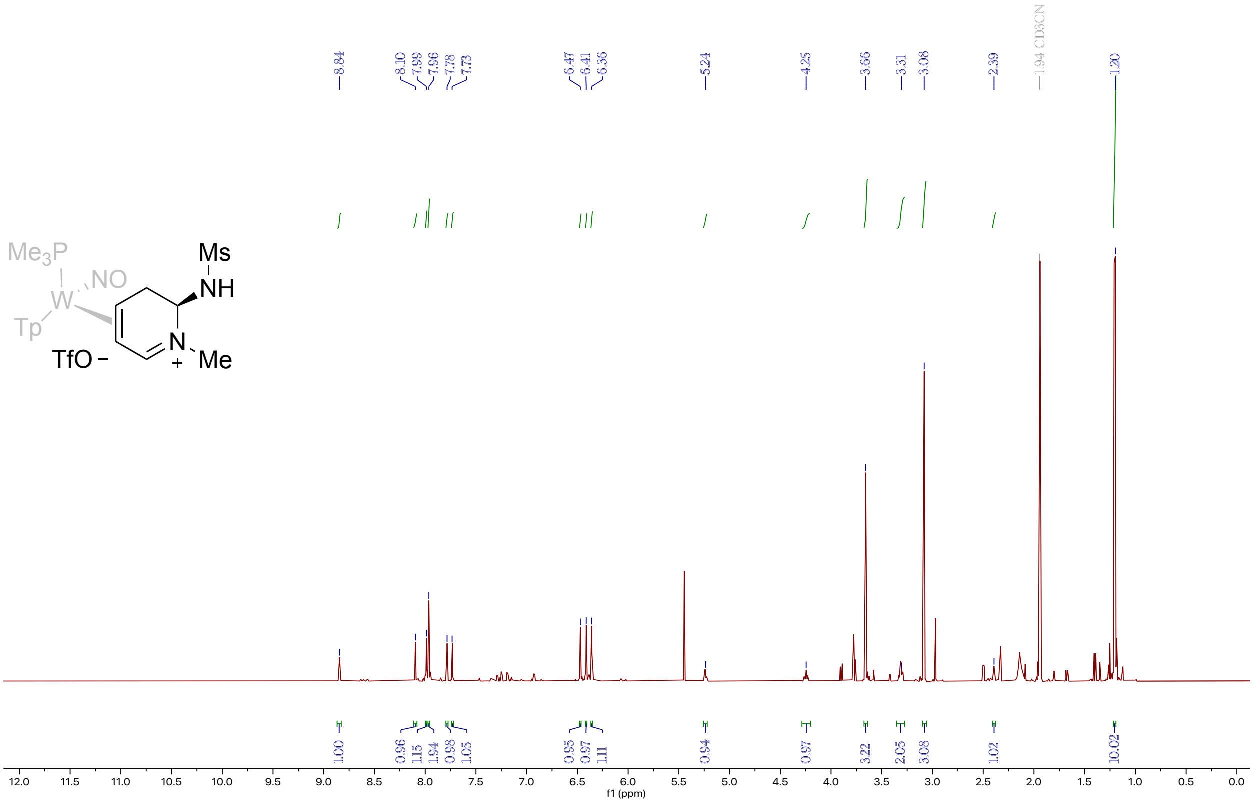

Figure S2: ^{13}C NMR, 200 MHz, CD_3CN, 25 °C, **6a**

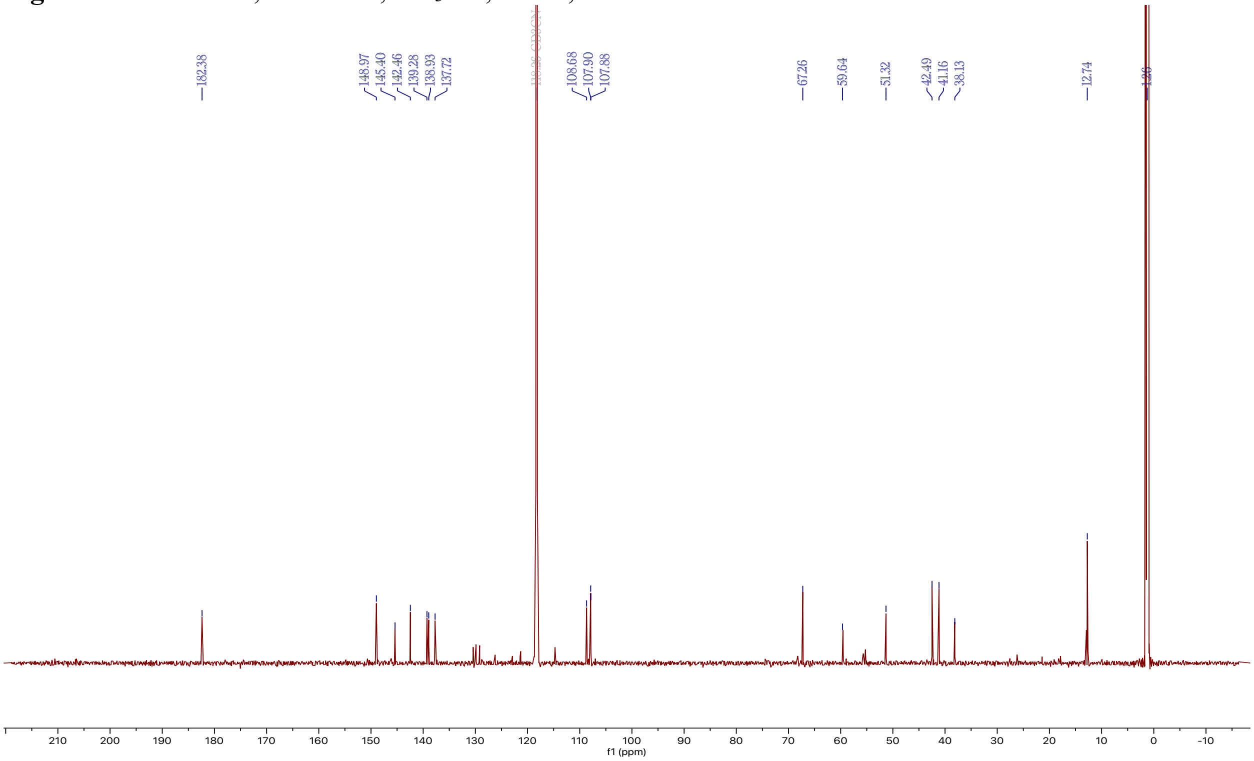

Figure S3: ^{1}H NMR, 800 MHz, CD_2Cl_2, 25 °C, **6b**

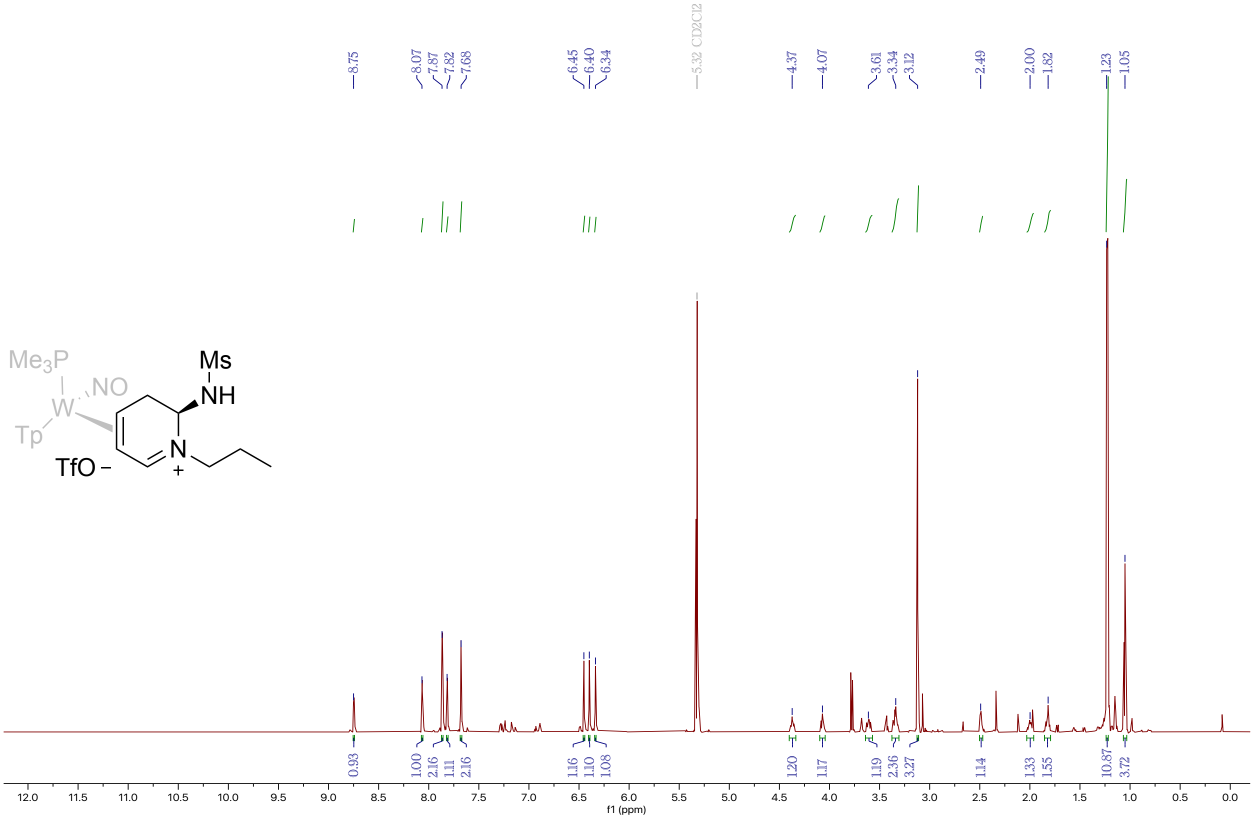

Figure S4: ^{13}C NMR, 200 MHz, CD_2C_2, 25 °C, **6b**

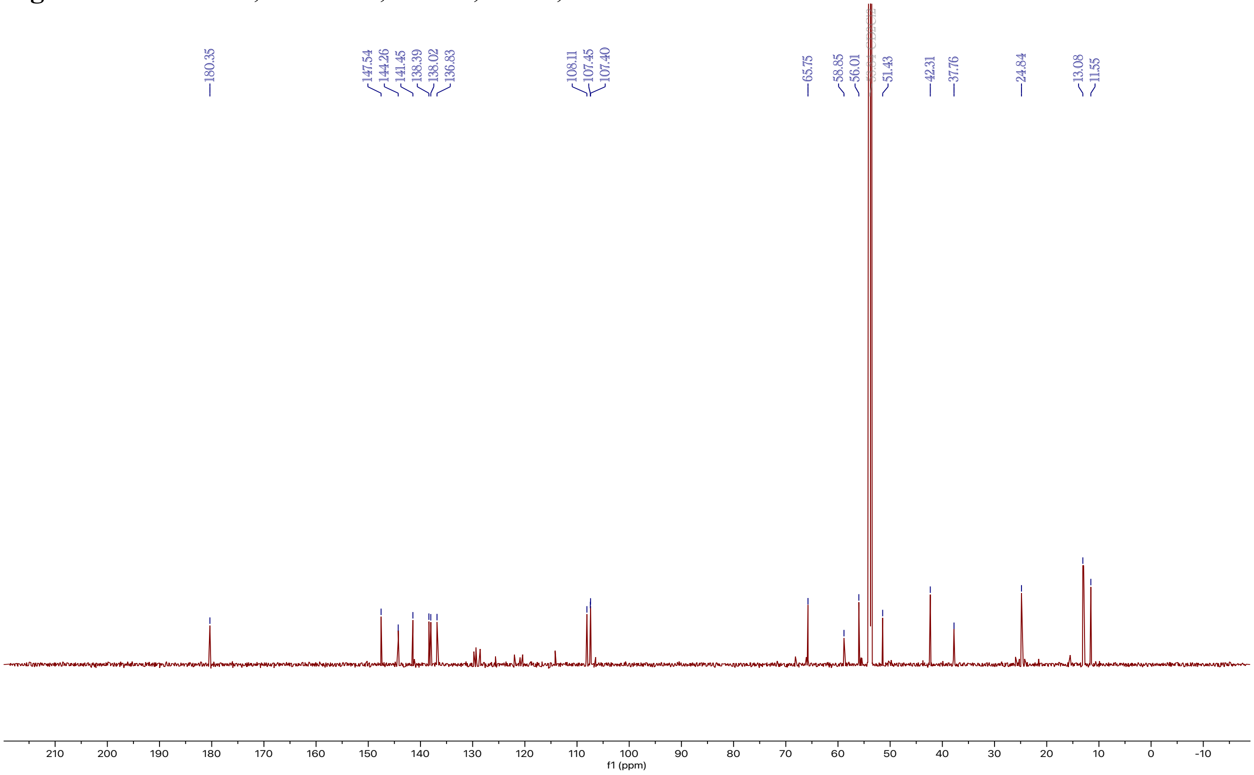

Figure S5: ^{1}H NMR, 800 MHz, CD_3CN, 25 °C, **6c**

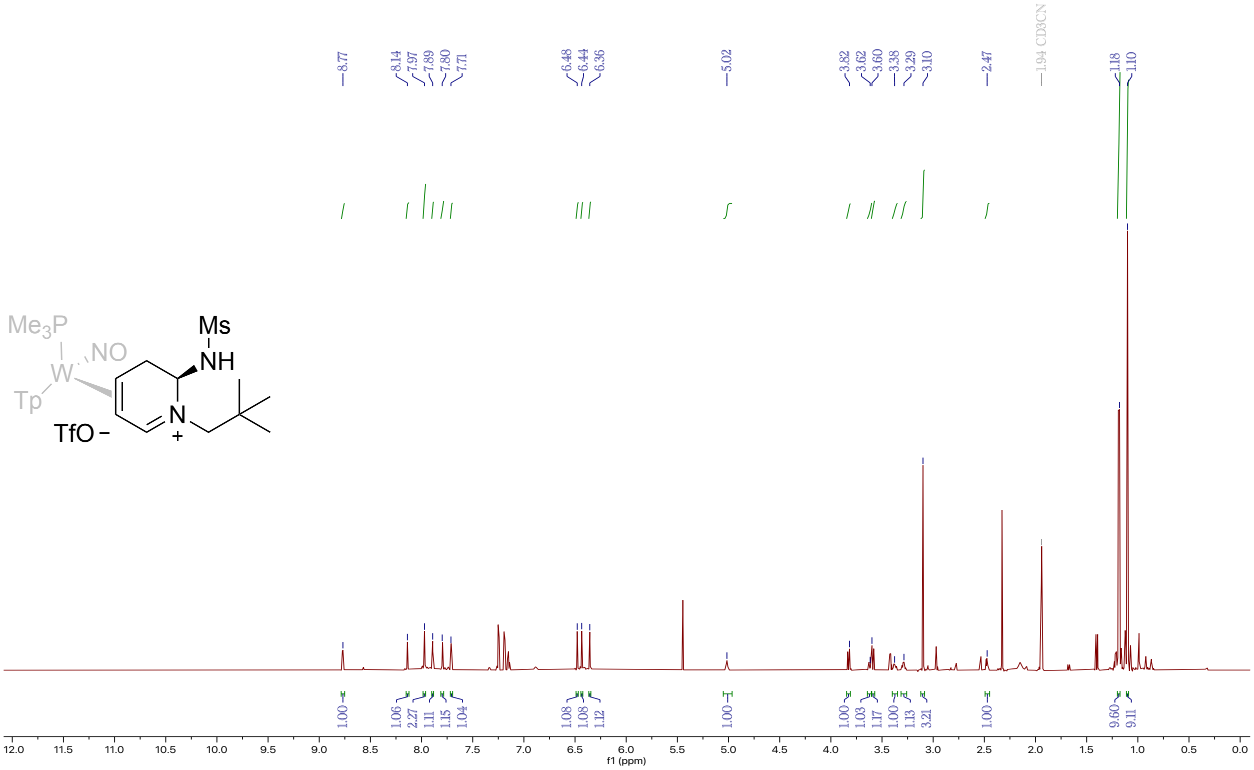

Figure S6: ^{13}C NMR, 200 MHz, CD_3CN, 25 °C, **6c**

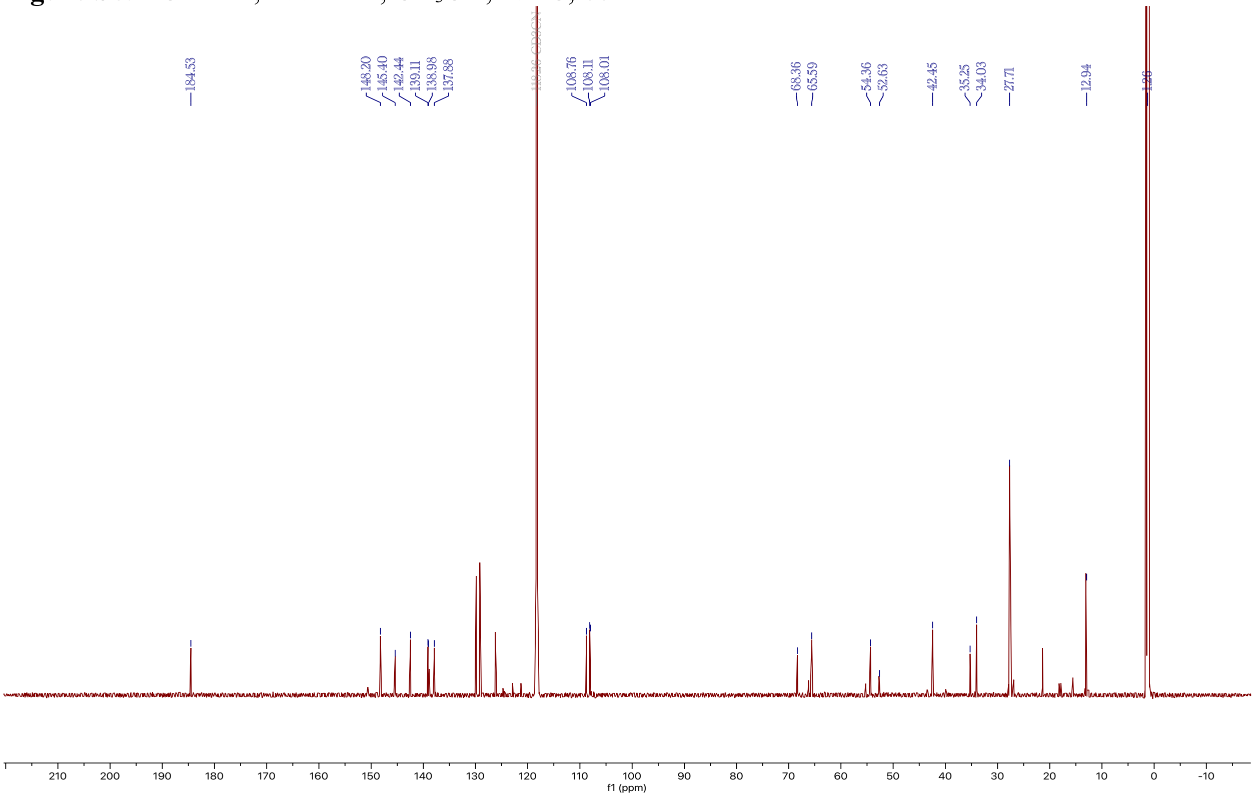

Figure S7: ^{1}H NMR, 800 MHz, CD_2Cl_2, 25 °C, **6d**

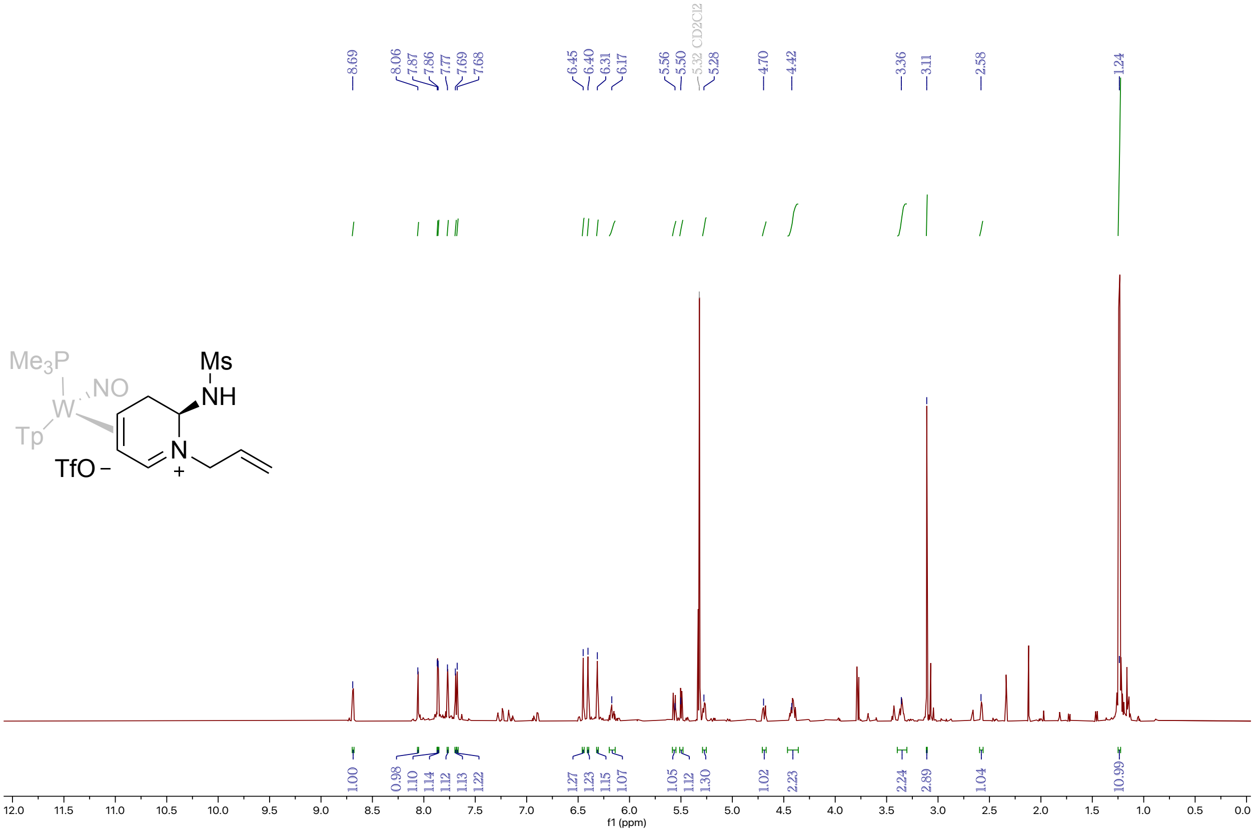

Figure S8: ^{13}C NMR, 200 MHz, CD_2Cl_2, 25 °C, **6d**

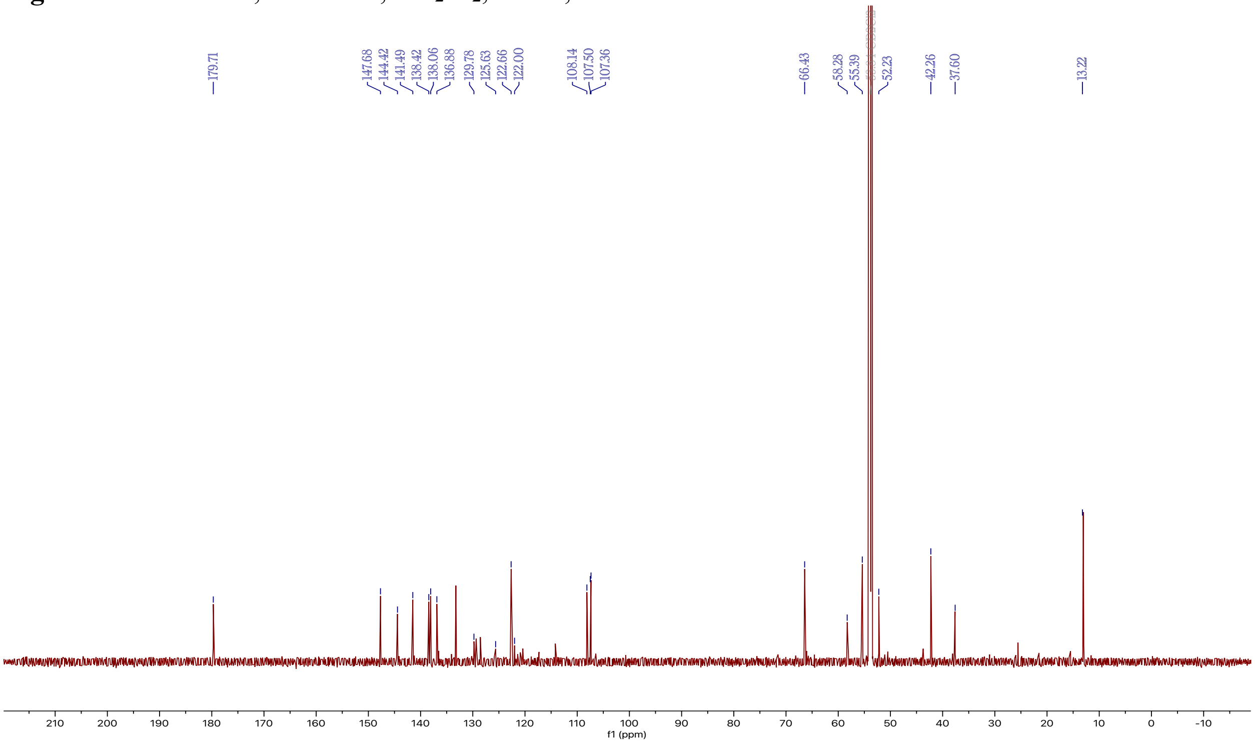

Figure S9: ^{1}H NMR, 800 MHz, CD_3CN, 25 °C, **6e**

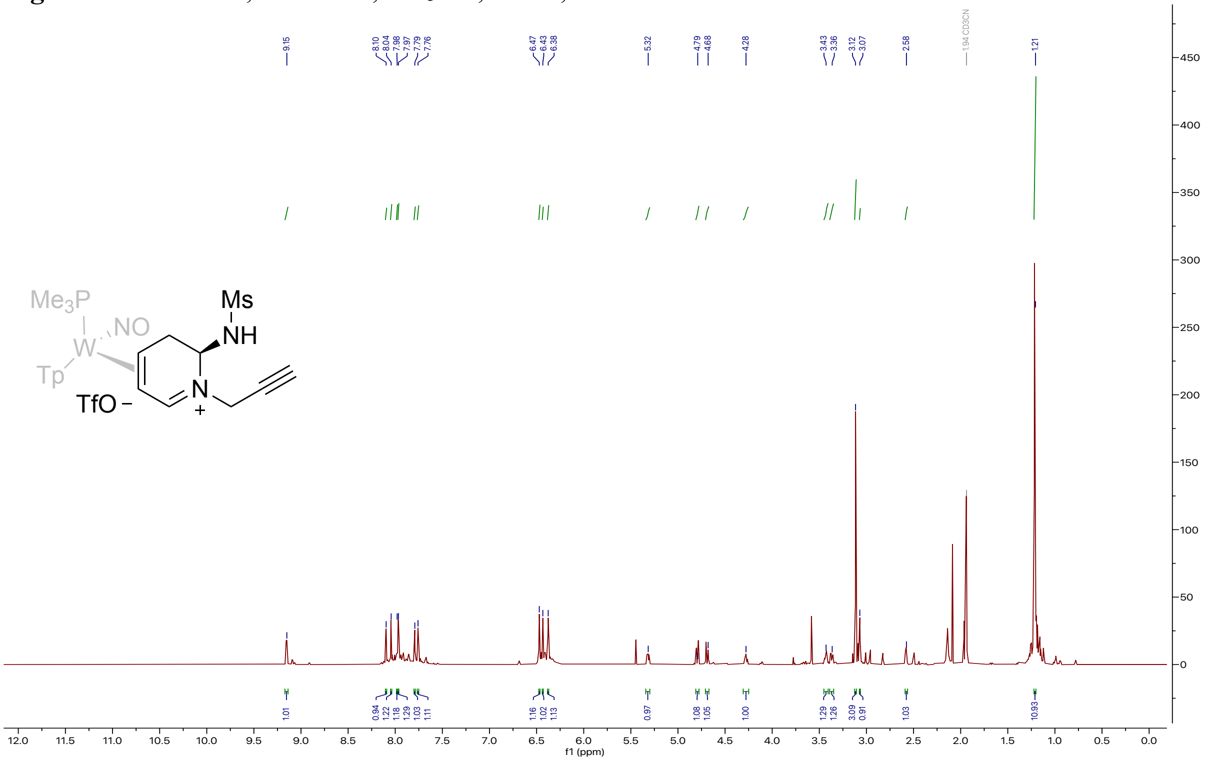

Figure S10: ^{13}C NMR, 200 MHz, CD_3CN, 25 °C, **6e**

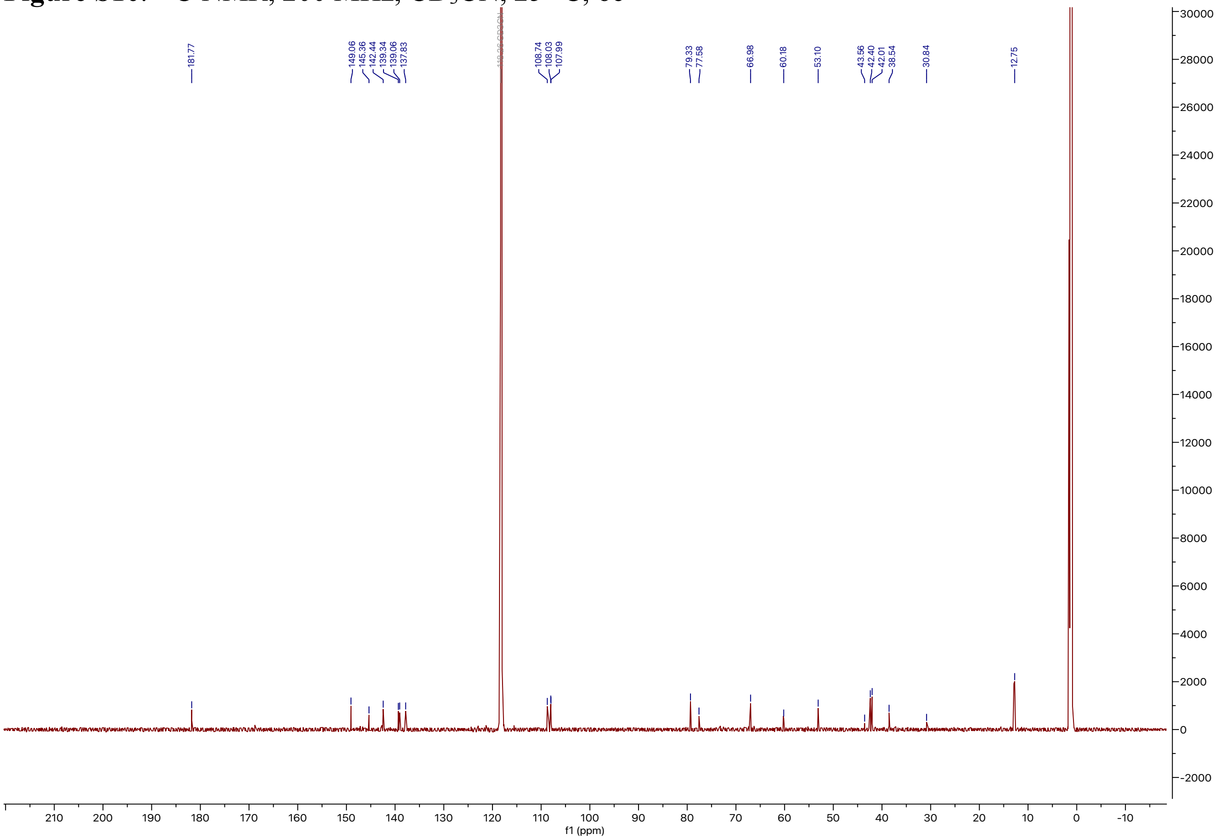

Figure S11: ^{1}H NMR, 800 MHz, CD_3CN, 25 °C, **6f**

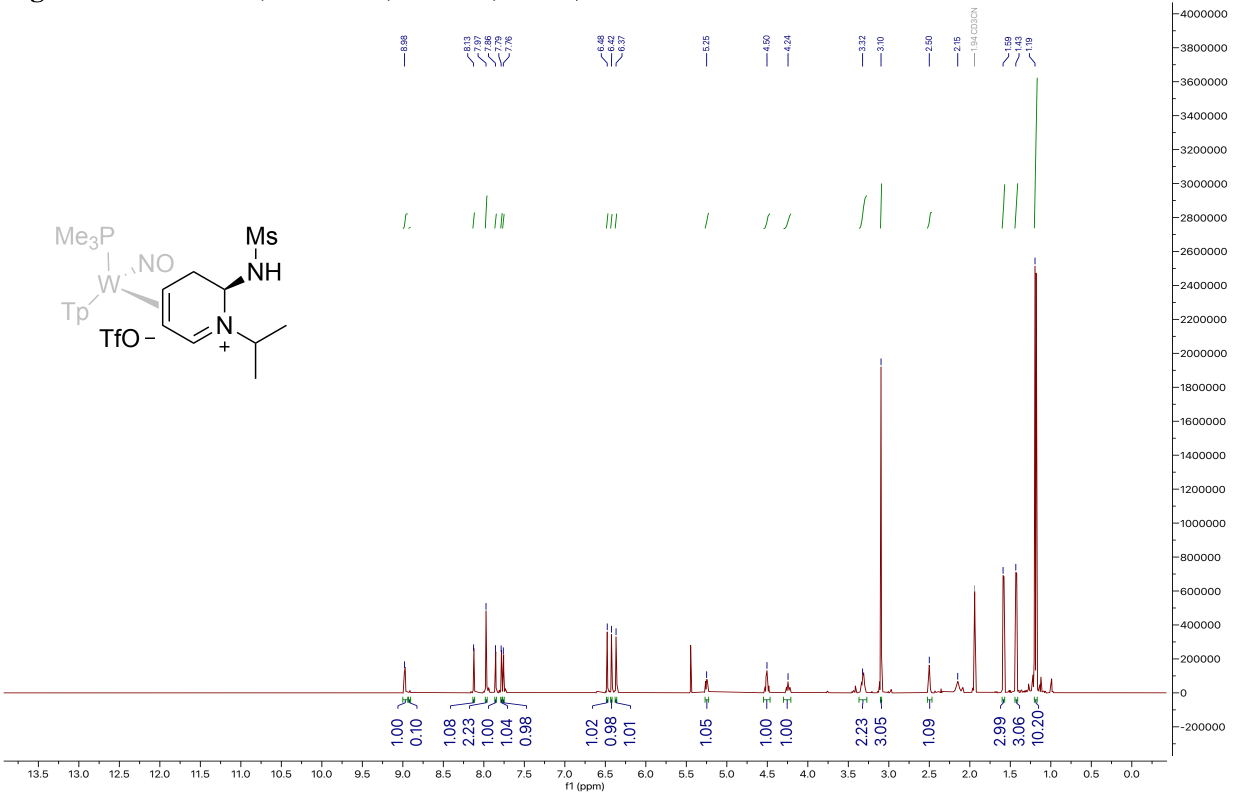

Figure S12: ^{13}C NMR, 200 MHz, CD_3CN, 25 °C, **6f**

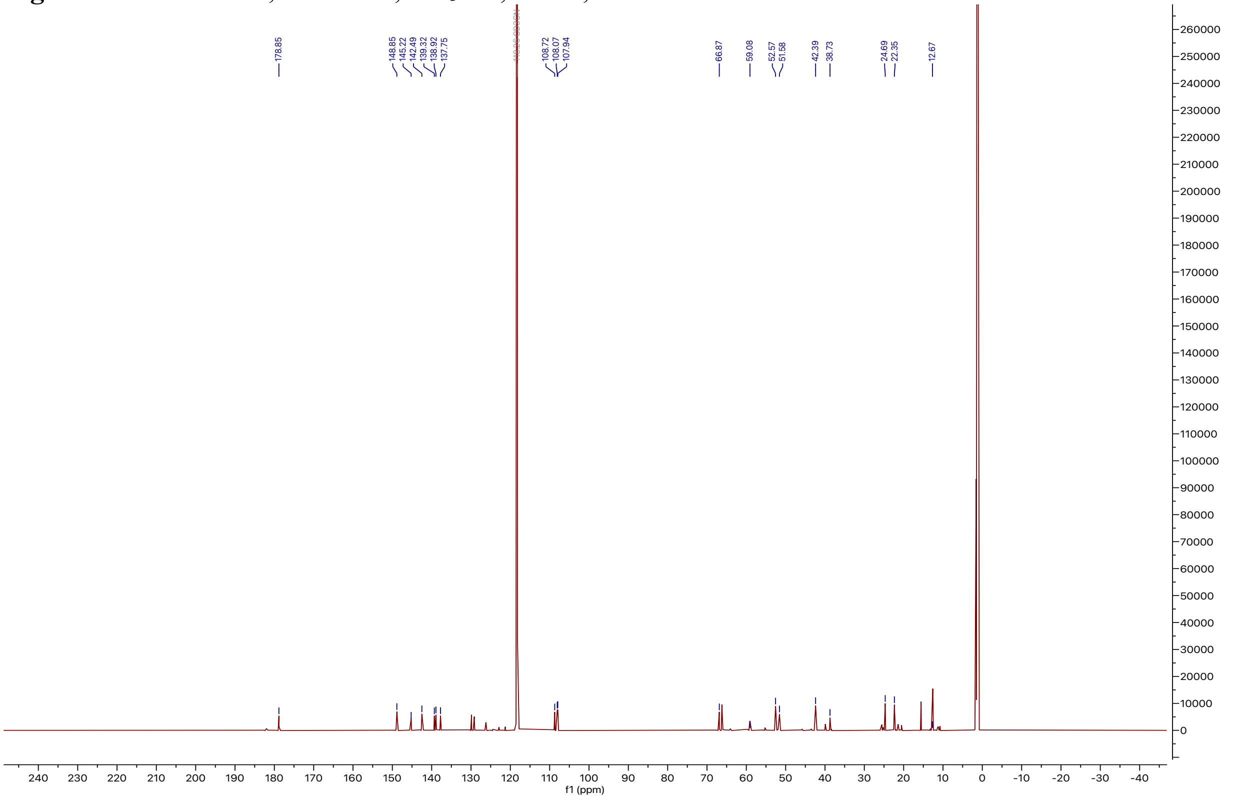

Figure S13: ^{1}H NMR, 800 MHz, CD_3CN, 25 °C, **6g**

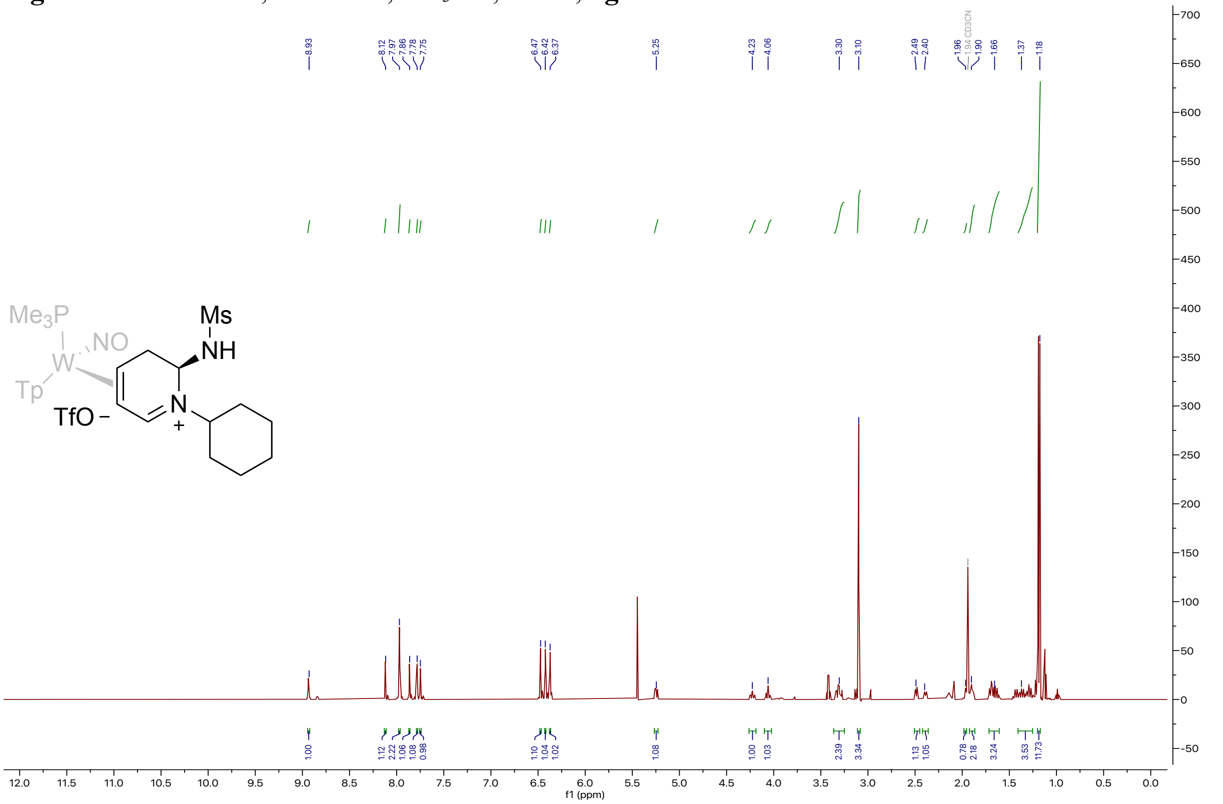

Figure S14: ^{13}C NMR, 200 MHz, CD_3CN, 25 °C, **6g**

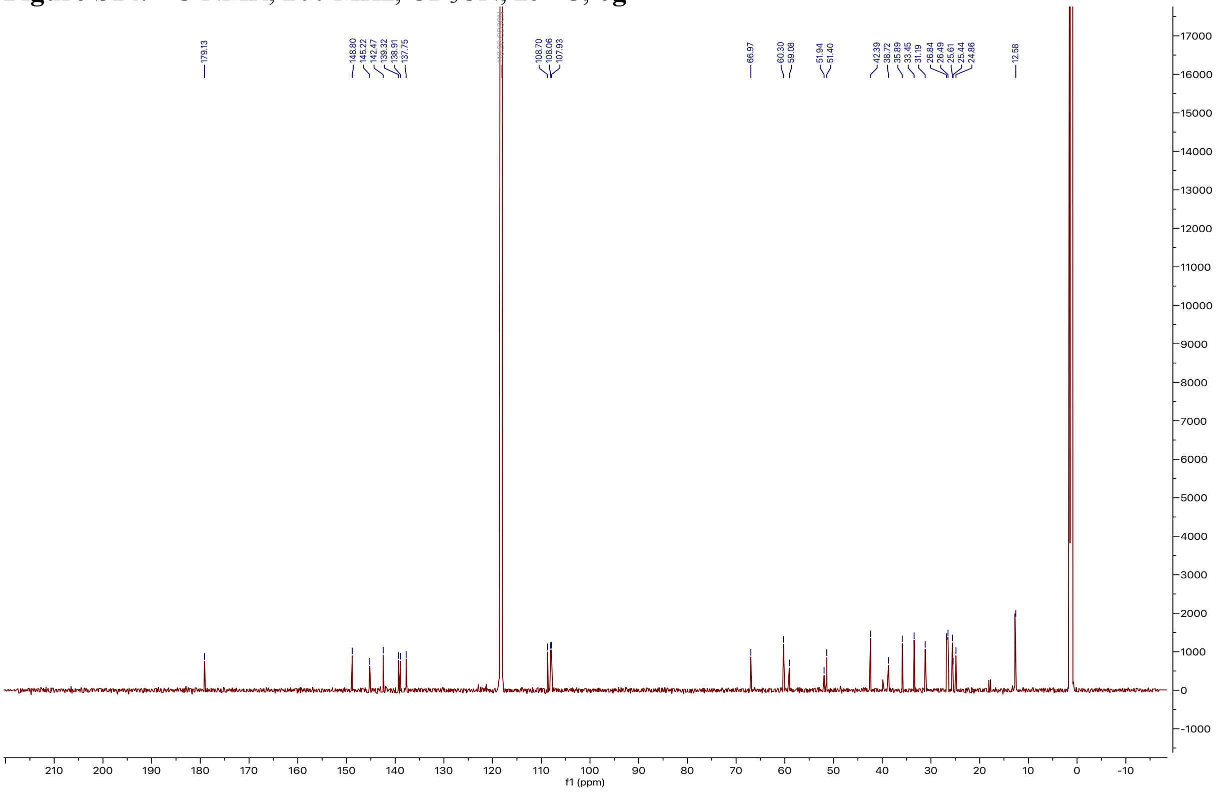

Figure S15: ^{1}H NMR, 800 MHz, CD_2Cl_2, 25 °C, **6h**

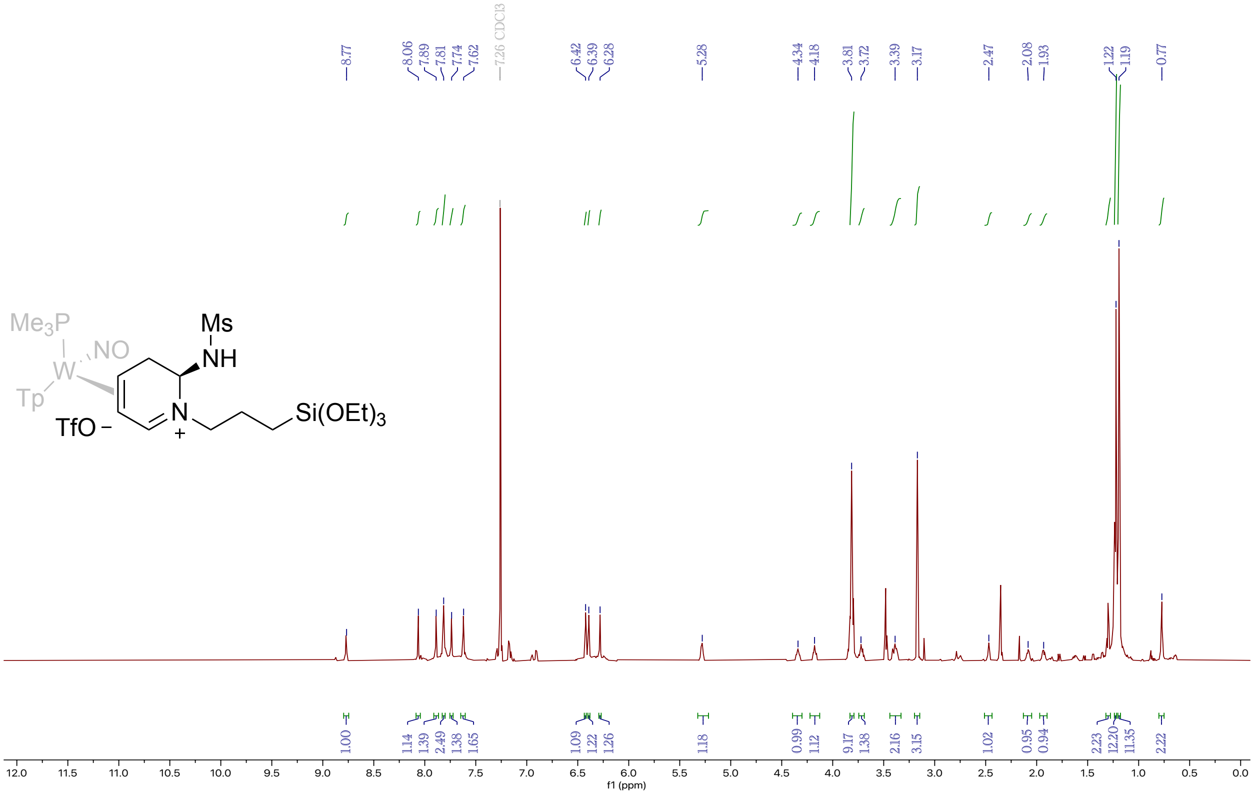

Figure S16: ^{13}C NMR, 200 MHz, CD_2Cl_2, 25 °C, **6h**

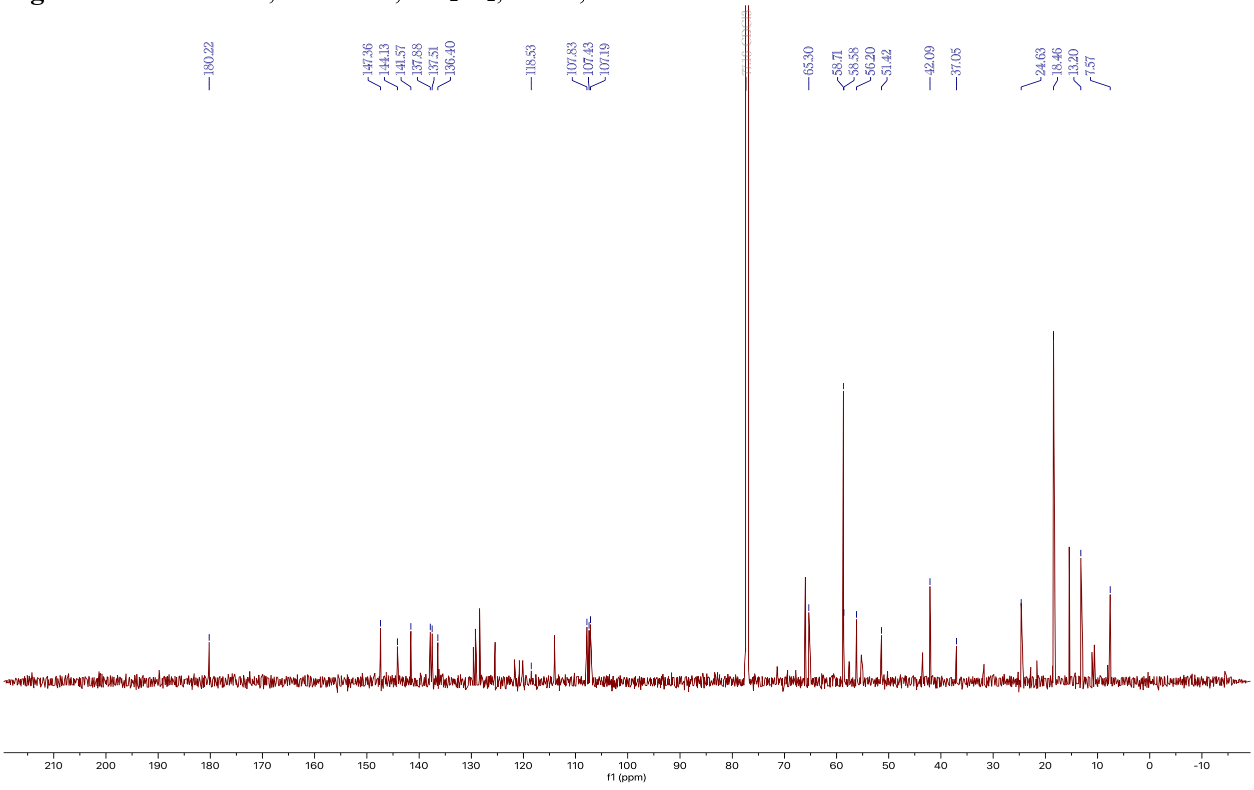

Figure S17: ^{1}H NMR, 800 MHz, CD_3CN, 25 °C, **6i**

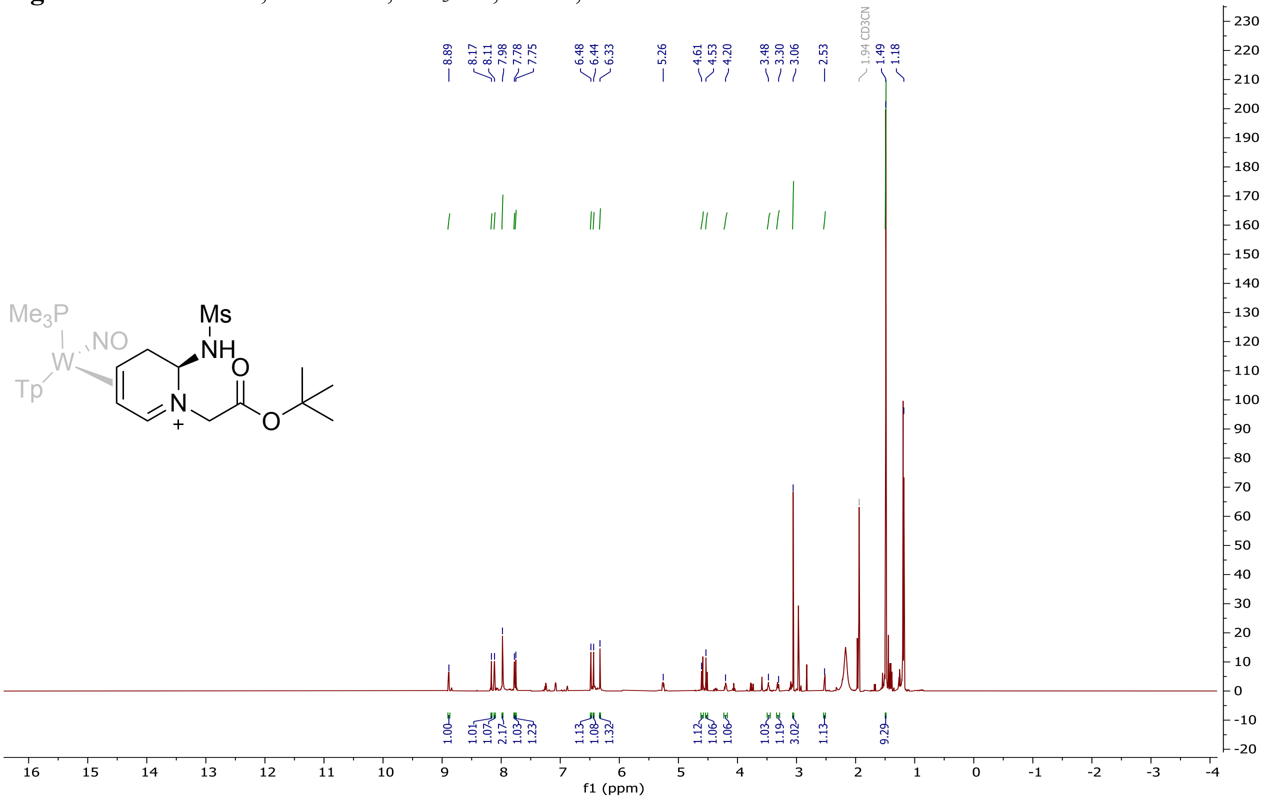

Figure S18: ^{13}C NMR, 200 MHz, CD_3CN, 25 °C, **6i**

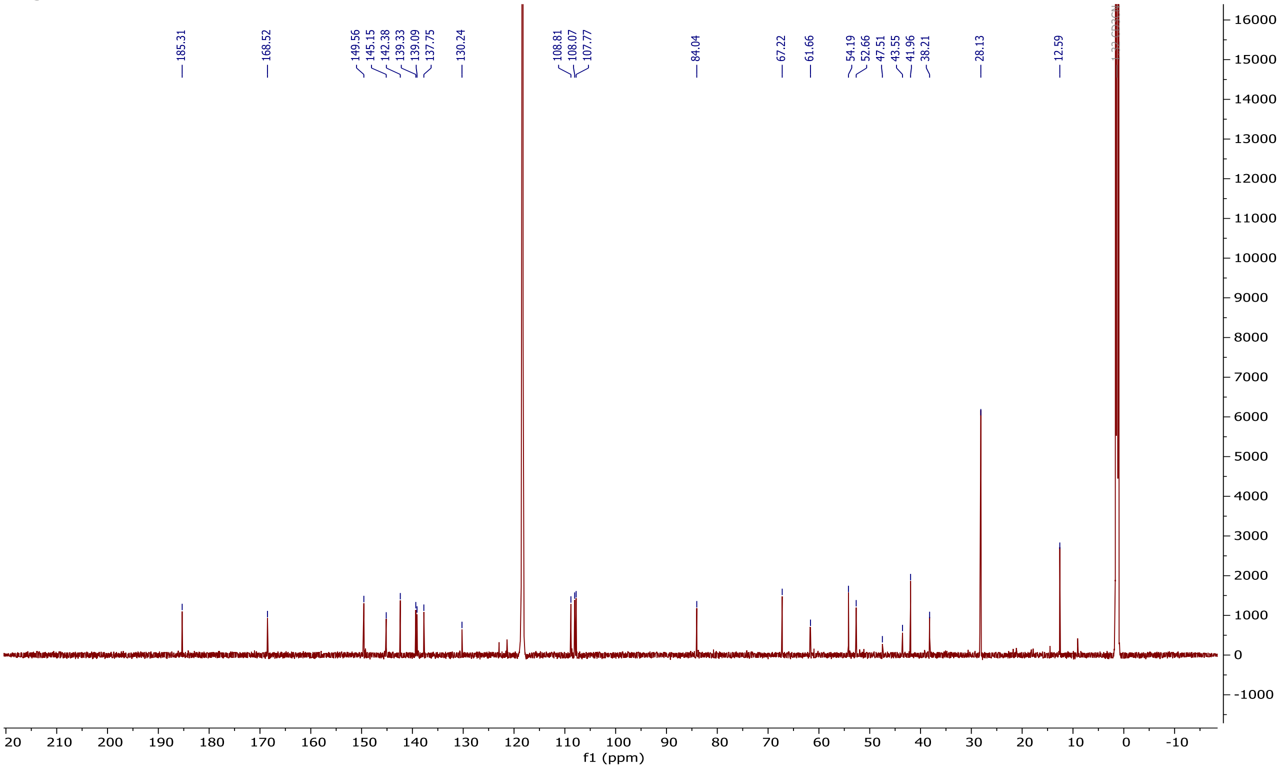

Figure S19: ^{1}H NMR, 800 MHz, CD_3CN, 25 °C, **6j**

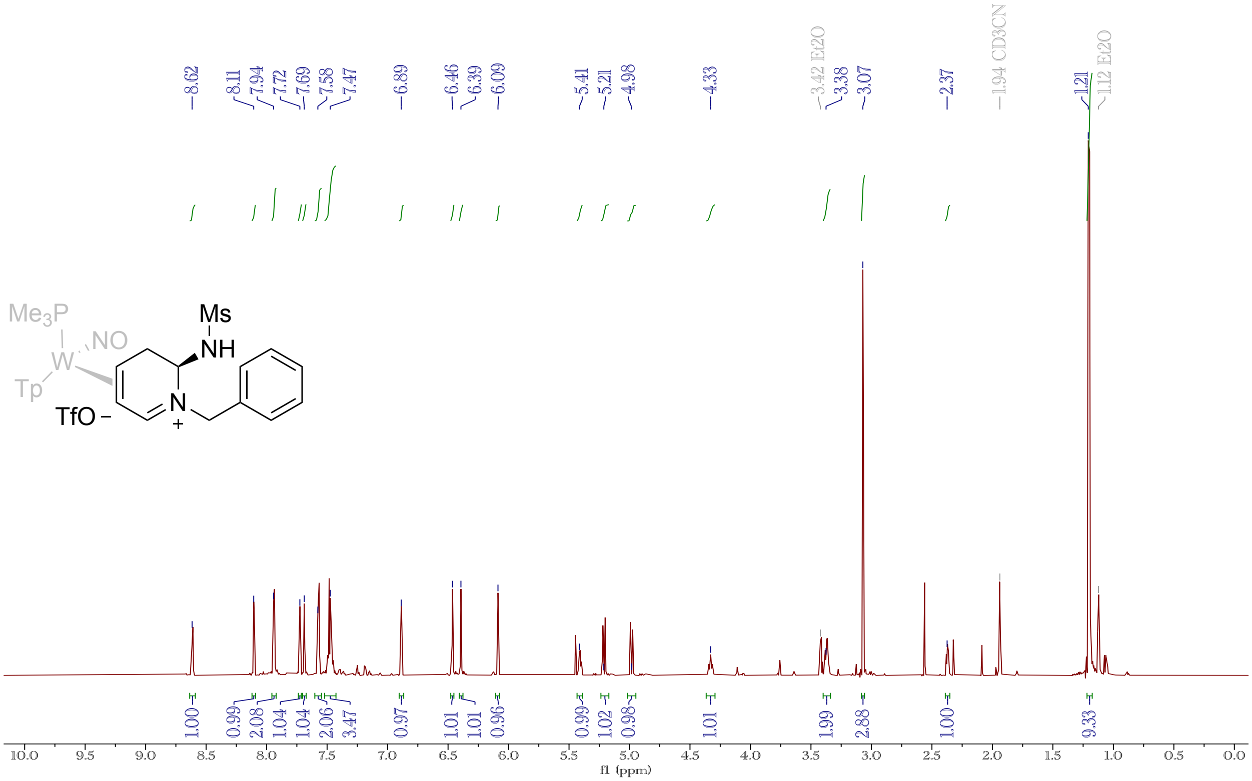

Figure S20: ^{13}C NMR, 200 MHz, CD_3CN, 25 °C, **6j**

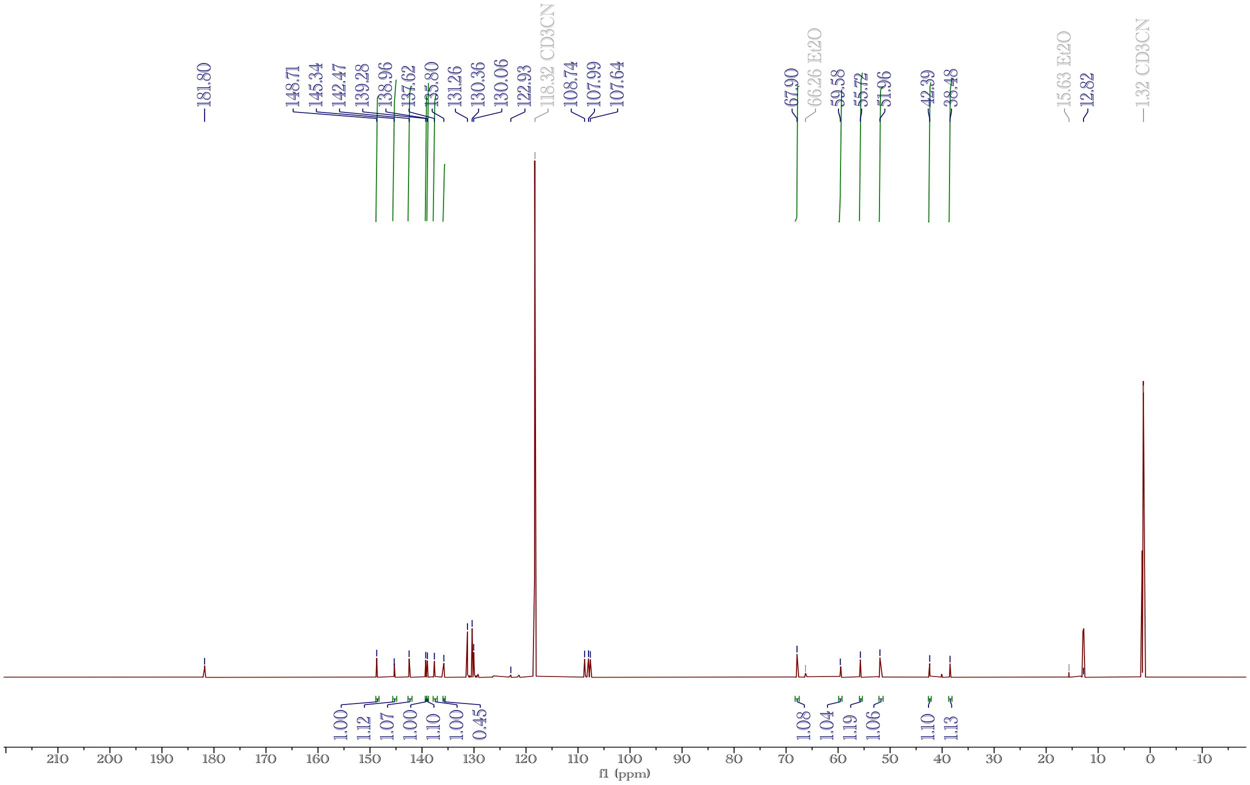

Figure S21: ^{1}H NMR, 800 MHz, CD_3CN, 25 °C, **6k**

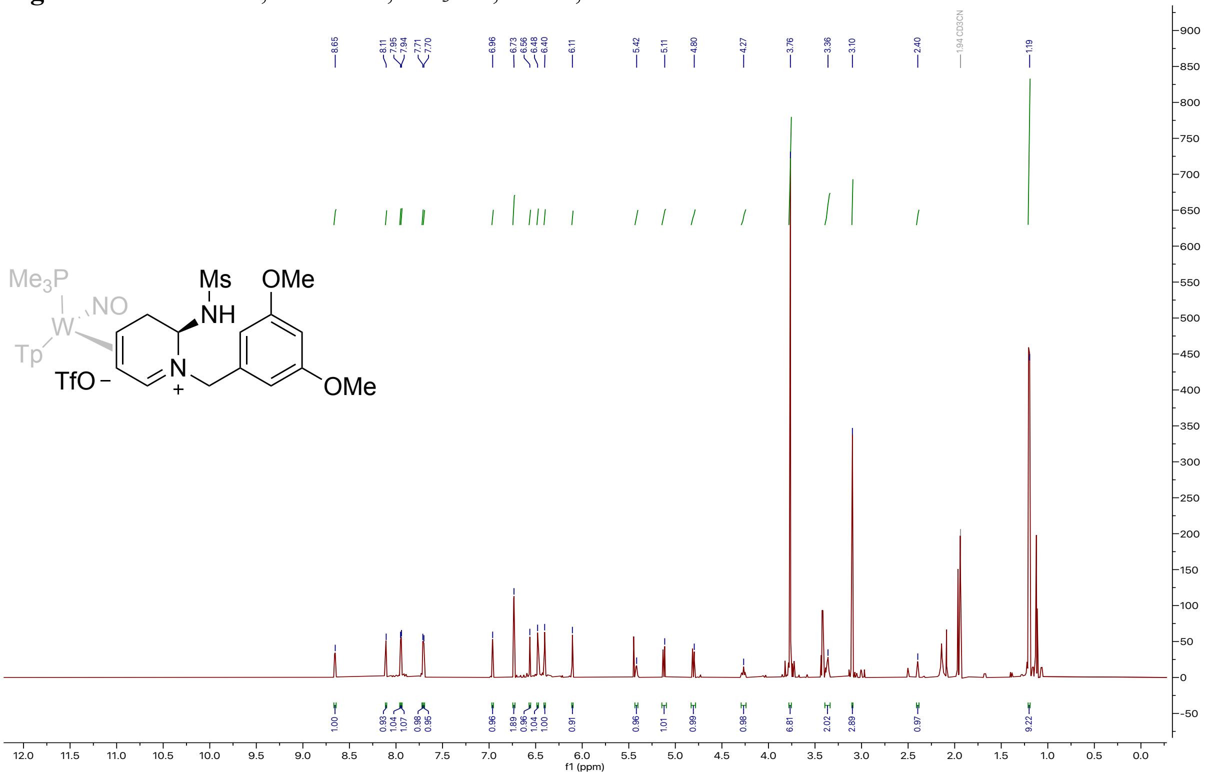

Figure S22: ^{13}C NMR, 200 MHz, CD_3CN, 25 °C, **6k**

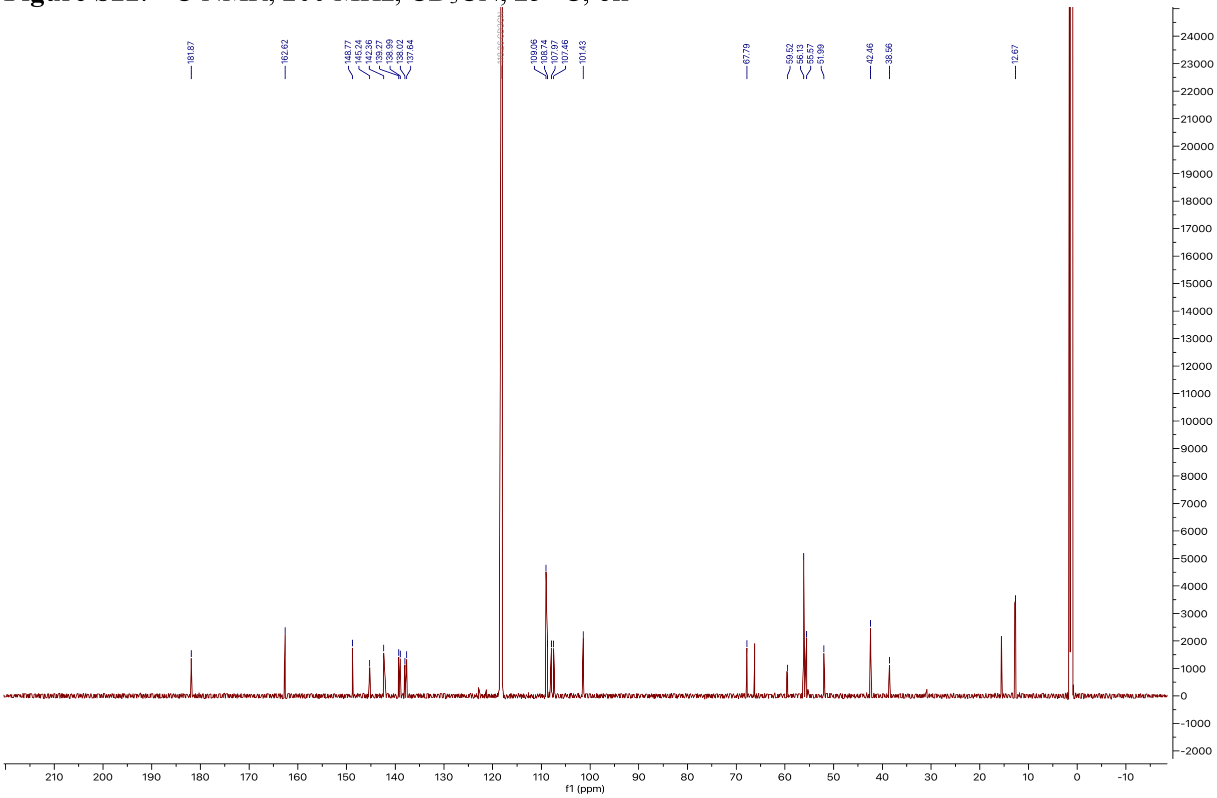

Figure S23: ^{1}H NMR, 800 MHz, CD_2Cl_2, 25 °C, **6l**

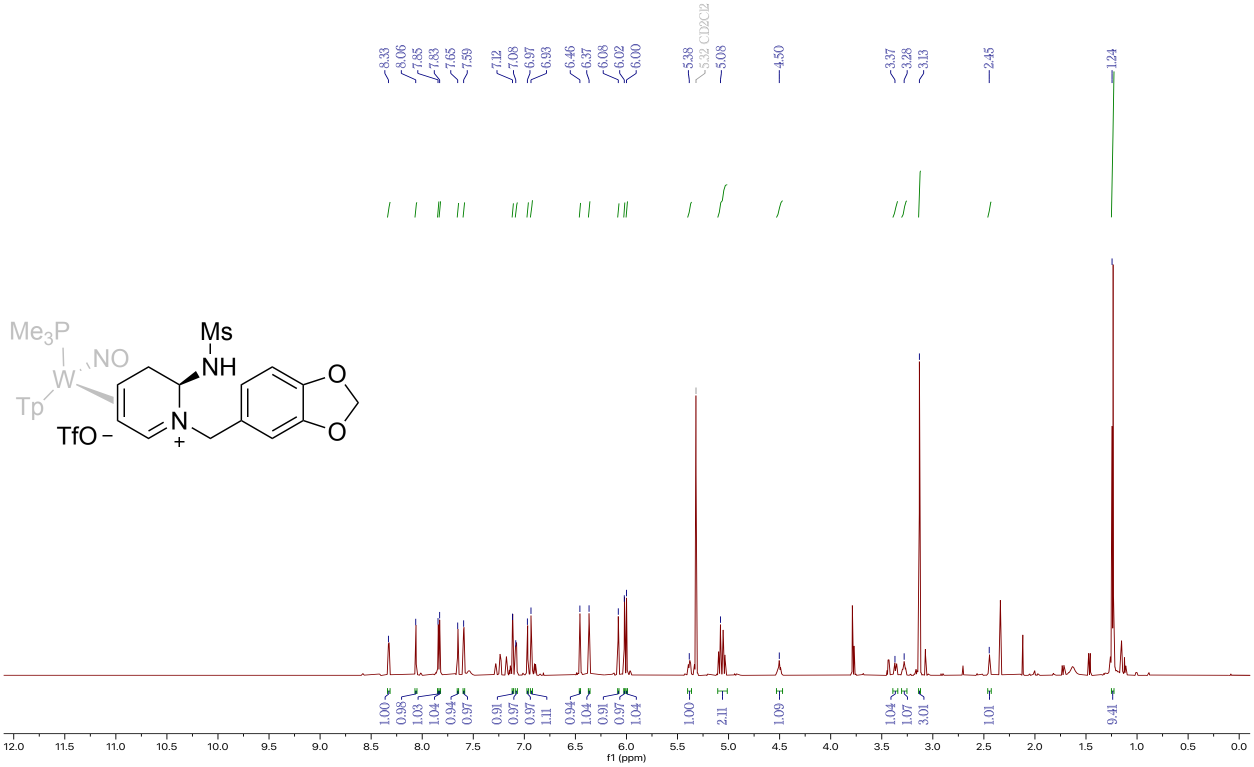

Figure S24: ^{13}C NMR, 200 MHz, CD_2Cl_2, 25 °C, **6l**

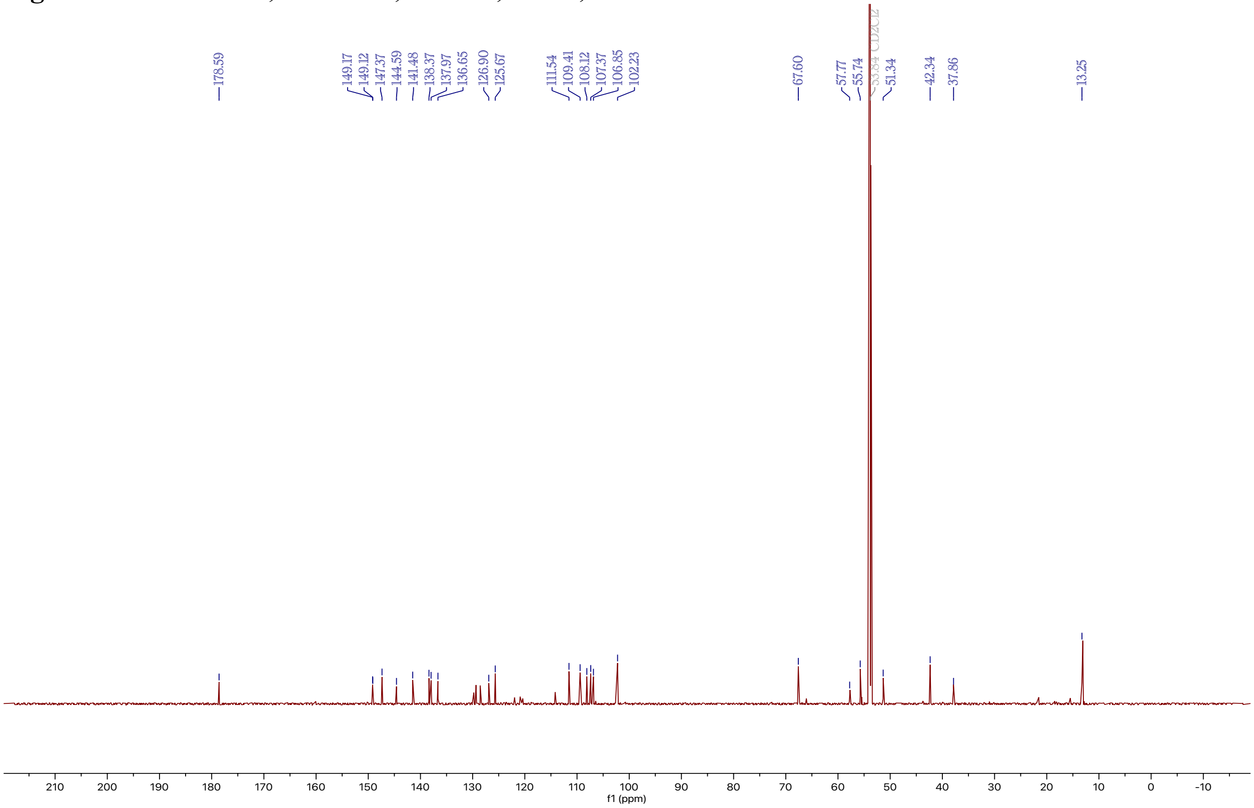

Figure S25: ^{1}H NMR, 800 MHz, CD_3CN, 25 °C, **6m**

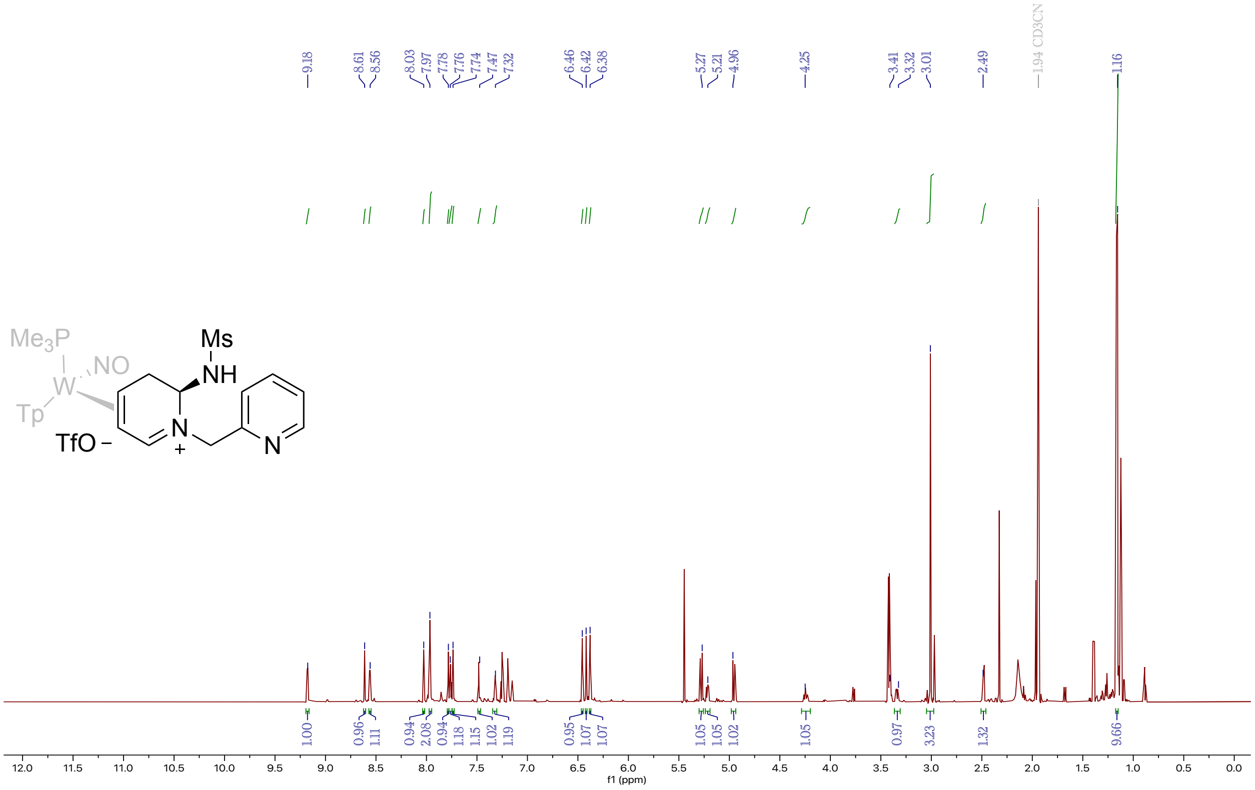

Figure S26: ^{13}C NMR, 200 MHz, CD_3CN, 25 °C, **6m**

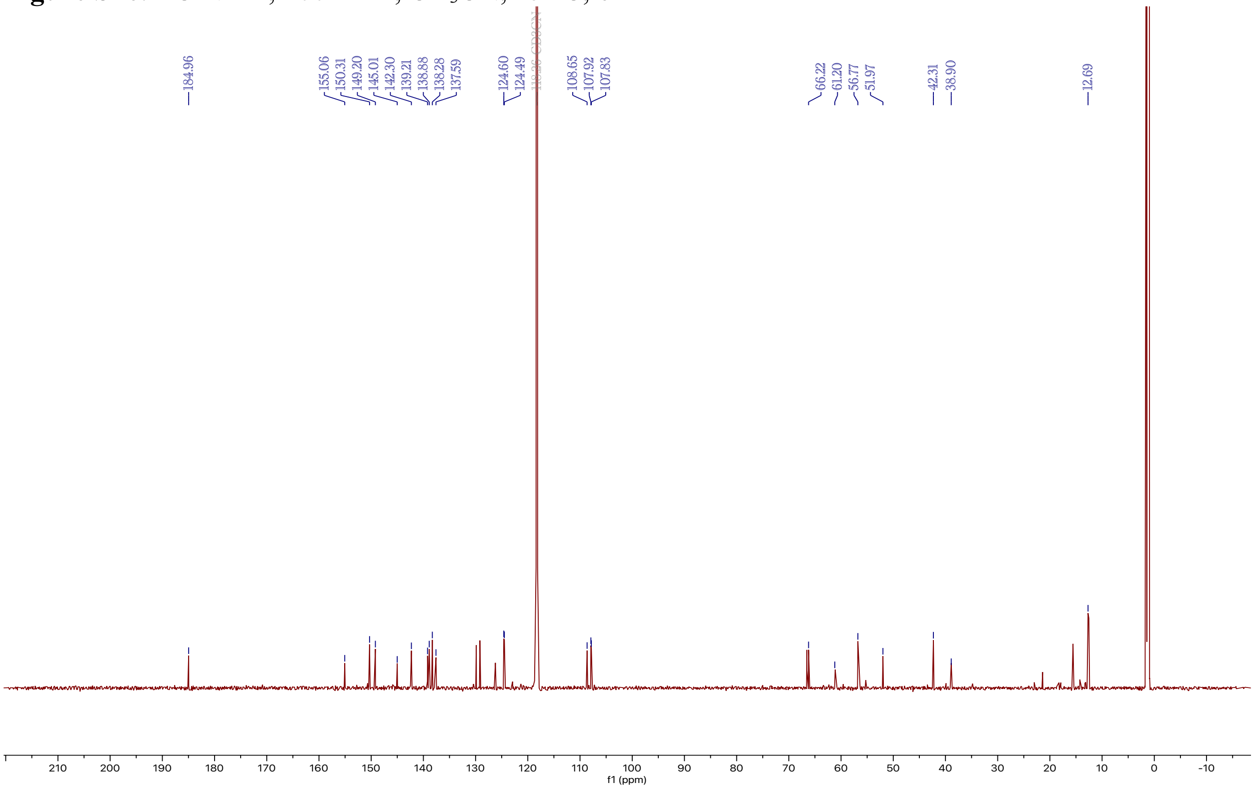

Figure S27: ^{1}H NMR, 800 MHz, CD_3CN, 25 °C, **6n**

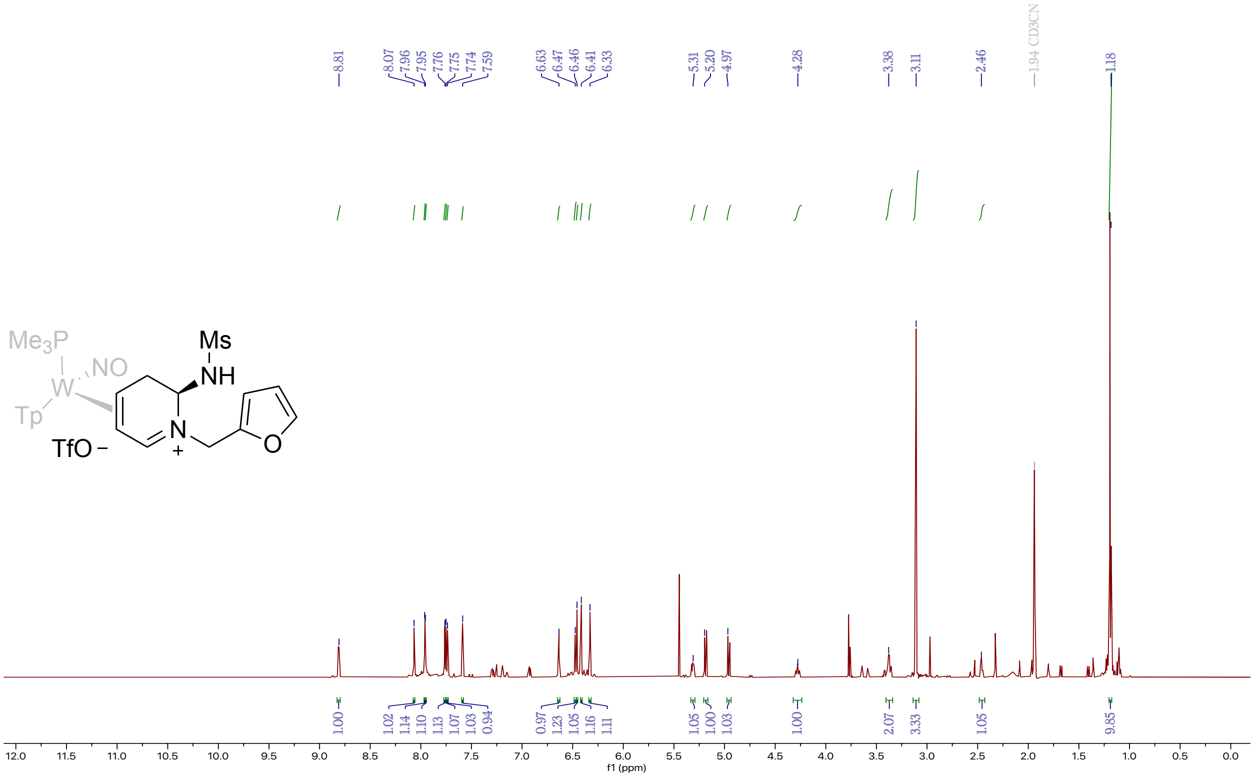

Figure S28: ^{13}C NMR, 200 MHz, CD_3CN, 25 °C, **6n**

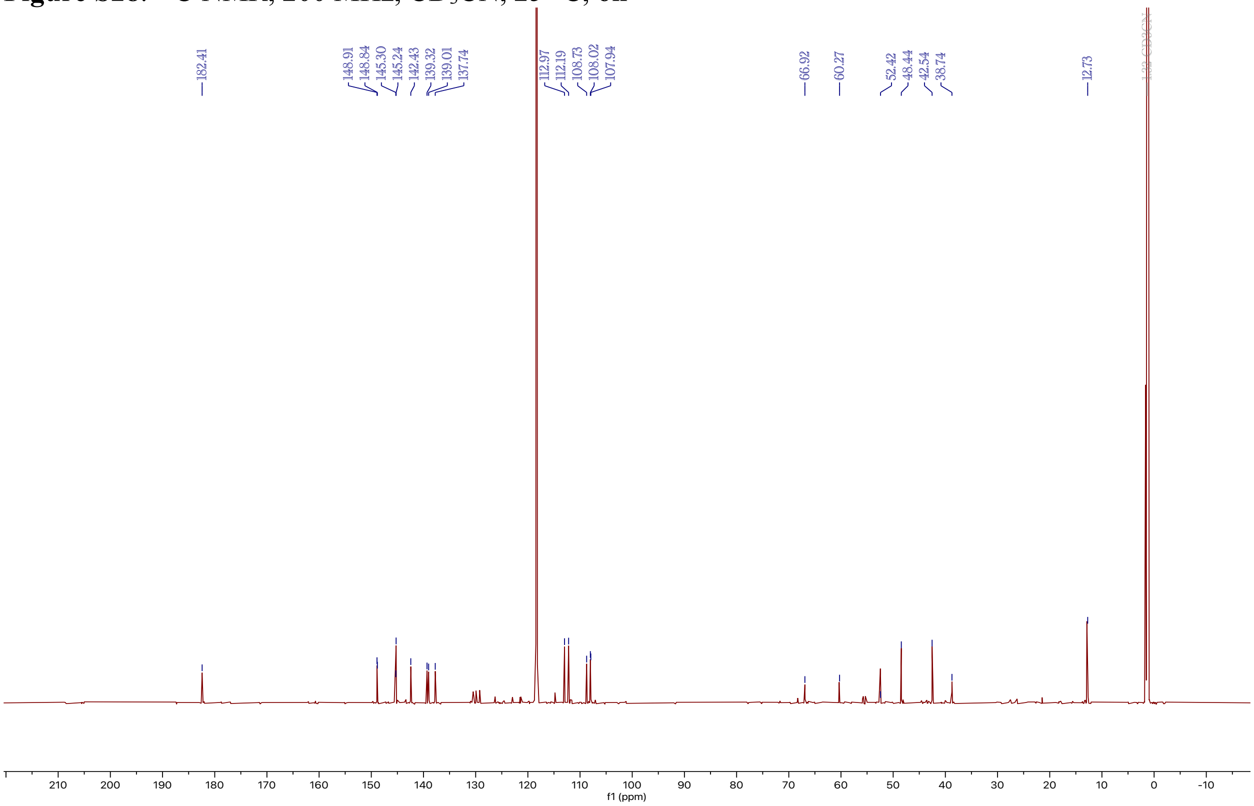

Figure S29: ^{1}H NMR, 800 MHz, CD_3CN, 25 °C, **6o**

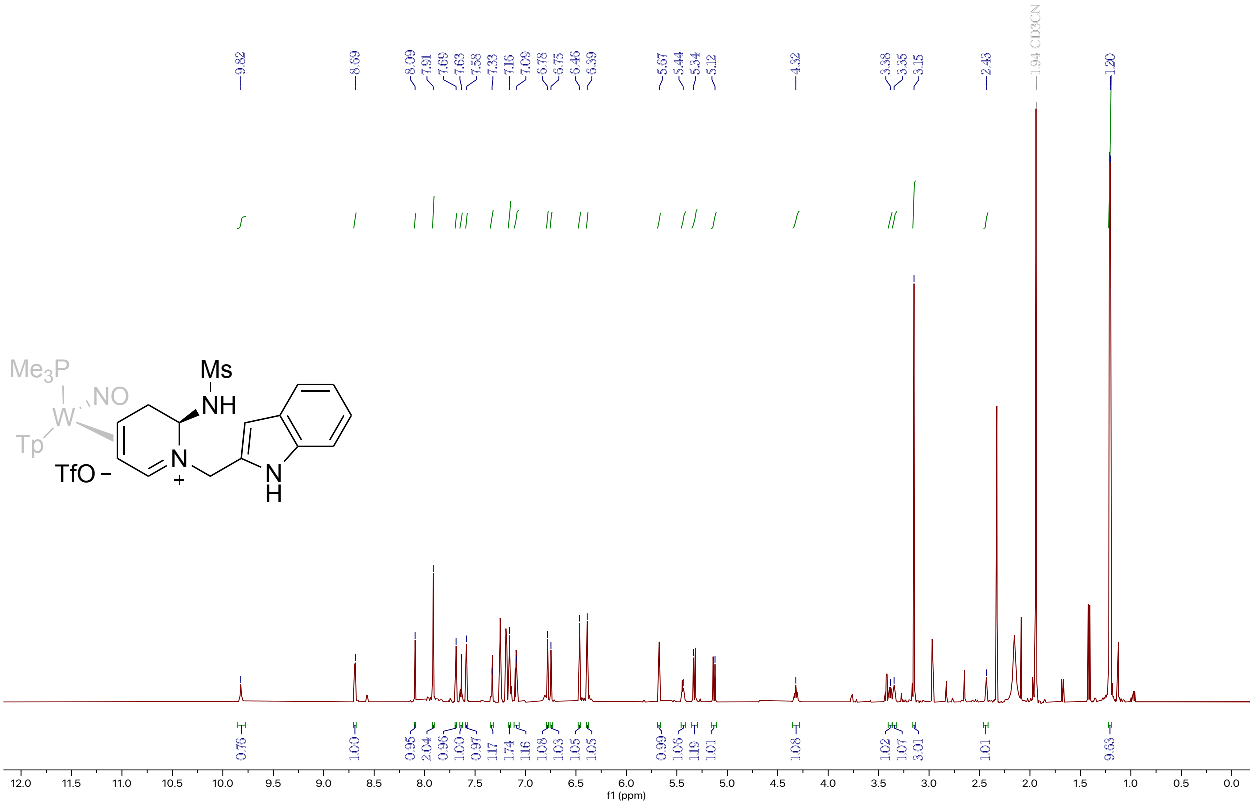

Figure S30: ^{13}C NMR, 200 MHz, CD_3CN, 25 °C, **6o**

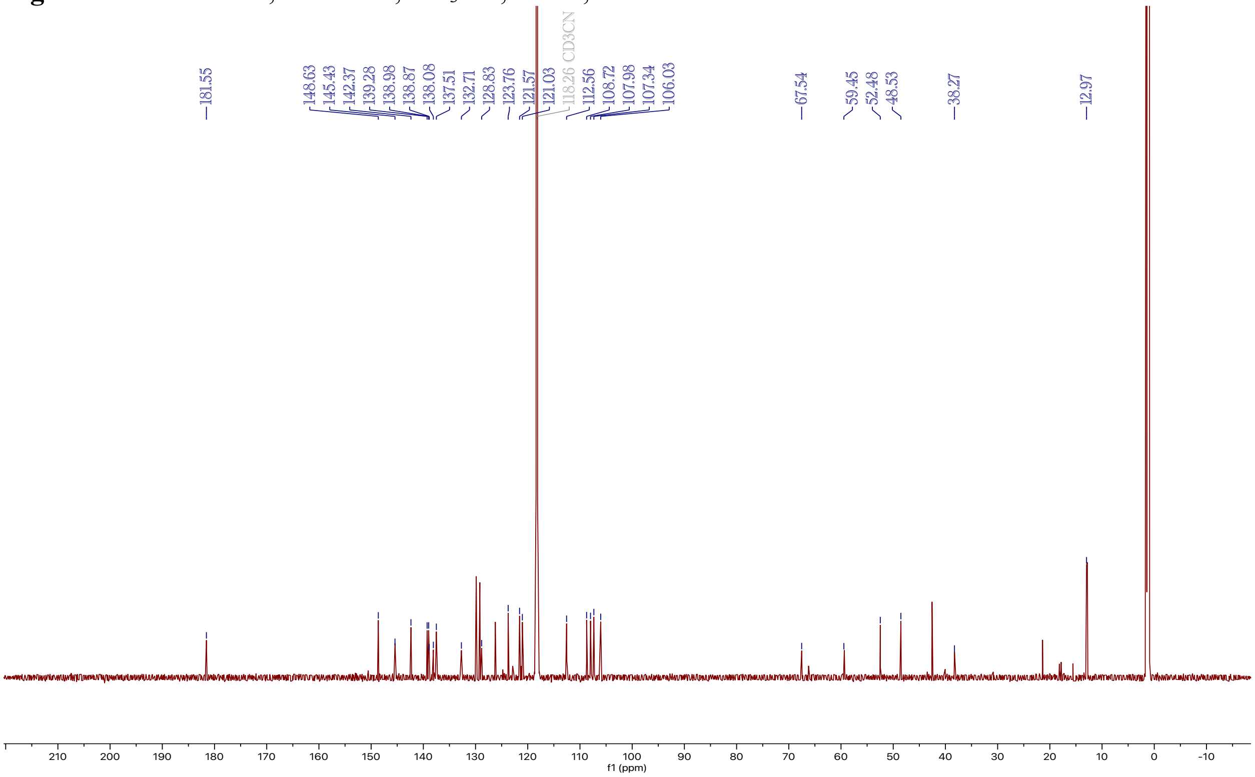

Figure S31: ^{1}H NMR, 800 MHz, CD_3CN, 25 °C, **6p**

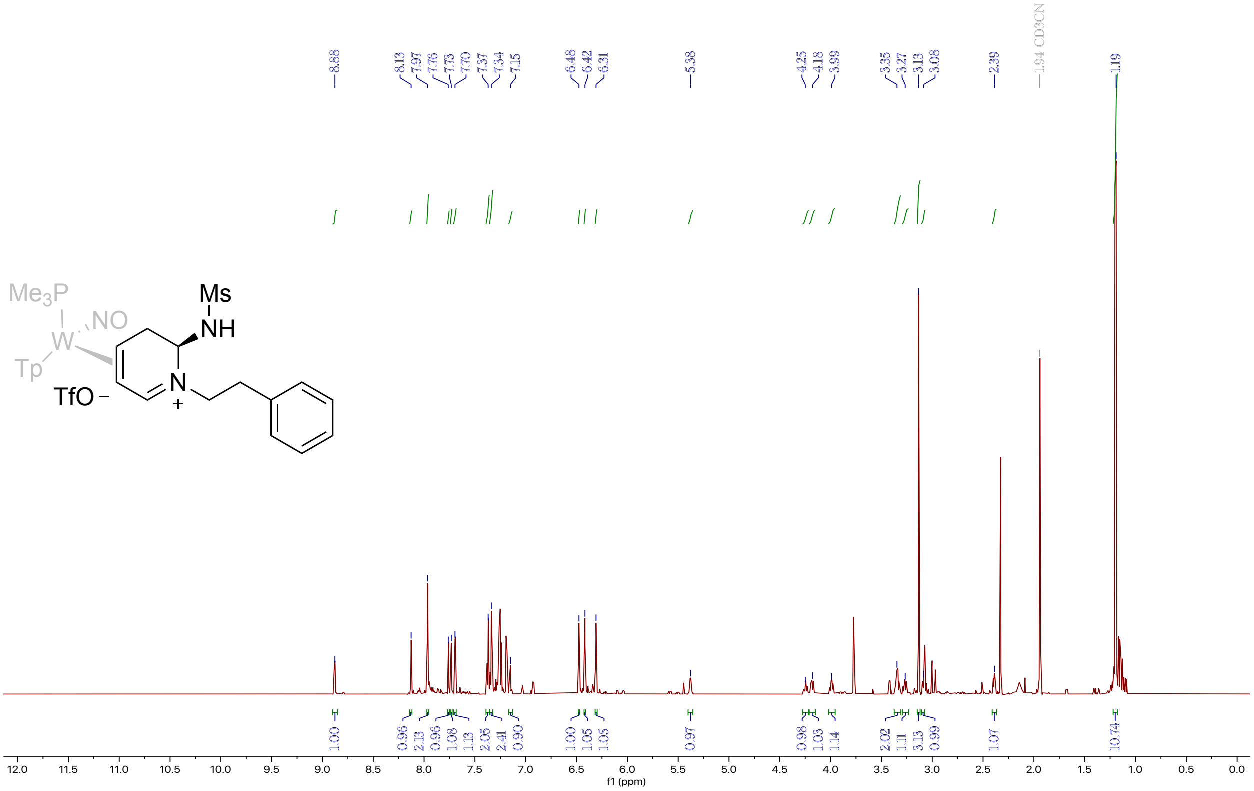

Figure S32: ^{13}C NMR, 200 MHz, CD_3CN, 25 °C, **6p**

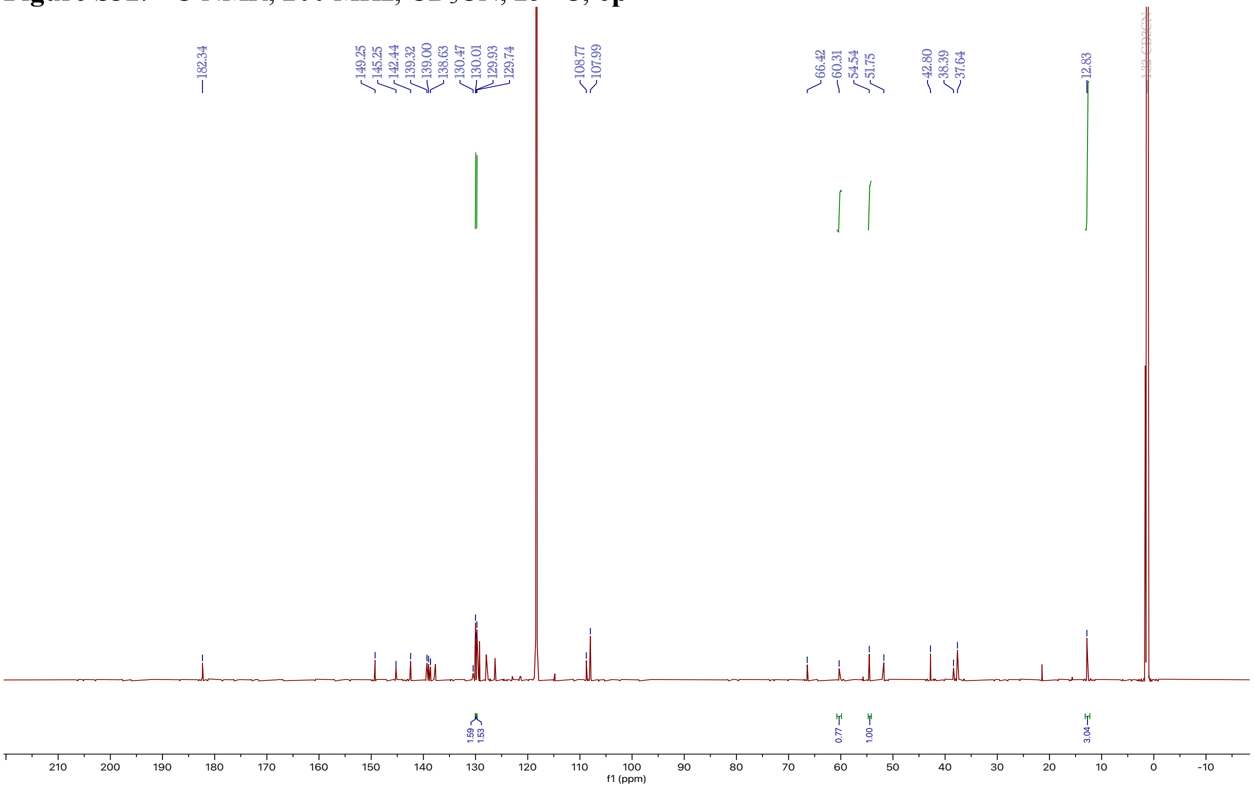

Figure S33: ^{1}H NMR, 800 MHz, CD_3CN, 25 °C, **6q**

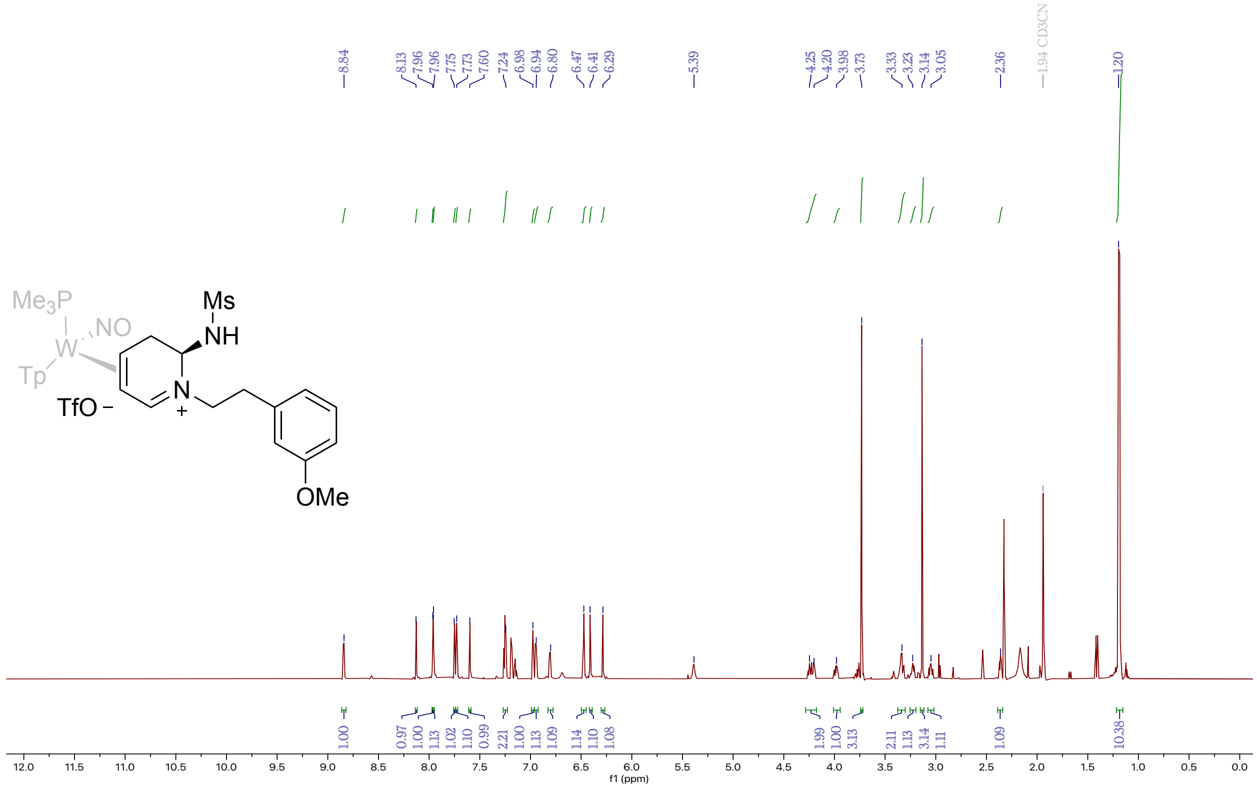

Figure S34: ^{13}C NMR, 200 MHz, CD_3CN, 25 °C, **6q**

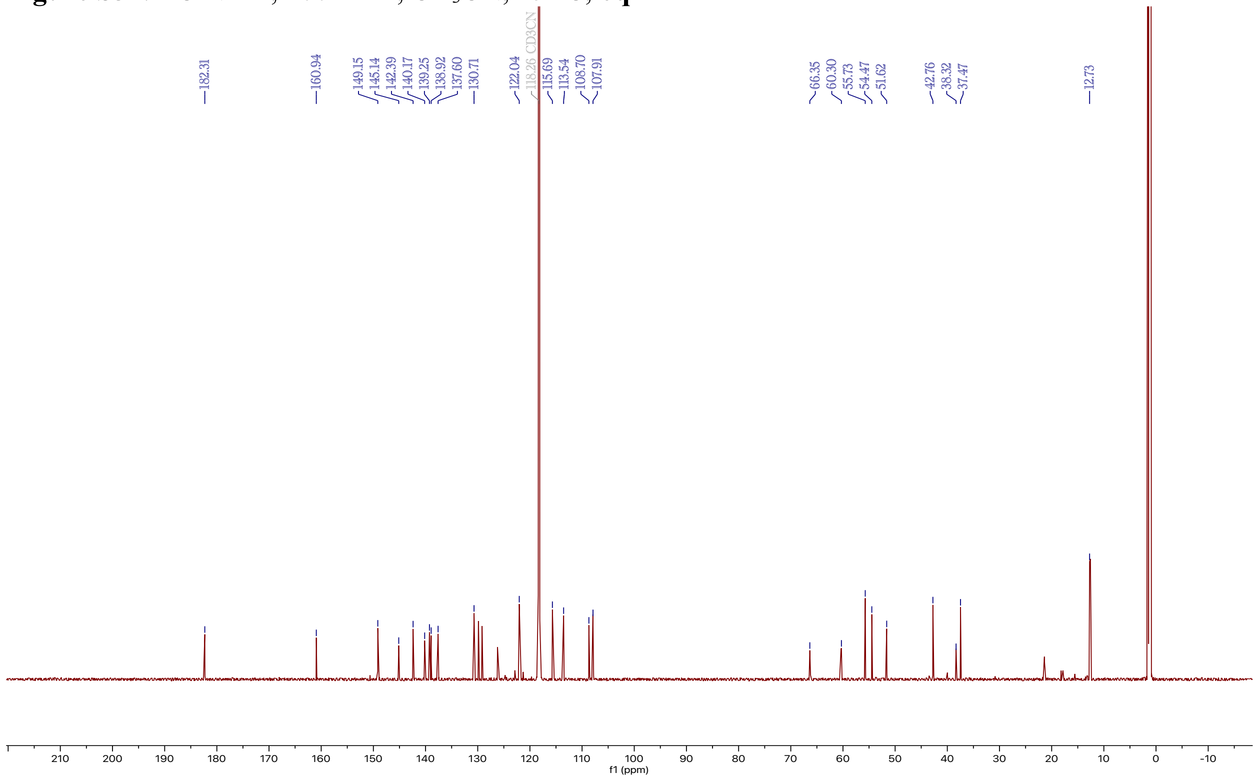

Figure S35: ^{1}H NMR, 800 MHz, CD_3CN, 25 °C, **6r**

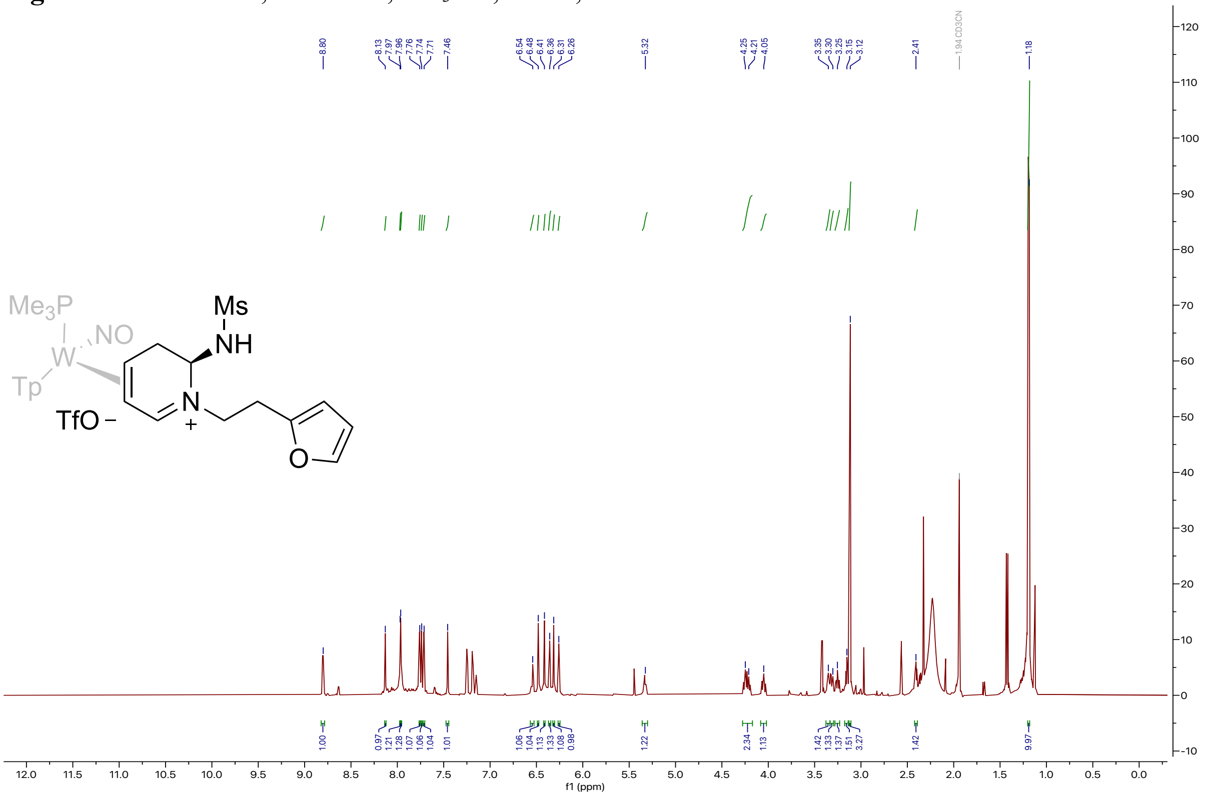

Figure S36: ^{13}C NMR, 200 MHz, CD_3CN, 25 °C, **6r**

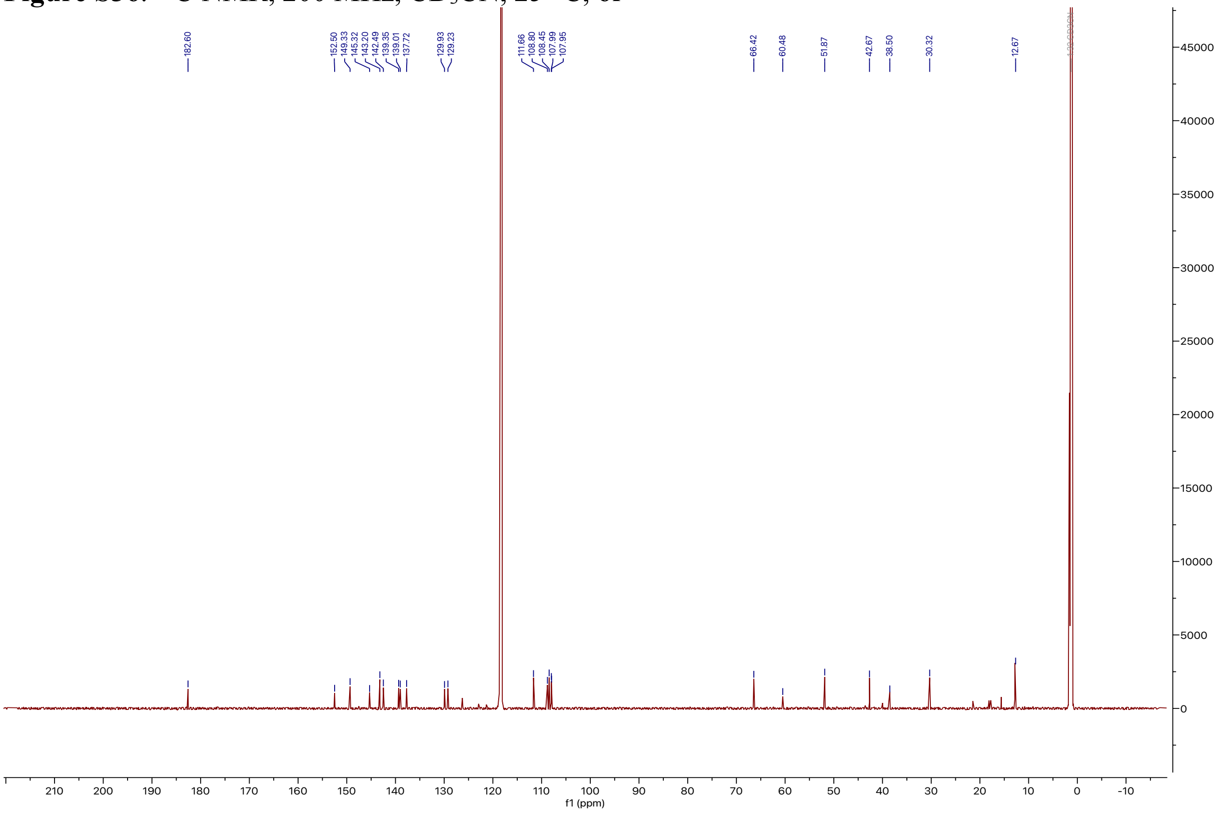

Figure S37: ^{1}H NMR, 800 MHz, CD_3CN, 25 °C, **6s**

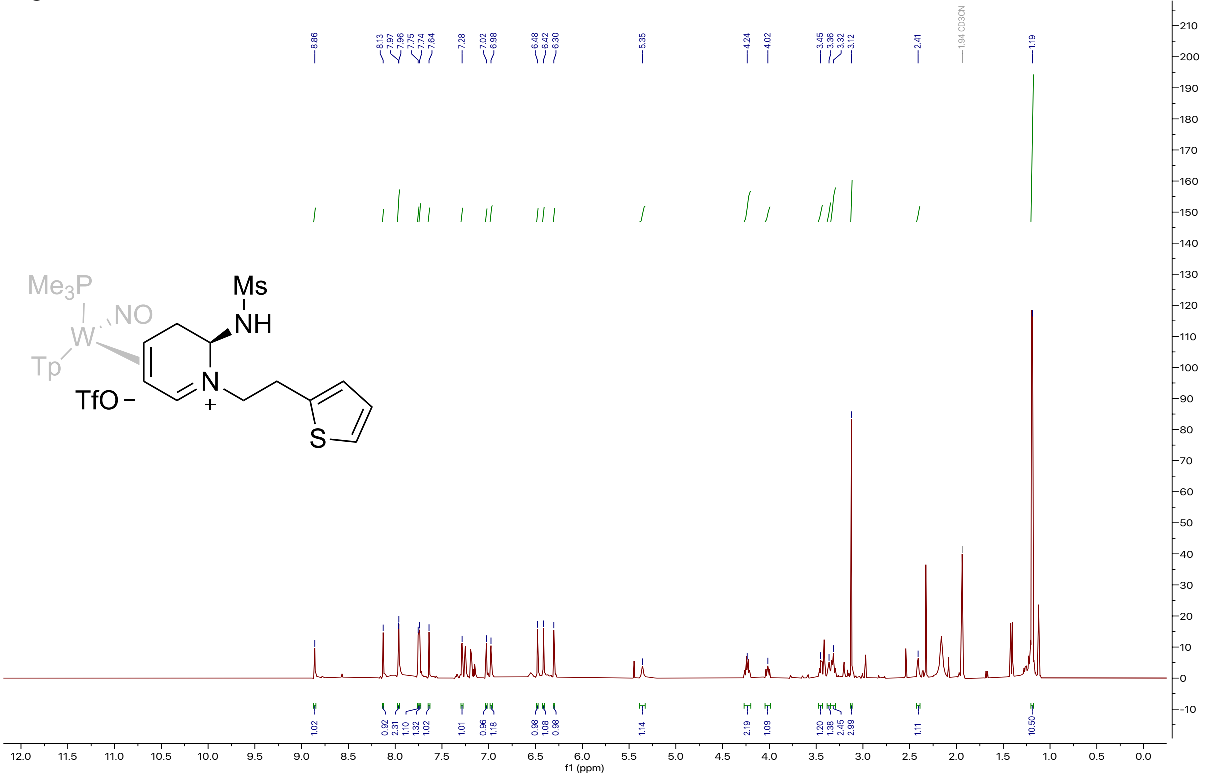

Figure S38: ^{13}C NMR, 200 MHz, CD_3CN, 25 °C, **6s**

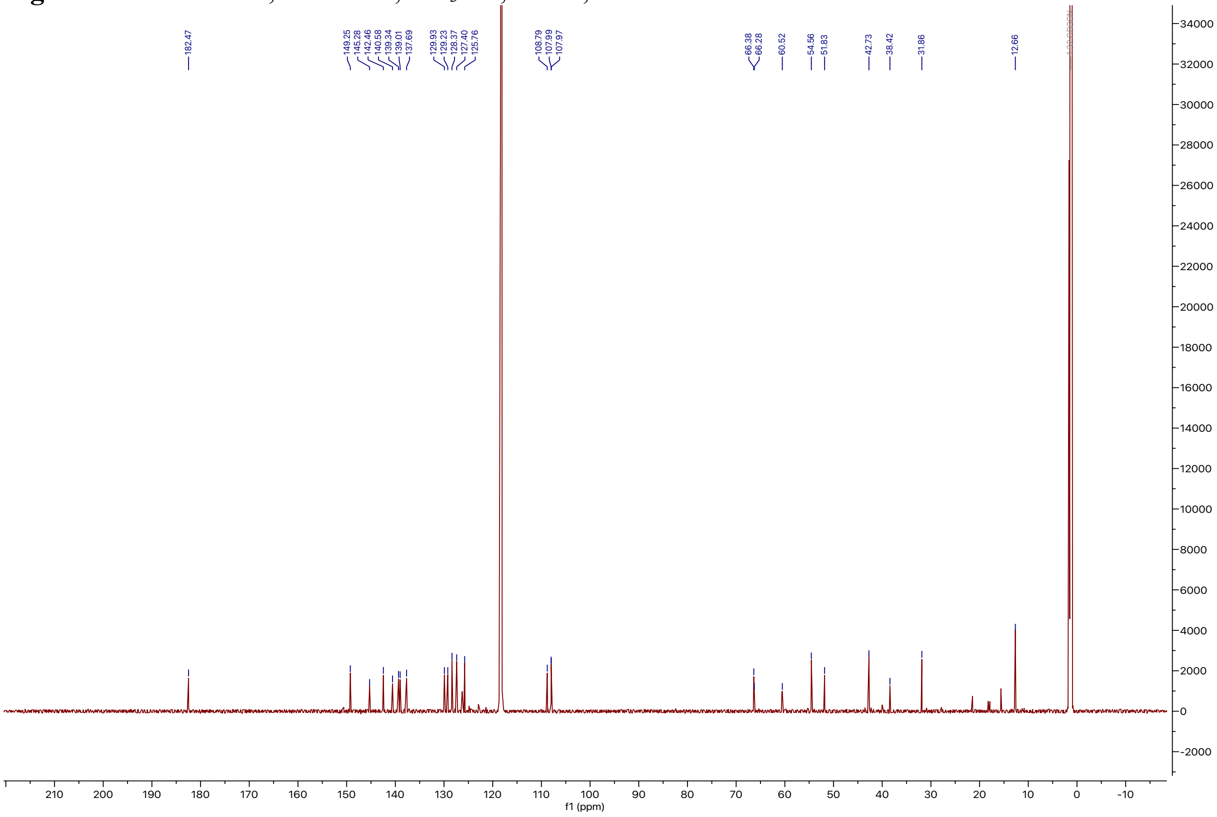

Figure S39: ^{1}H NMR, 800 MHz, CD_3CN, 25 °C, **6t**

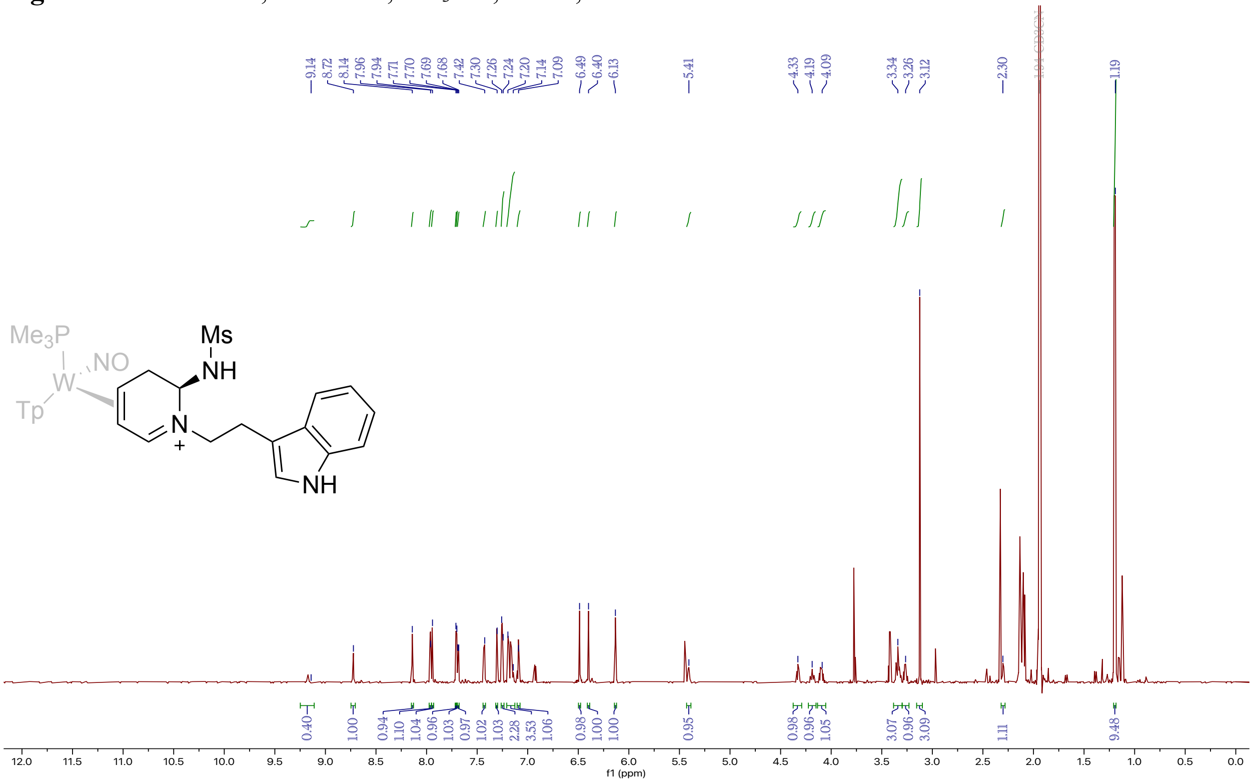

Figure S40: ^{13}C NMR, 200 MHz, CD_3CN, 25 °C, **6t**

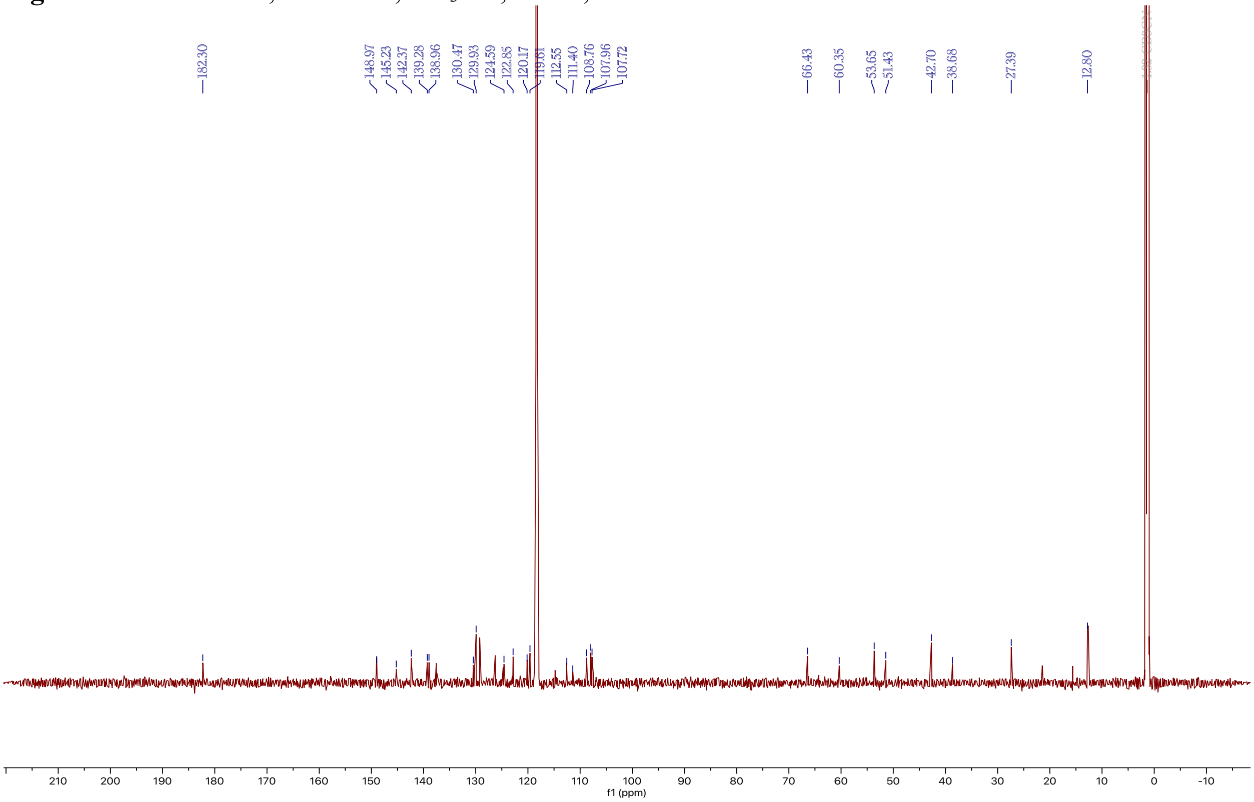

Figure S41: ^{1}H NMR, 800 MHz, CD_3CN, 25 °C, **6u**

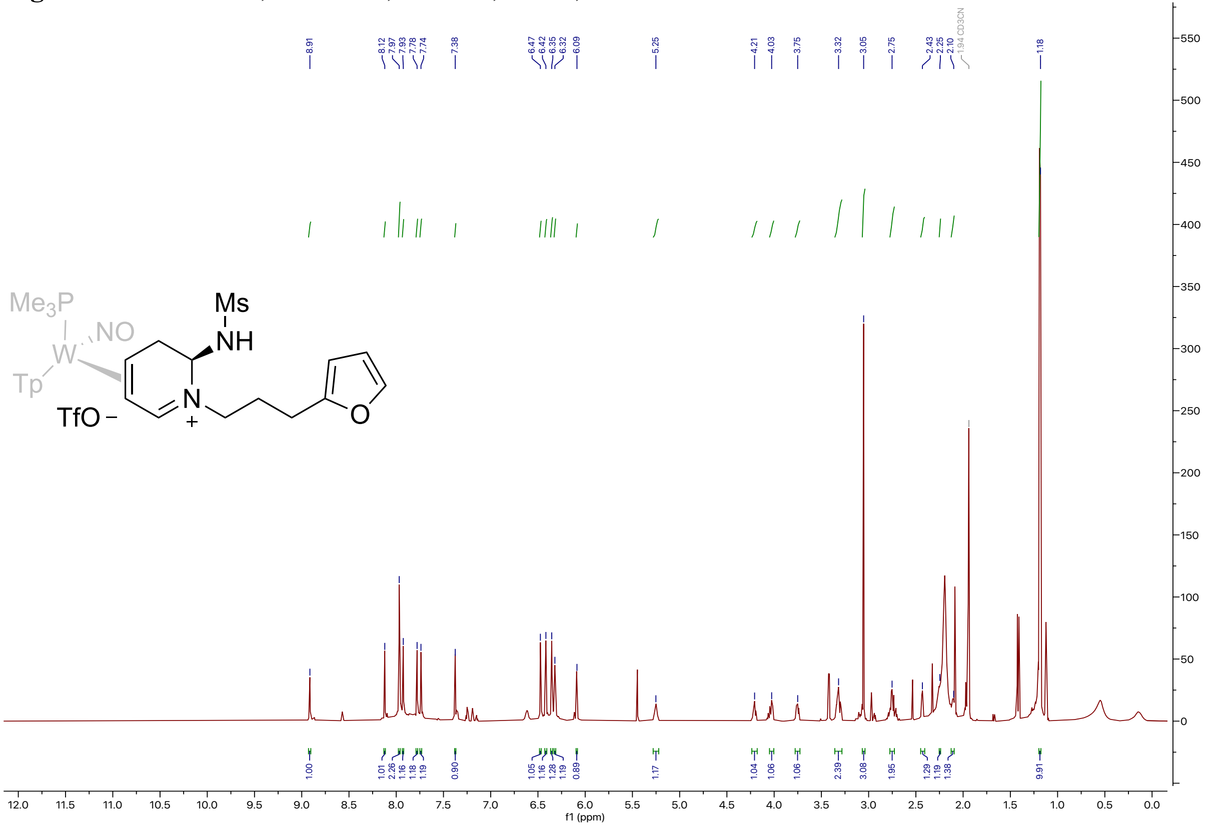

Figure S42: ^{13}C NMR, 200 MHz, CD_3CN, 25 °C, **6u**

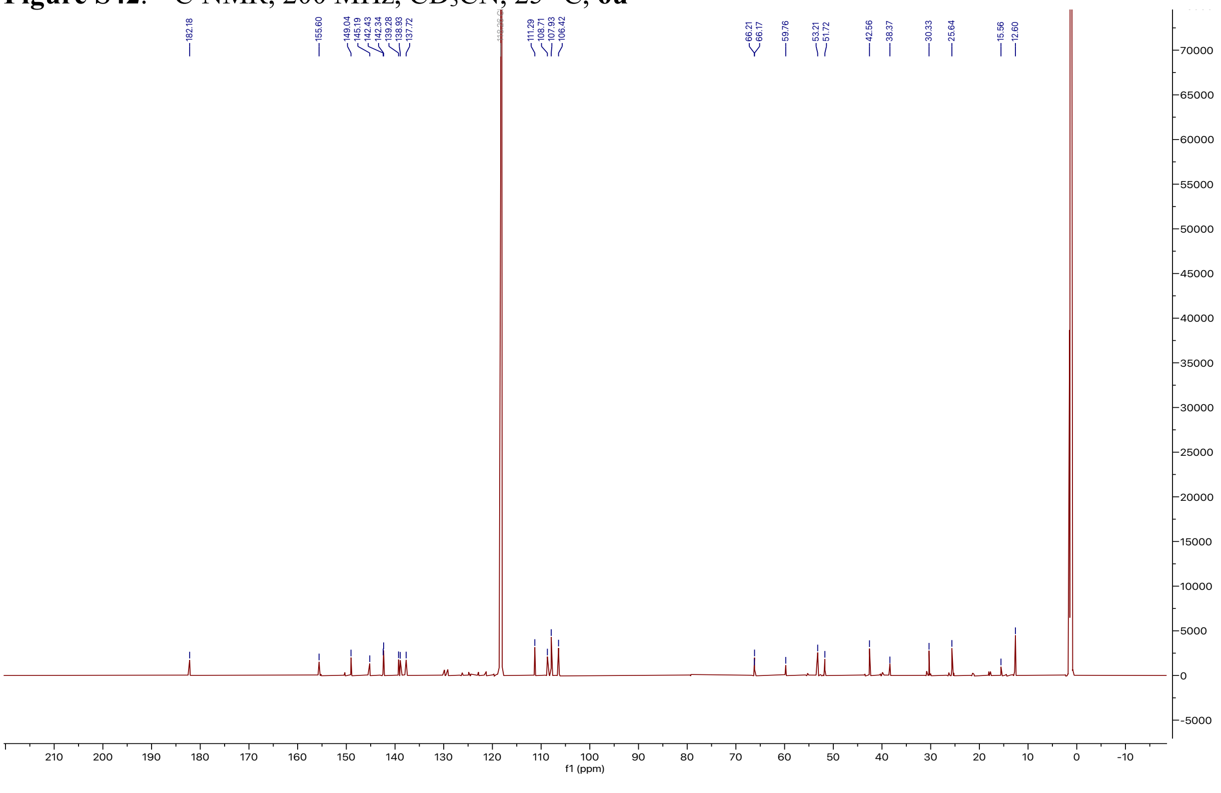

Figure S43: ^{1}H NMR, 800 MHz, CD_3CN, 25 °C, **6v**

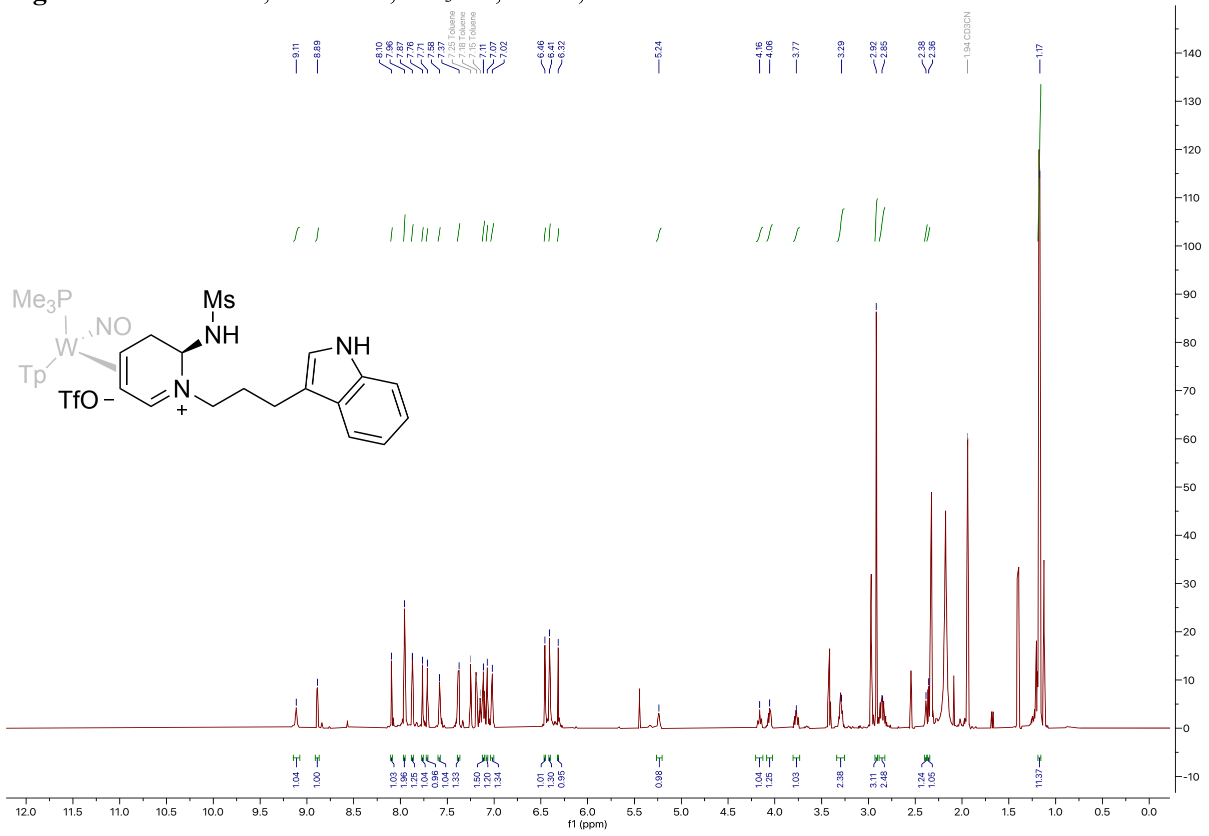

Figure S44: ^{13}C NMR, 200 MHz, CD_3CN, 25 °C, **6v**

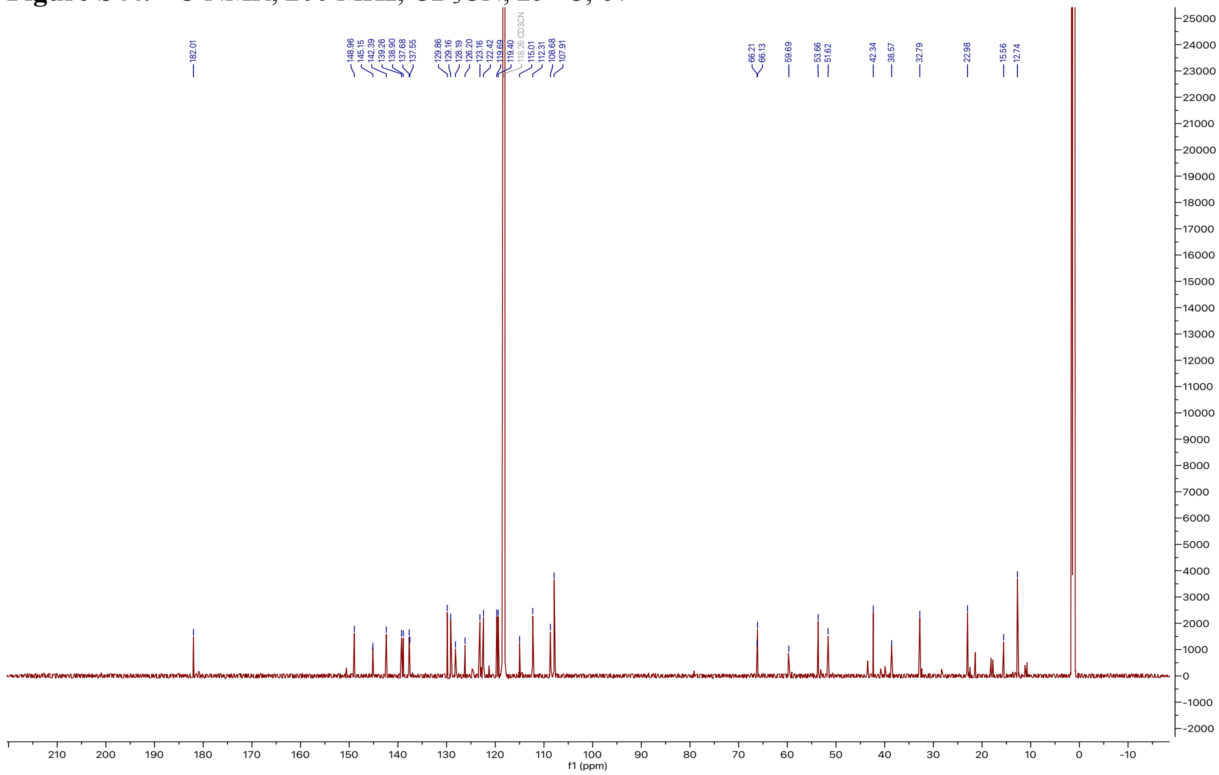

Figure S45: ^{1}H NMR, 800 MHz, CD_3CN, 25 °C, **7a**

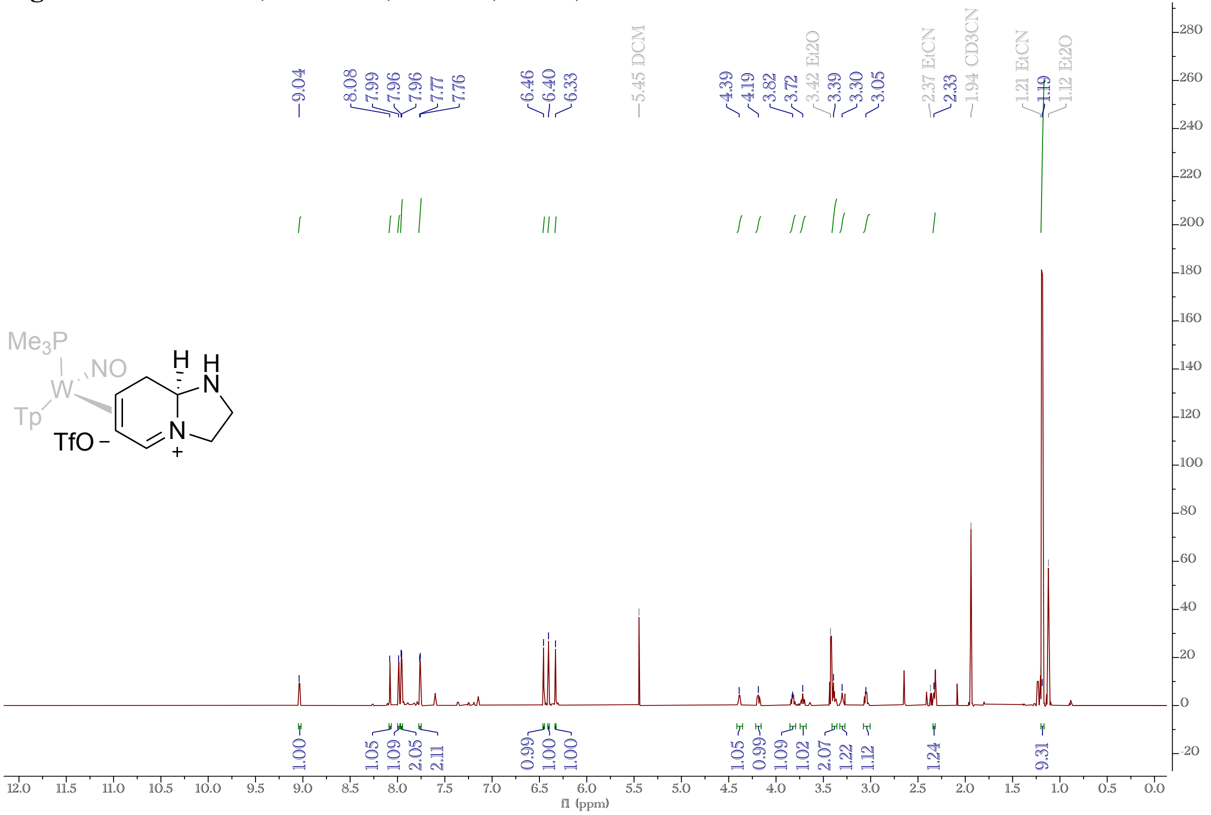

Figure S46: ^{13}C NMR, 200 MHz, CD_3CN, 25 °C, **7a**

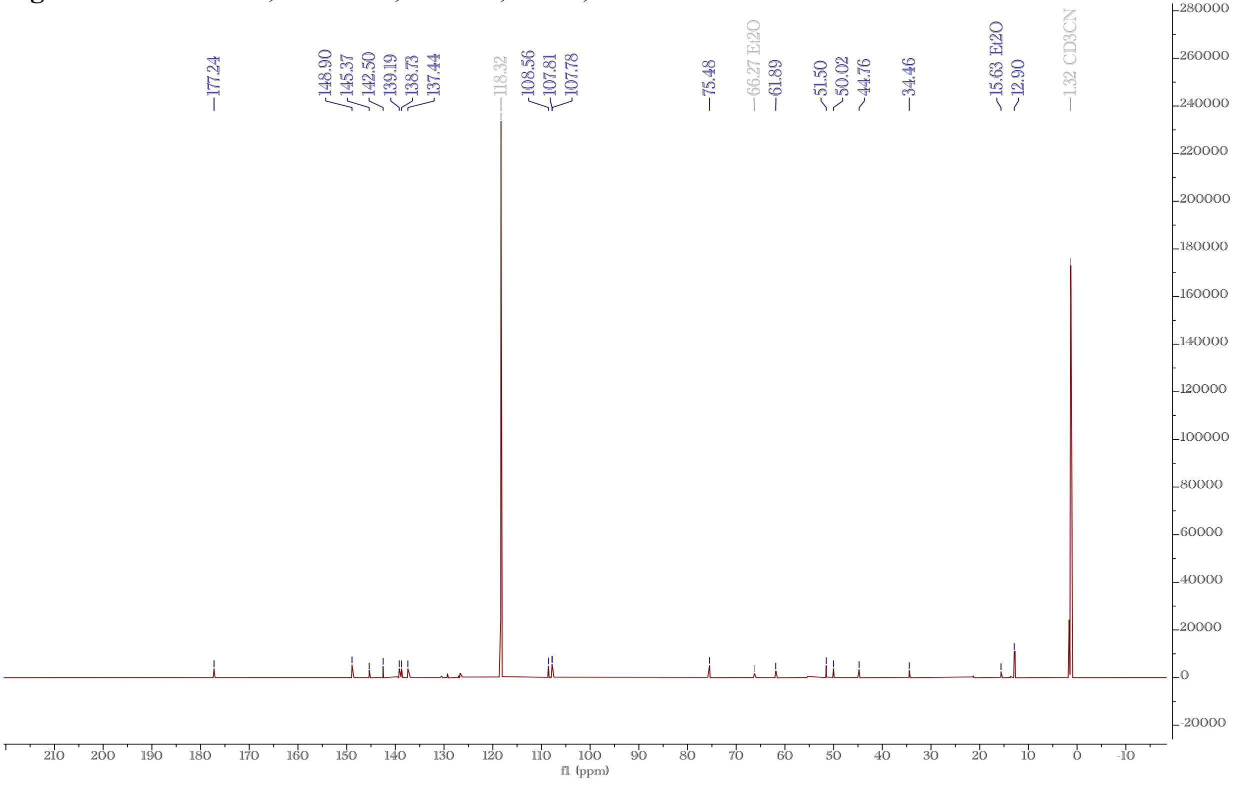

Figure S47: ^{1}H NMR, 800 MHz, CD_3CN, 25 °C, **7b**

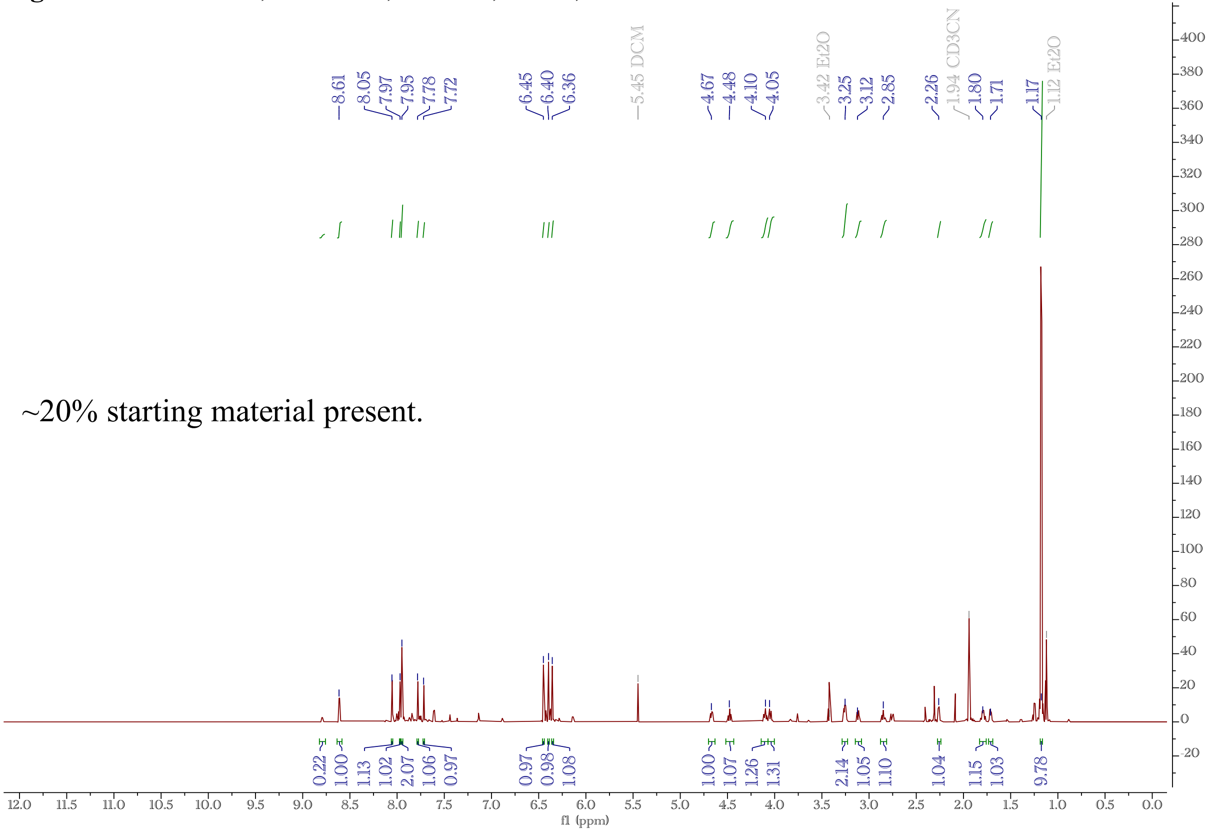

Figure S48: ^{13}C NMR, 200 MHz, CD_3CN, 25 °C, **7b**

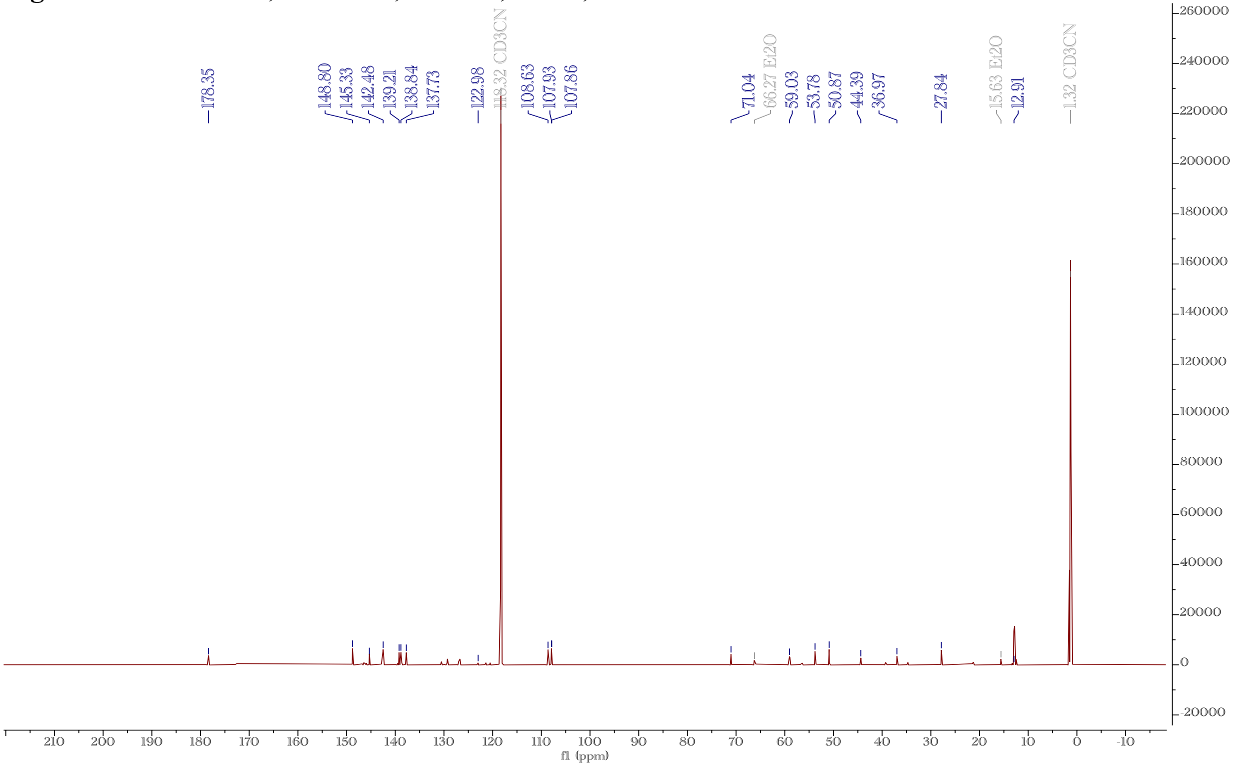

Figure S49: ^{1}H NMR, 800 MHz, CD_3CN, 25 °C, **7c**

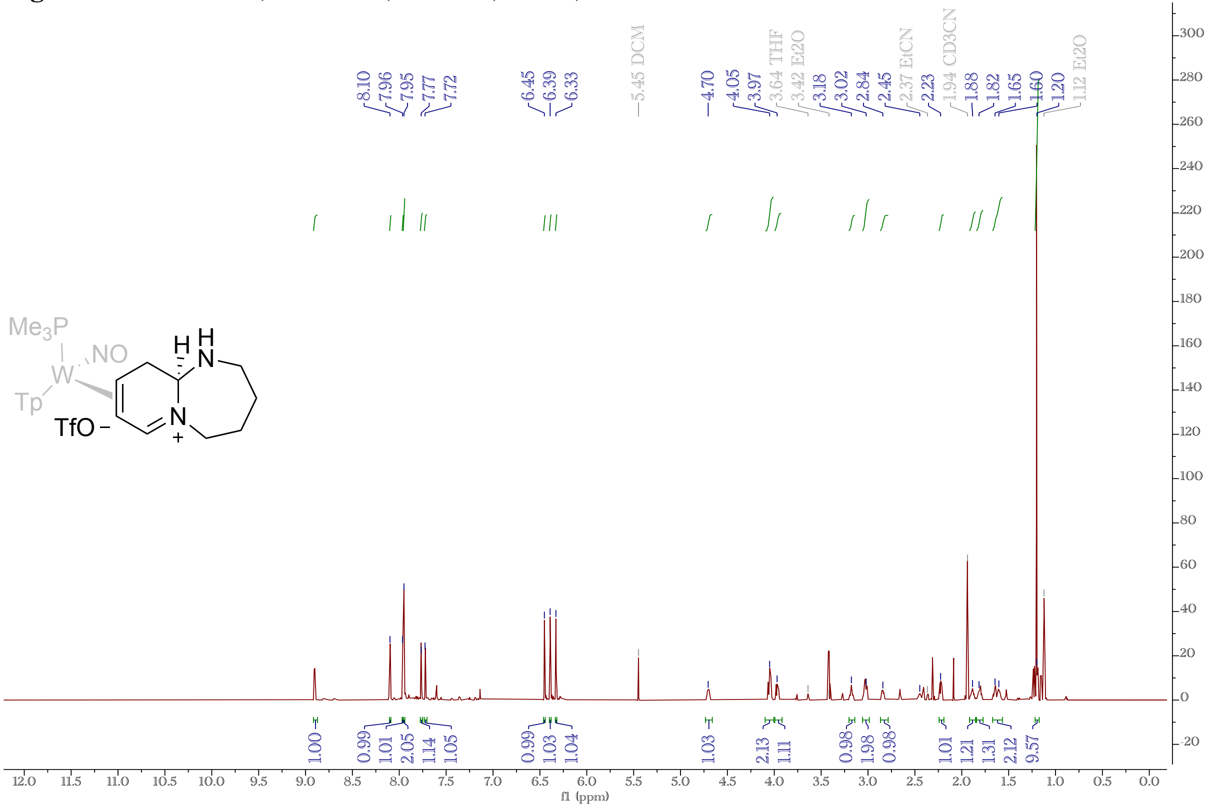

Figure S50: ^{13}C NMR, 200 MHz, CD_3CN, 25 °C, **7c**

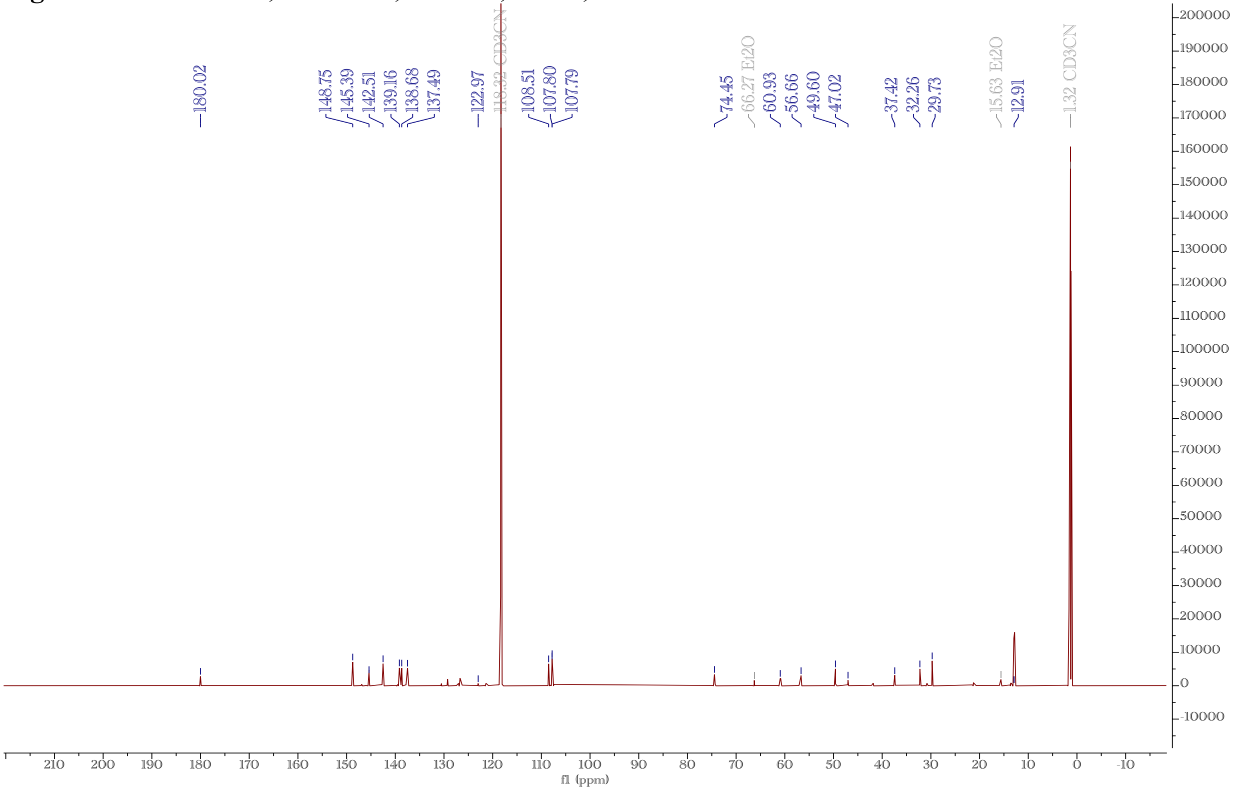

Figure S51: ^{1}H NMR, 800 MHz, CD_3CN, 25 °C, **7d**

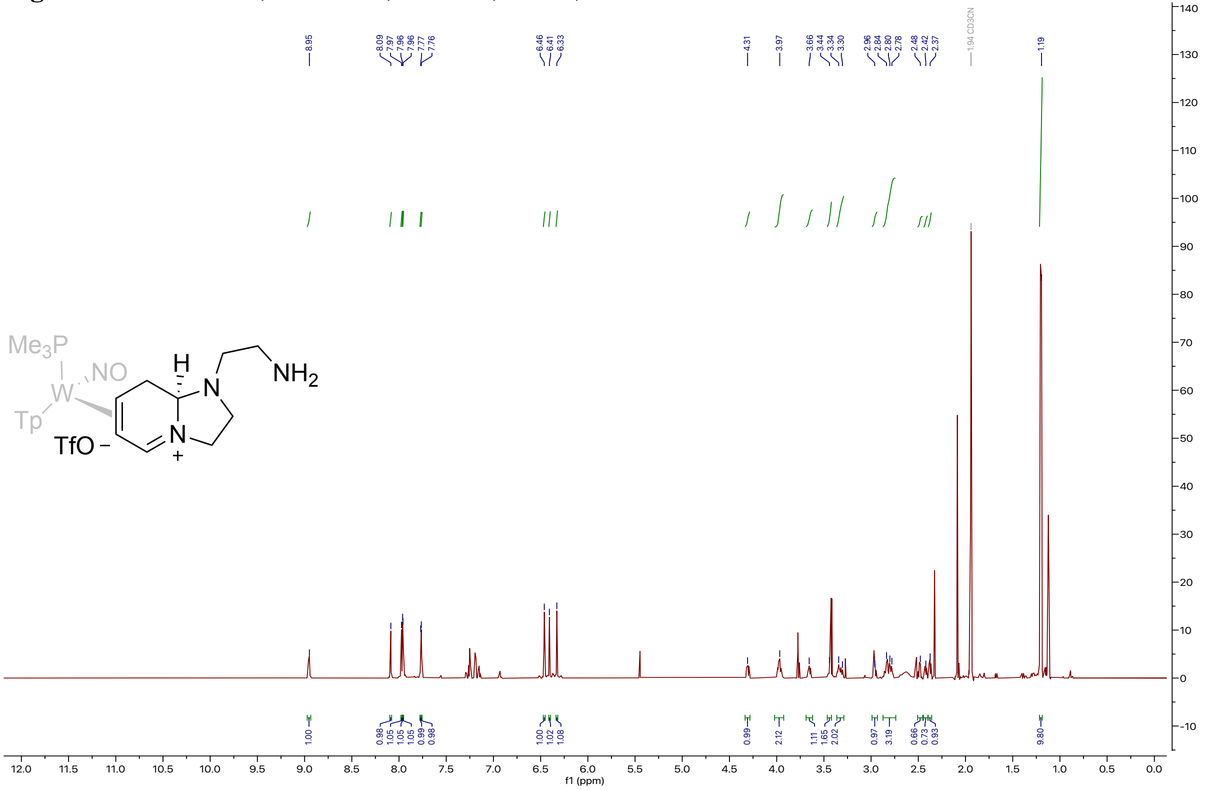

Figure S52: ^{13}C NMR, 200 MHz, CD_3CN, 25 °C, **7d**

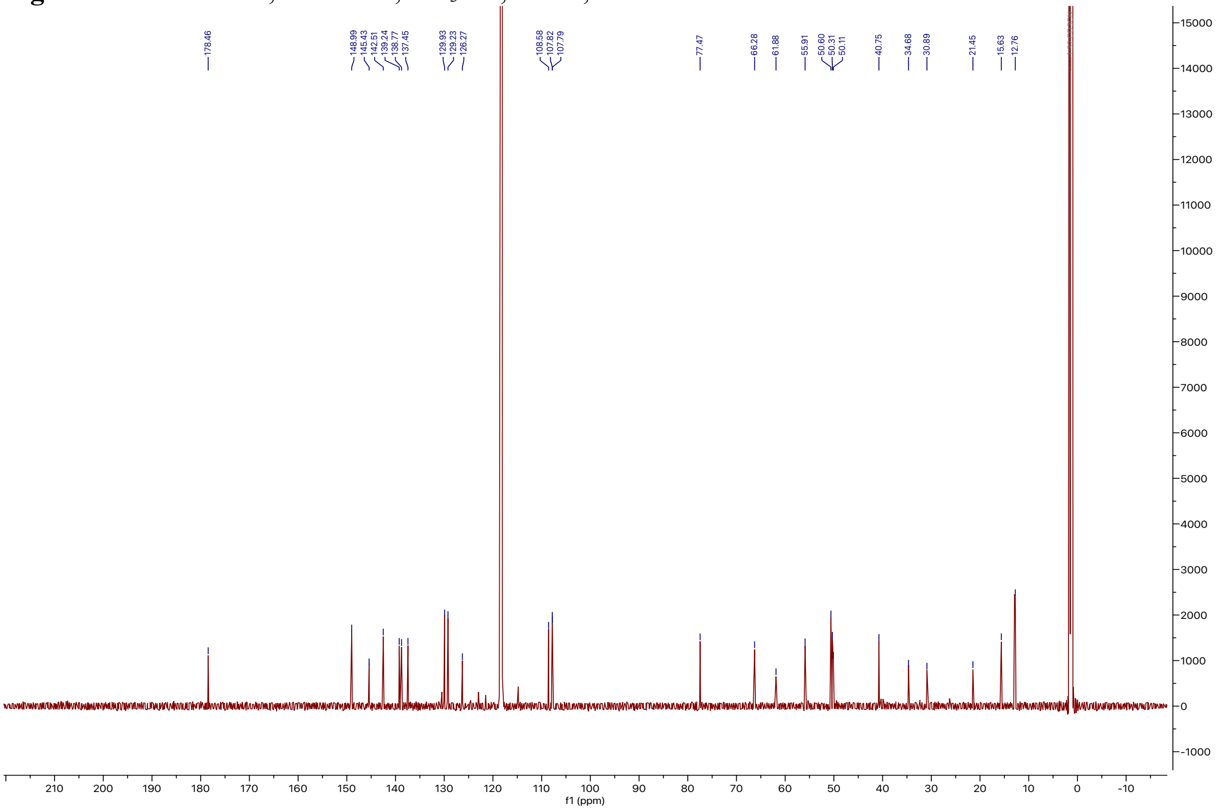

Figure S53: ^{1}H NMR, 800 MHz, CD_3CN, 25 °C, **7e**

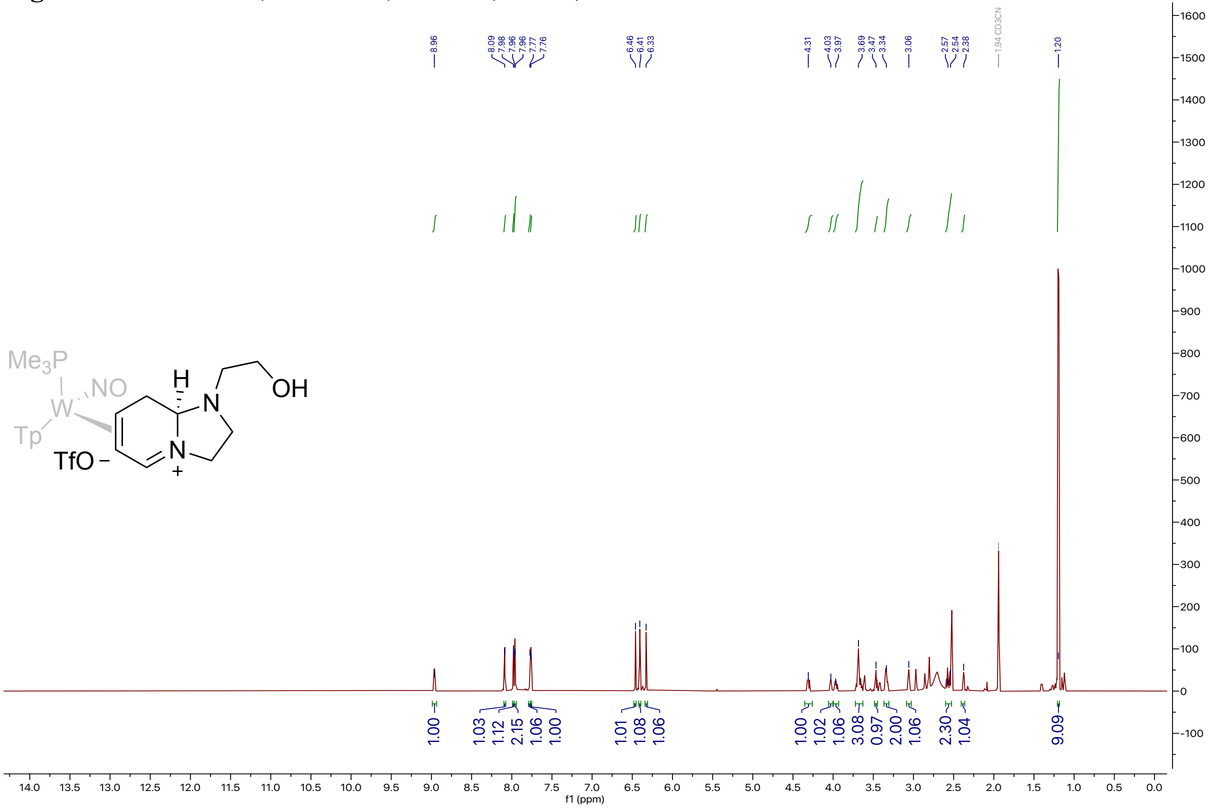

Figure S54: ^{13}C NMR, 200 MHz, CD_3CN, 25 °C, **7e**

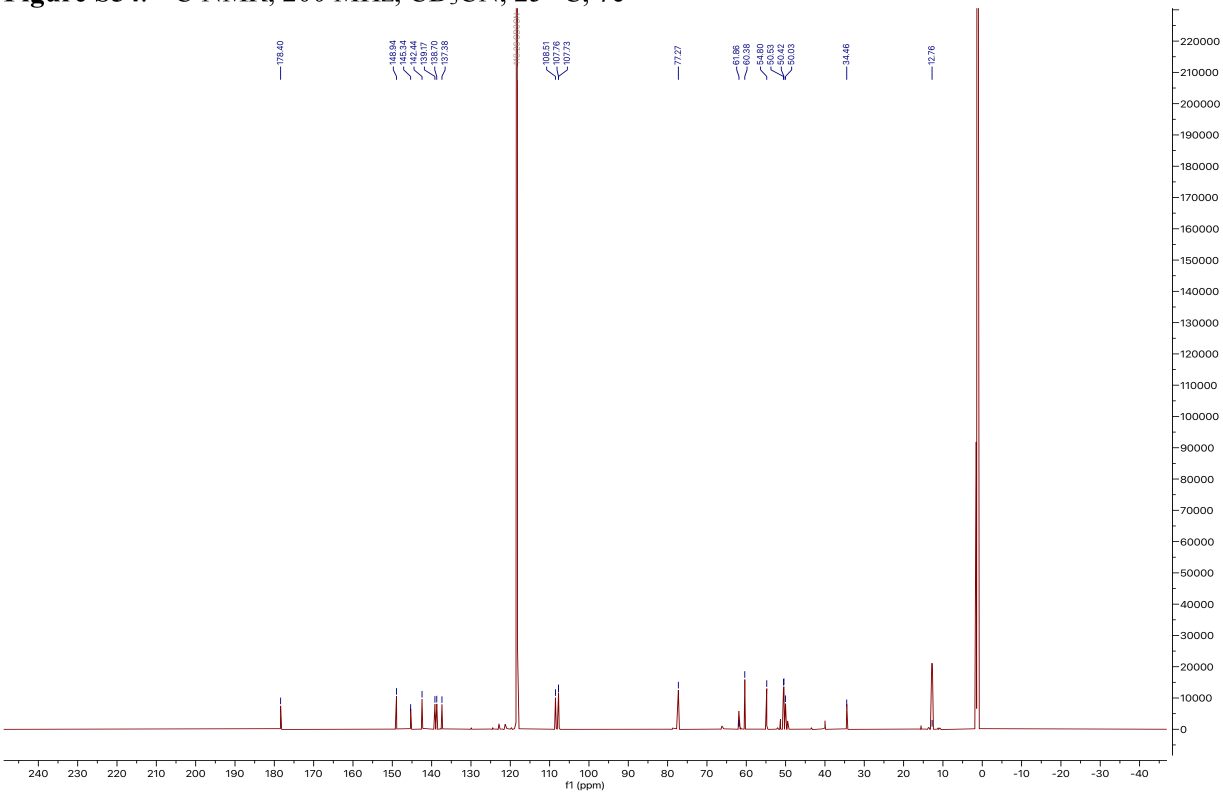

Figure S55: ^{1}H NMR, 800 MHz, CD_3CN, 25 °C, **7f**

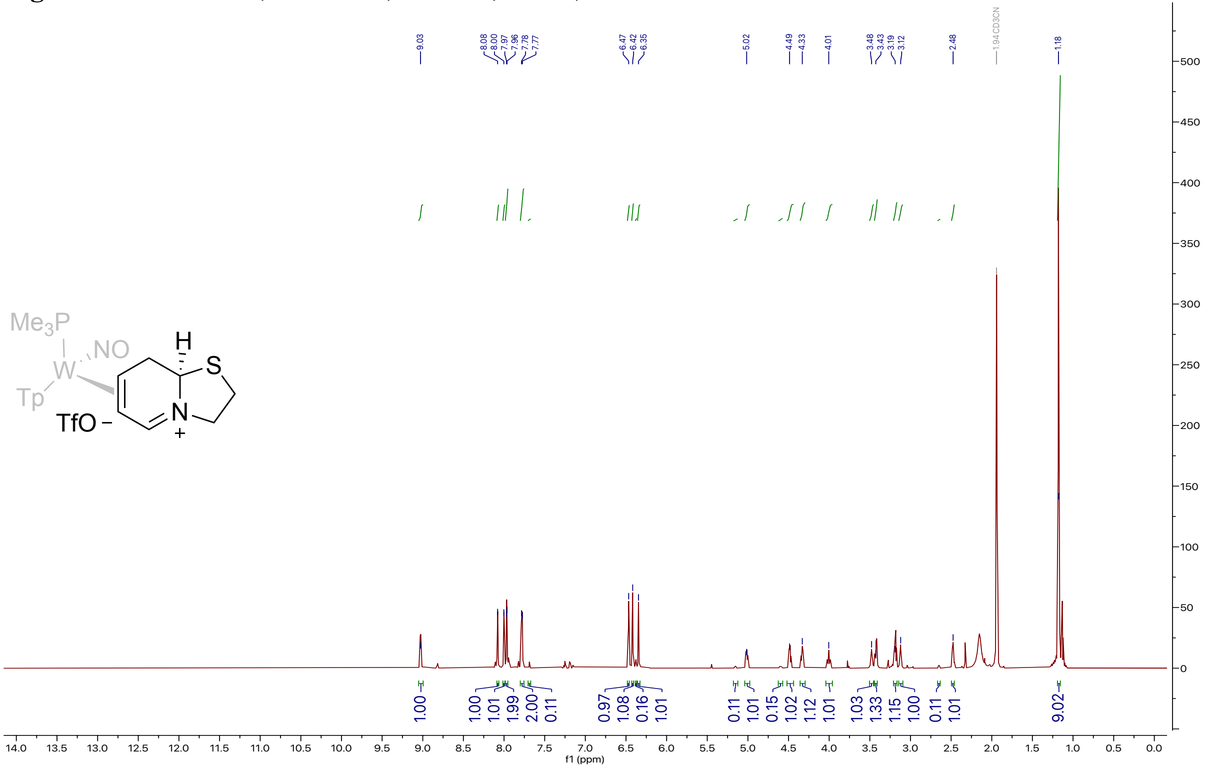

Figure S56: ^{13}C NMR, 200 MHz, CD_3CN, 25 °C, **7f**

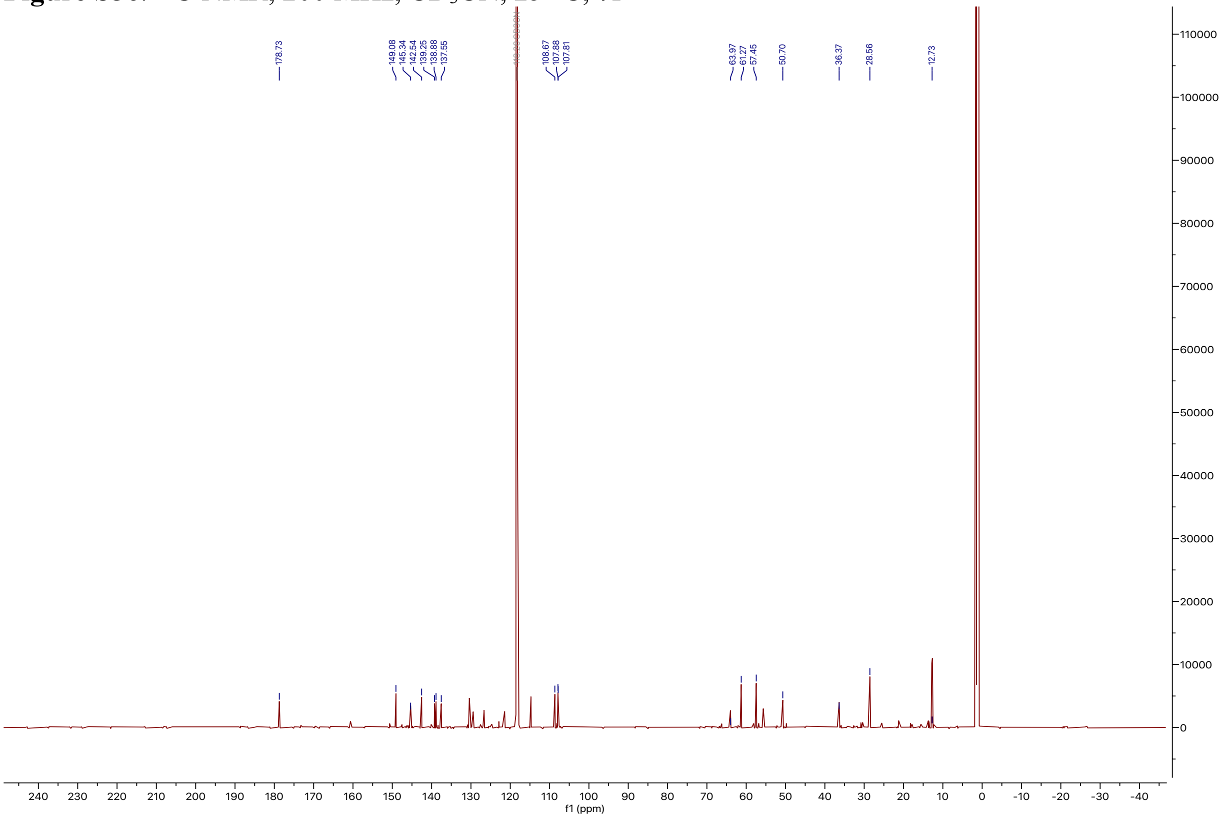

Figure S57: ^{1}H NMR, 800 MHz, CD_3CN, 25 °C, **7g**

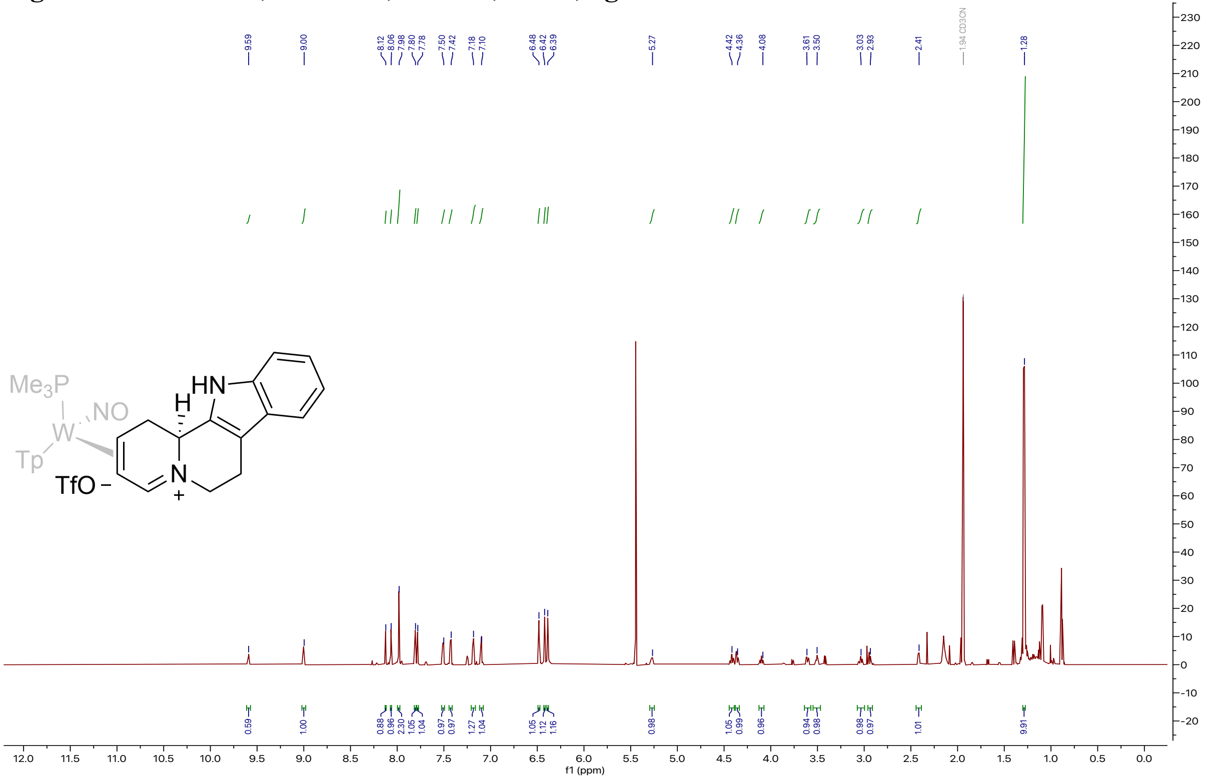

Figure S58: ^{13}C NMR, 200 MHz, CD_3CN, 25 °C, **7h**

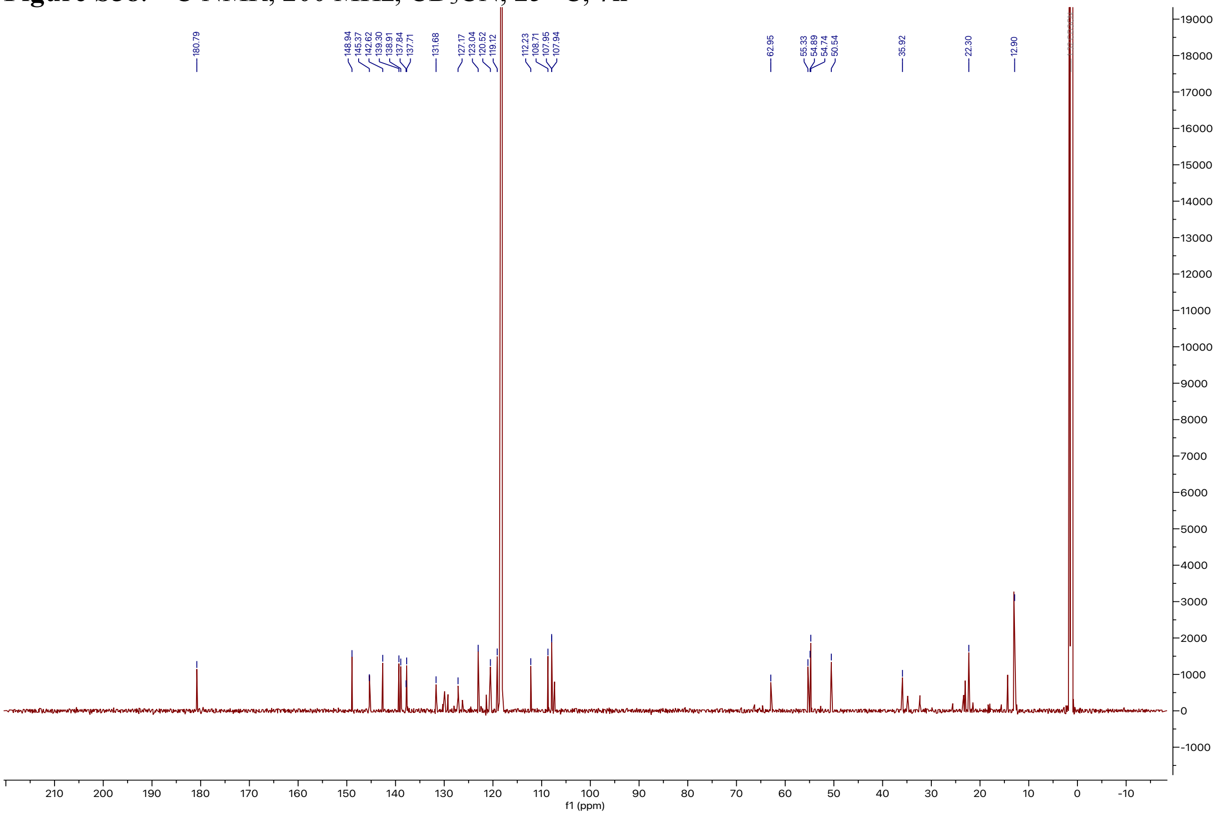

Figure S59: ^{1}H NMR, 800 MHz, CD_3CN, 25 °C, **8**

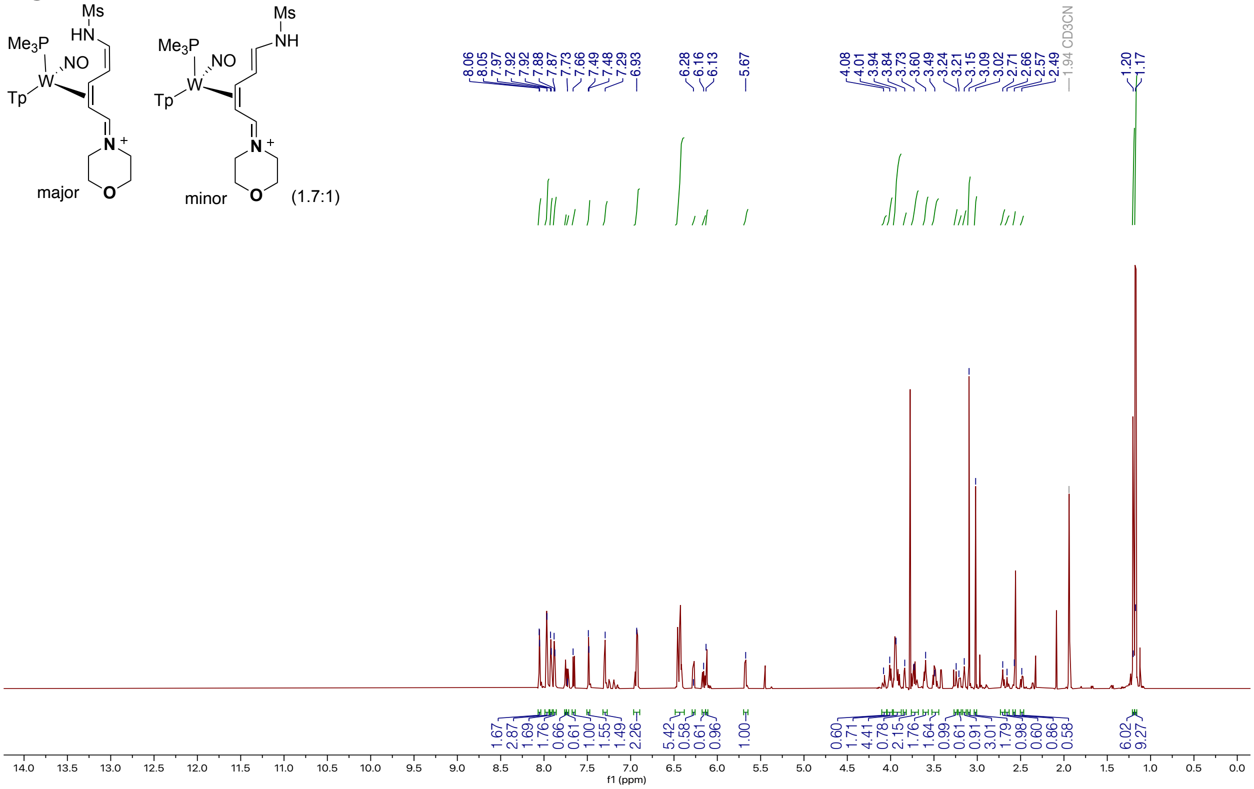

Figure S60: ^{13}C NMR, 200 MHz, CD_3CN, 25 °C, **8**

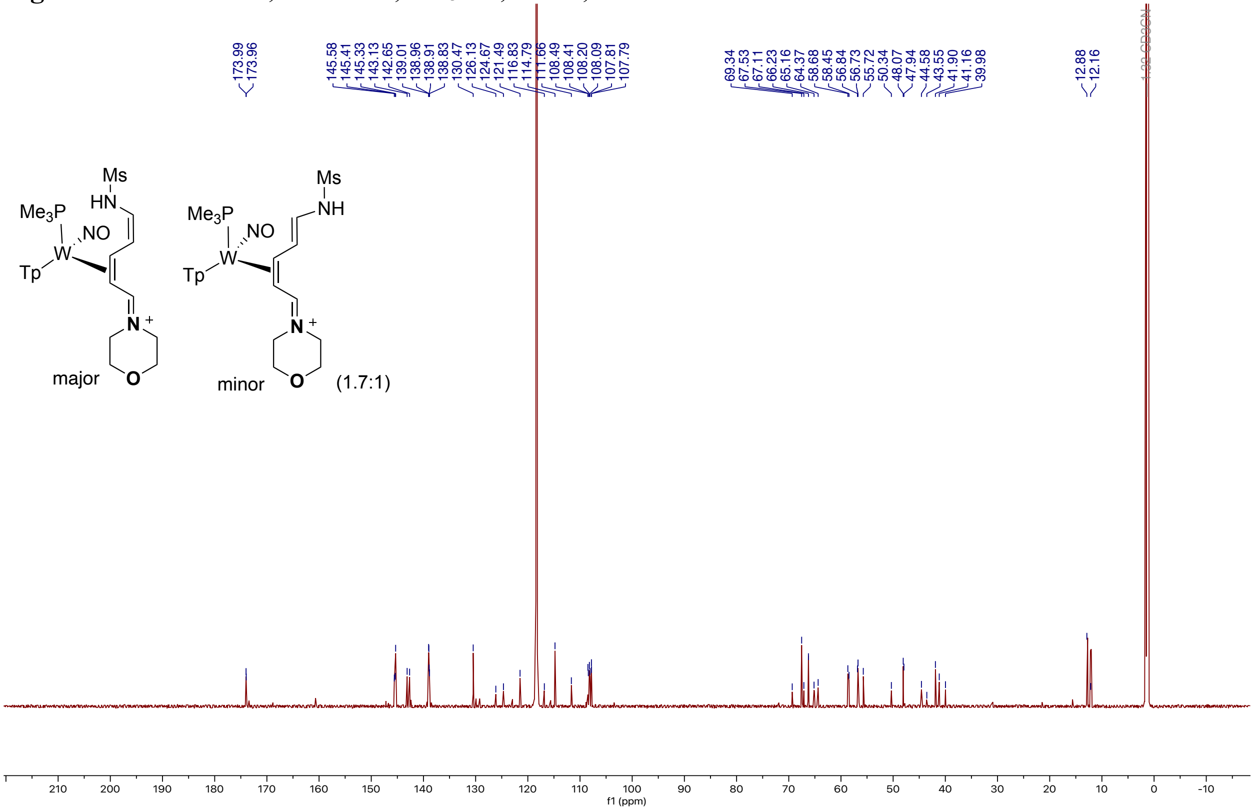

Figure S61: ^{1}H NMR, 800 MHz, CD_3CN, 25 °C, **9**

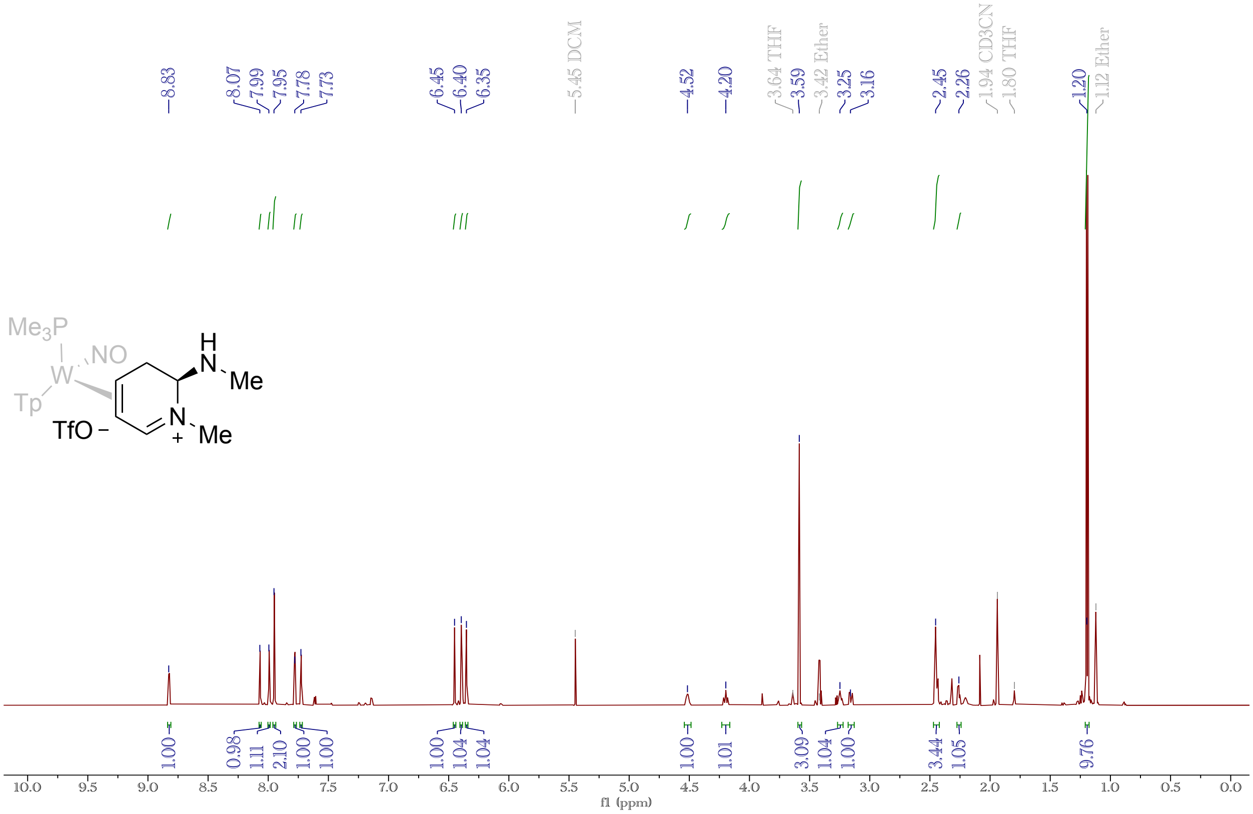

Figure S62: ^{13}C NMR, 200 MHz, CD_3CN, 25 °C, **9**

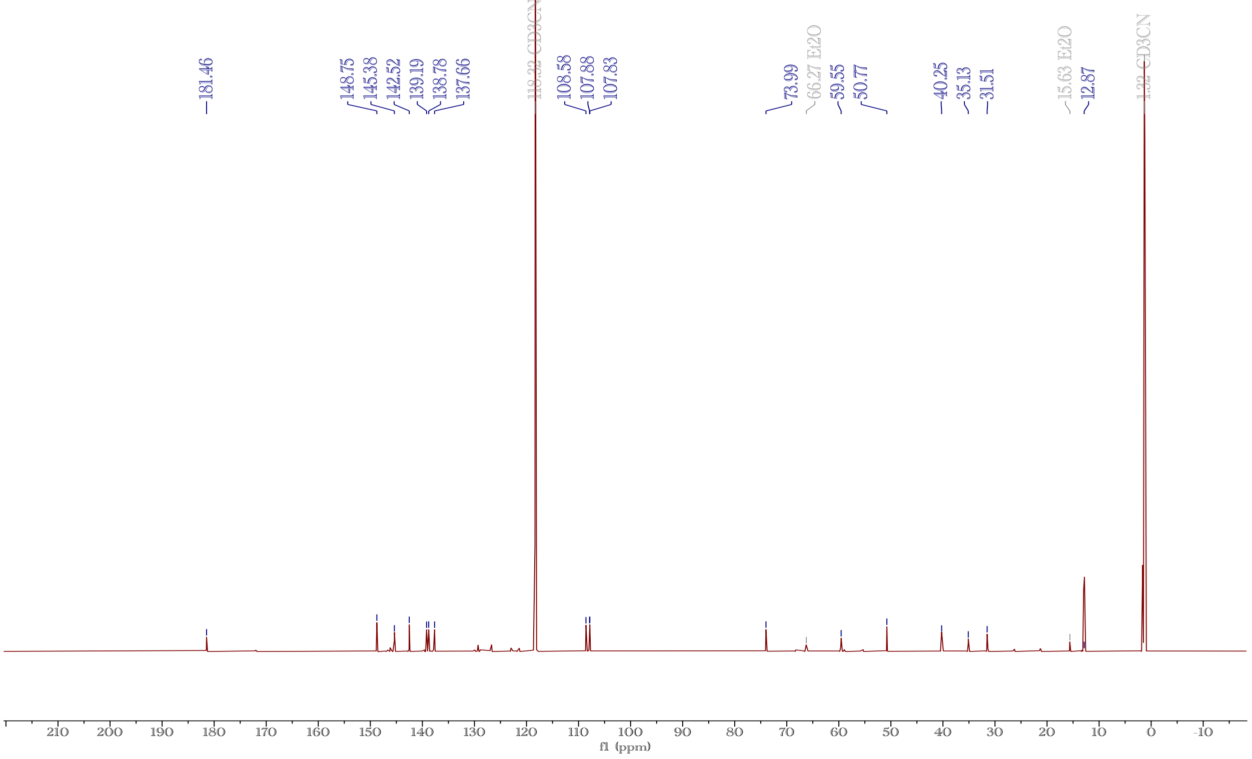

Figure S63: ^{1}H NMR, 800 MHz, CD_3CN, 25 °C, **10**

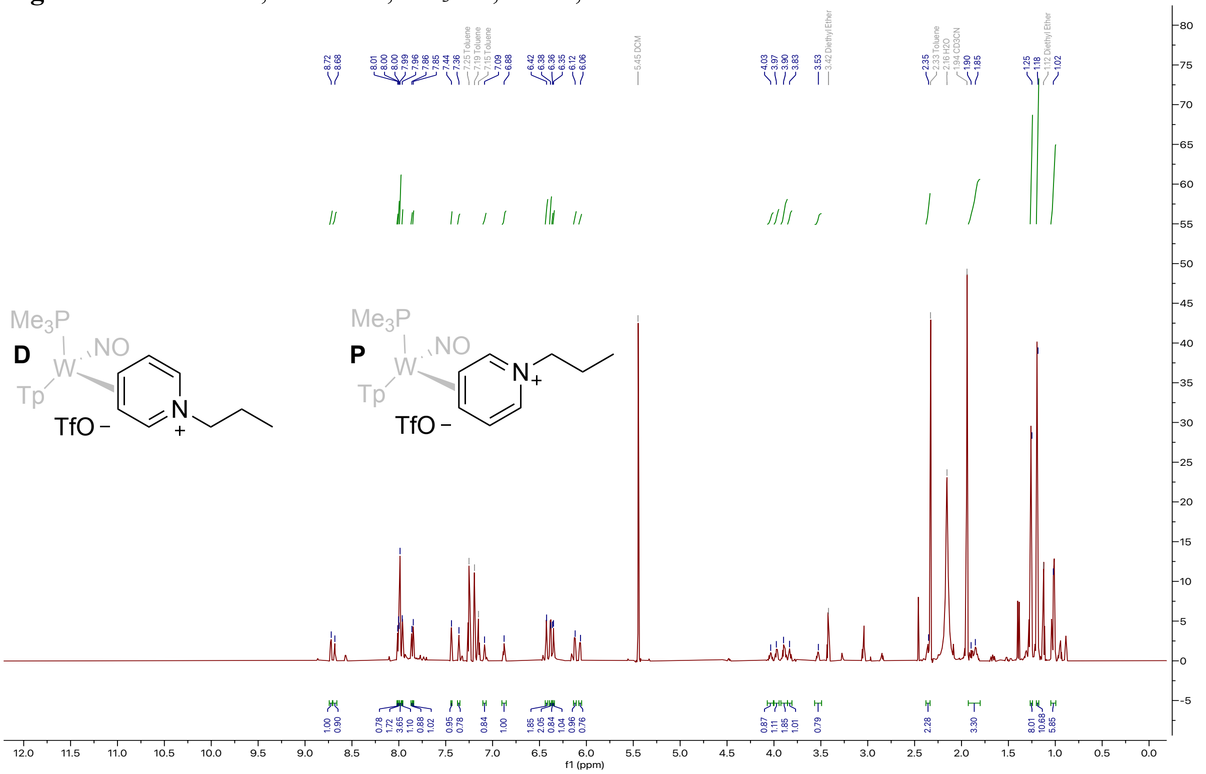

Figure S64: ^{13}C NMR, 200 MHz, CD_3CN, 25 °C, **10**

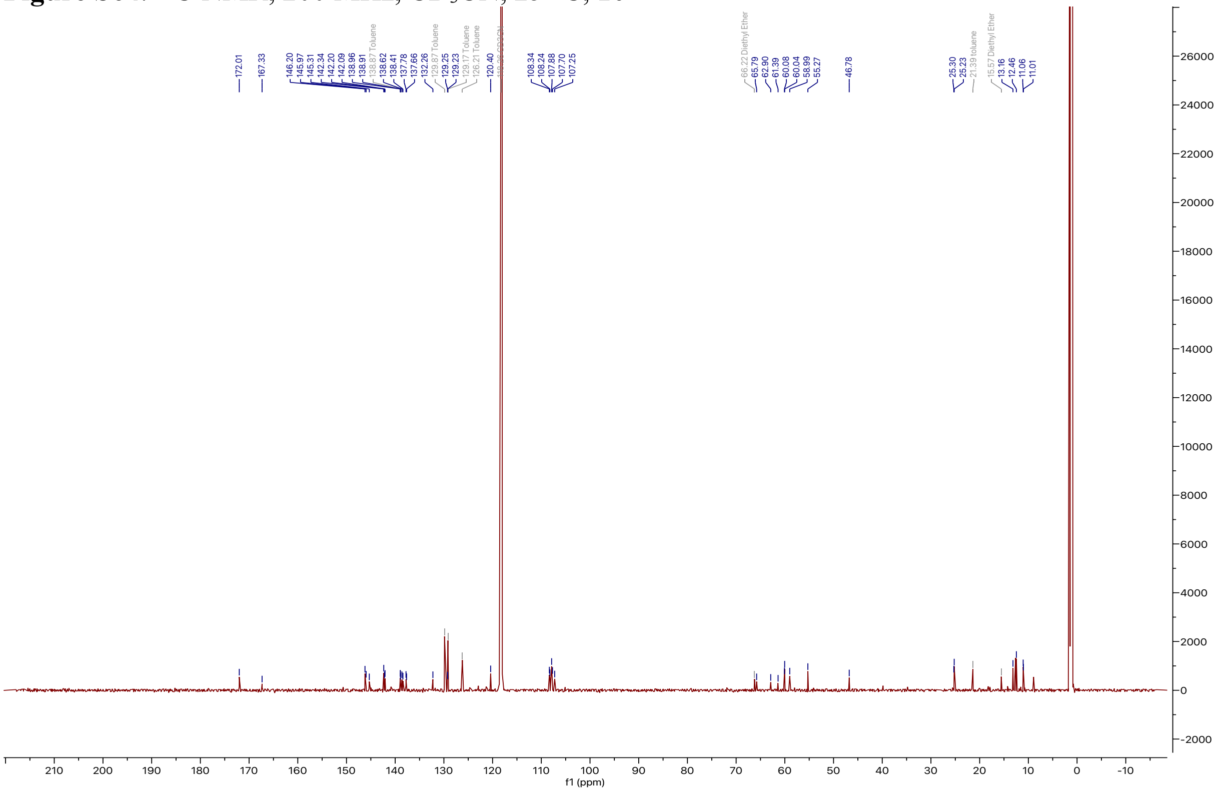

SC-XRD Data:

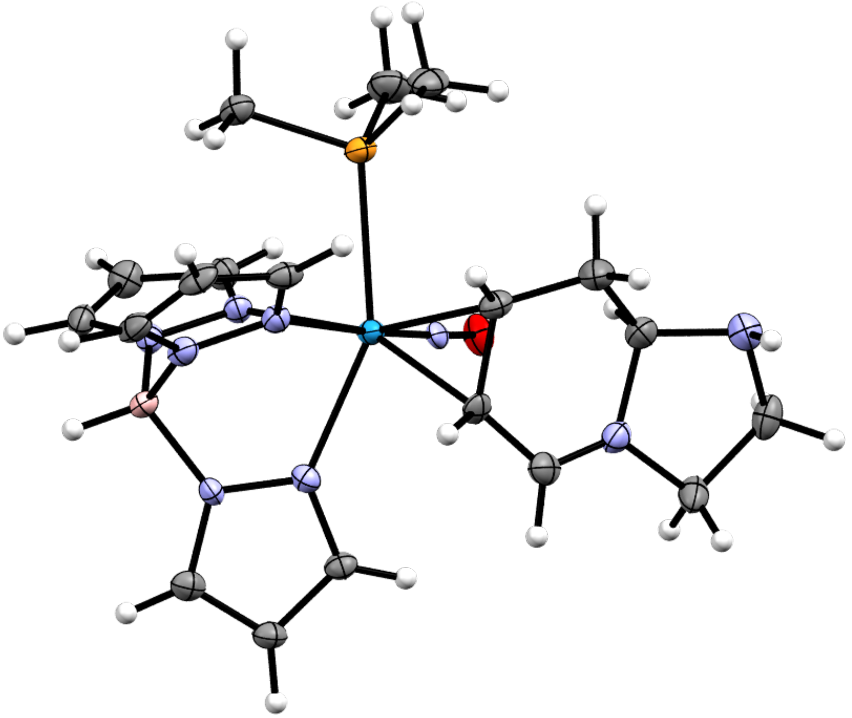

Figure S65: ORTEP/ellipsoid diagram of **7a** (disordered triflate omitted). R-enantiomer of the [W] fragment shown.

Table S1: SC-XRD data for **7a**.

CCDC	Chemical Formula	FW (g/mol)
2429406	$C_{20}H_{30}BF_3N_9O_4PSW$	775.22
T (K)	λ (Å)	Crystal size (mm)
100(2)	0.71073	0.046 x 0.067 x 0.090
Crystal habit	Crystal system	Space group
Yellow needle	Monoclinic	$P\ 2_1/n$
a (Å)	b (Å)	c (Å)
16.1373(6)	10.2833(3)	16.6623(6)
α (°)	β (°)	γ (°)
90	100.0990(10)	90
V (Å^3)	Z	ρ_{calc} (g/cm^3)
2722.18(16)	4	1.892
μ (mm^{-1})	F(000)	θ range (°)
4.446	1528	2.34 to 28.28
Index ranges	Data/restraints/parameters	Goodness-of-fit on F^2
$-21 \leq h \leq 21$<br>$-13 \leq k \leq 12$<br>$-22 \leq l \leq 19$	6757 / 30 / 394	1.009
R_1 [I>2σ(I)]	wR_2 [all data]	
0.0342	0.0717	

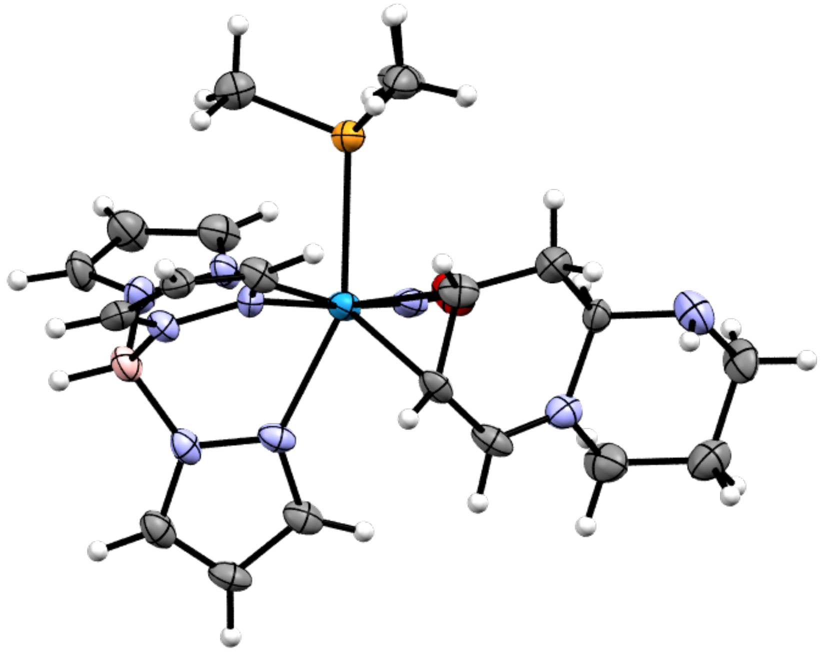

Figure S66: ORTEP/ellipsoid diagram of **7b** (triflate omitted). R-enantiomer of the [W] fragment shown.

Table S2: SC-XRD data for **7b**.

CCDC	Chemical Formula	FW (g/mol)
2429407	$C_{21}H_{32}BF_3N_9O_4PSW$	789.24
T (K)	λ (Å)	Crystal size (mm)
100(2)	0.71073	0.051 x 0.059 x 0.114
Crystal habit	Crystal system	Space group
Orange block	Monoclinic	$P\ 2_1/n$
a (Å)	b (Å)	c (Å)
15.8033(7)	10.6246(4)	17.4770(9)
α (°)	β (°)	γ (°)
90	98.4000(10)	90
V (Å^3)	Z	ρ_{calc} (g/cm^3)
2903.0(2)	4	1.806
μ (mm^{-1})	F(000)	θ range (°)
4.171	1560	2.25 to 27.49
Index ranges	Data/restraints/parameters	Goodness-of-fit on F^2
$-20 \leq h \leq 18$<br>$-12 \leq k \leq 13$<br>$-22 \leq l \leq 18$	6664 / 0 / 385	1.016
R_1 [I>2σ(I)]	wR_2 [all data]	
0.0557	0.1314	

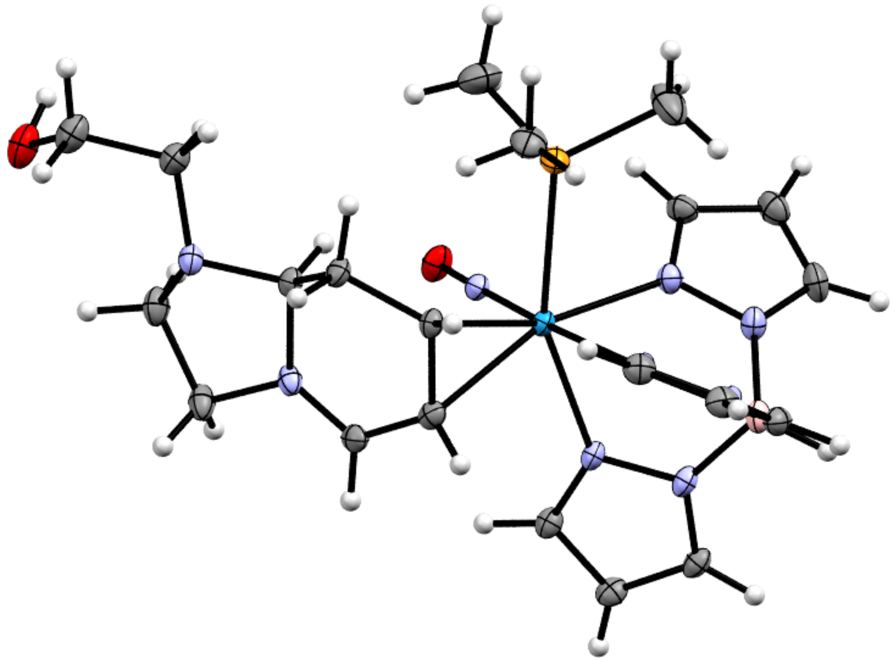

Figure S67: ORTEP/ellipsoid diagram of **7e** (triflate omitted). S-enantiomer of the [W] fragment shown.

Table S3: SC-XRD data for **7e.**

CCDC<br>2429408	Chemical Formula<br>$C_{22}H_{34}BF_3N_9O_5PSW$	FW (g/mol)<br>819.27
T (K)<br>100(2)	λ (Å)<br>0.71073	Crystal size (mm)<br>0.038 x 0.092 x 0.244
Crystal habit<br>Yellow needle	Crystal system<br>Triclinic	Space group<br>(2)
a (Å)<br>7.9016(4)	b (Å)<br>13.7411(6)	c (Å)<br>14.5890(7)
α (°)<br>106.9650(10)	β (°)<br>93.243(2)	γ (°)<br>94.967(12)
V (Å^3)<br>1503.83(12)	Z<br>2	ρ_{calc} (g/cm^3)<br>1.809
μ (mm^{-1})<br>4.032	F(000)<br>812	θ range (°)<br>3.58 to 59.16 (0.72 Å)
Index ranges<br>$-10 \leq h \leq 10$<br>$-19 \leq k \leq 16$<br>$-20 \leq l \leq 20$	Data/restraints/parameters<br>65224 / 0 / 407	Goodness-of-fit on F^2<br>1.029
R_1 [I>2σ(I)]<br>0.0236	wR_2 [all data]<br>0.0561	

Nitrogen Substitution Optimizations

Optimizations of the syntheses of **6a-6v** resulted in the observation of various side products (Figure S71). These impurities were all off shoots of the mechanism for synthesizing the **Target** compound. The **RO (M)** impurity is an incomplete **Target** pathway wherein the desired amine adds and triggers an initial ring-opening process; however, the subsequent ring-closing has not yet occurred. An alkene diastereomer **RO (m)** is also observed via this pathway. The **Ald** impurity forms from the addition of hydroxide from residual water in the presence of amine base resulting in an aldehyde ring-opening impurity identical to **RO (M)** (an alkene diastereomer is not observed with this impurity). When the **Target** is successfully synthesized, rearomatization can occur when the sulfonamide undergoes an elimination with an adjacent proton. This forms **ReAr (M)**, but proximal/distal isomerization on the [W] fragment occurs rapidly upon rearomatization, resulting in **ReAr (m)**. Similarly, the [W] can briefly support an η^2-bound dicationic ligand. A weak acid source can promote the protonation and facile elimination of the sulfonamide. The resulting highly electrophilic ligand can undergo a second nucleophilic addition of excess residual amine, forming the **Double** substitution product. Generally, colder temperatures, fewer equivalencies, and shorter reaction times result in higher levels of **RO (M)** and **RO (m)** impurities while warmer temperatures, longer reaction times, and higher equivalencies results in increased **ReAr (M)**, **ReAr (m)**, and **Double** impurity formation.

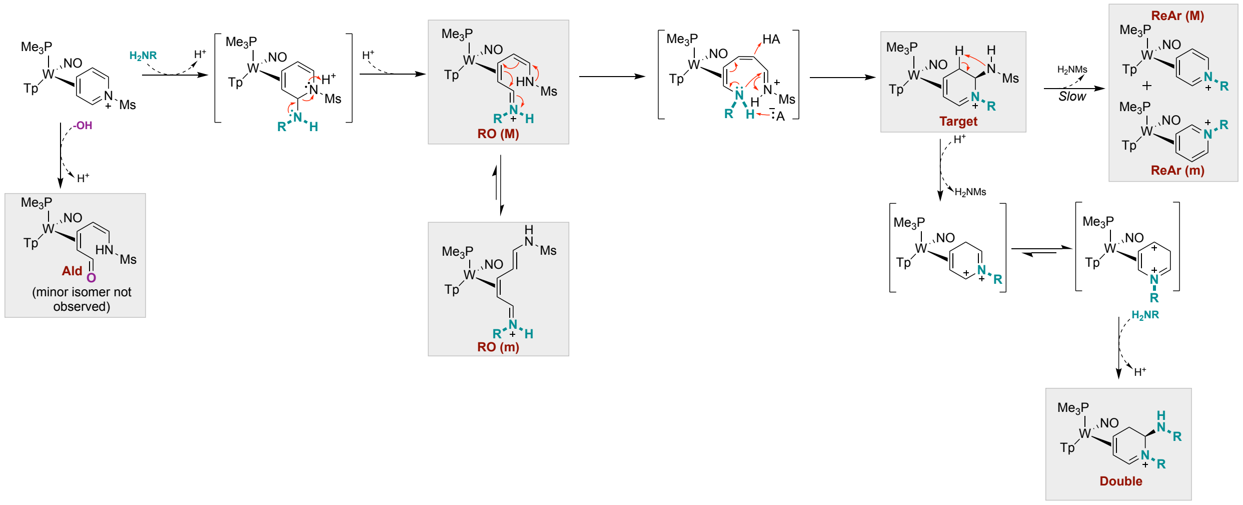

Figure S68: Mechanism pathway for the **Target** and each impurity observed in the optimizations of **6a-6v**.

Table S4: A variety of conditions screened and the resulting product ratios for **6a-6e**.

Cmpd. (Eq.)	°C	min.	Solvent(s)	Target	Double	ReAr (M)	ReAr (m)	RO (M)	RO (m)	Ald
**6a (2)**	-40	5	9 mL (DCM), 1 mL (EtCN)	1	0.15	0.06	0.03	0.17	0	0
**6a (2)**	-40	15	5 mL (DCM), 1.5 mL (EtCN)	1	0.09	0.08	0	0.89	(overlap)	0.2
**6a (2)**	-30	5	2 mL (DCM), 2 mL (EtCN)	1	0.08	0	0	2.71	(overlap)	0
**6a (2)**	-20	5	1 mL DCM-d2	1	0.89	0.31	0	0	0	0
**6a (4)**	-40	15	8 mL (DCM), 2 mL (EtCN)	1	0.08	0.03	0.02	0	0	0
**6a (4)**	-40	15	9 mL (DCM), 3 mL (EtCN)	1	0.21	0.05	0.02	0.78	(overlap)	0.04
**6a (4)**	-20	5	1 mL (DCM), 1 mL (EtCN)	1	0.55	0	0	1.83	(overlap)	0
**6a (4)**	20	5	2 mL (DCM), 2 mL (EtCN)	1	0.17	0	0	0	0	0
**6a (5)**	-20	5	2 mL (DCM), 2 mL (EtCN)	1	0	0	0	0.27	0	0
**6a (5)**	-20	5	2 mL (DCM), 2 mL (EtCN)	1	0.22	0	0	1.46	(overlap)	0
**6a (5)**	-20	60	1 mL (DCM), 1 mL (EtCN)	0.86	1	0	0	1.15	(overlap)	0
**6a (5)**	-20	60	2 mL (DCM), 2 mL (EtCN)	0.12	1	0	0	0	0	0
**6a (6)**	20	5	1 mL (DCM)	0	1	0	0	0	0	0
**6a (8)**	20	5	1 mL (DCM), 1 mL (MeCN)	1	0	0.05	0	0	0	0
**6a (11)**	-20	5	2 mL (DCM), 2 mL (EtCN)	1	0.93	0	0	0	0	0
**6b (1.1)**	-30	15	2 mL (DCM), 1 mL (EtCN)	1	0.08	0.06	0.03	0.37	(overlap)	0.02
**6b (1.5)**	-30	15	2 mL (DCM), 1 mL (EtCN)	1	0.04	0	0	0	0	0
**6b (2)**	-40	15	2 mL (DCM). 0.5 mL (EtCN)	1	0.03	0.05	0.05	0.68	(overlap)	0.02
**6b (2)**	-40	15	5 mL (DCM), 1 mL (EtCN)	1	0	0	0	0	0	0
**6b (2)**	-40	15	12 mL (DCM)	1	0.1	0.1	0.08	0.27	(overlap)	0
**6b (2.5)**	-30	15	10 mL (DCM), 1 mL (EtCN)	1	0.12	0.03	0.02	0	0	0
**6b (3.5)**	-30	15	8 mL (DCM), 4 mL (EtCN)	1	0.1	0.03	0.02	0	0	0
**6b (11)**	-20	5	2 mL (DCM), 2 mL (EtCN)	1	0	0	0	0	0	0
**6c (2)**	-30	15	8 mL (DCM), 4 mL (EtCN)	1	0.04	0.02	0	0.13	(overlap)	0.02
**6d (9)**	20	20	0.5 mL d3-MeCN	0	1	0.26	0.05	0	0	0
**6d (9)**	20	40	0.5 mL d3-MeCN	0	1	0.33	0.06	0	0	0
**6d (9)**	20	60	0.5 mL d3-MeCN	0	1	0.59	0.08	0	0	0
**6e (1.5)**	20	5	2 mL (DCM), 1 mL (EtCN)	1	0.23	0.09	0.06	0.12	(overlap)	0
**6e (2)**	-40	5	8 mL (DCM), 4 mL (EtCN)	1	0.45	0	0	1.54	(overlap)	0

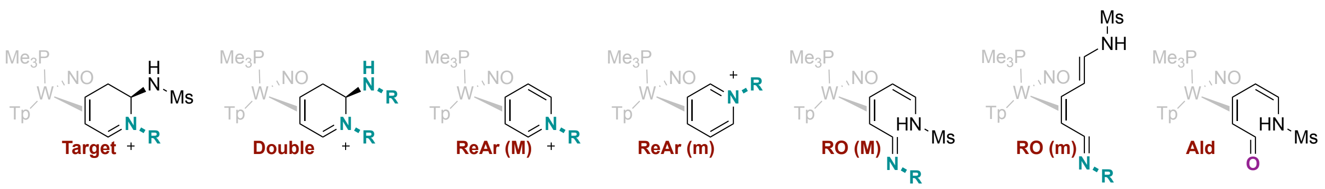

Table S5: A variety of conditions screened and the resulting product ratios for **6g-6p**.

Cmpd. (Eq.)	°C	min.	Solvent(s)	Target	Double	ReAr (M)	ReAr (m)	RO (M)	RO (m)	Ald
**6g (2)**	-40	15	8 mL (DCM), 2 mL (EtCN)	1	0.02	0.02	0.01	0	0	0
**6g (2)**	20	5	2 mL (DCM), 1 mL (EtCN)	1	0.15	0.07	0.03	0	0	0
**6g (3)**	-30	15	8 mL (DCM), 2mL (EtCN)	1	0	0.02	0.02	0	0	0
**6g (5)**	-40	60	2 mL (DCM), 2 mL (EtCN)	1	0.82	0	0	0	0	0
**6g (5)**	-20	60	2 mL (DCM), 2 mL (EtCN)	0	1	0	0	0	0	0
**6h (1.5)**	20	5	2 mL (DCM), 1 mL (EtCN)	1	0	0.19	0	0	0	0
**6h (2)**	-30	15	8 mL (DCM), 4 mL (EtCN)	1	0.03	0.08	0	0	0	0
**6h (3)**	-40	60	2 mL (DCM), 2 mL (EtCN)	1	0	0	0	0	0	0
**6h (3)**	-20	60	2 mL (DCM), 2 mL (EtCN)	1	0.48	0.18	0.09	0	0	0
**6i (4)**	-40	15	8 mL (DCM), 4 mL (EtCN)	1	0.03	0.03	0.02	0.63	0.2	0.04
**6i (4)**	-30	15	8 mL (DCM), 4 mL (EtCN)	1	0.16	0.13	0.02	0.43	0.12	0.07
**6k (1.5)**	20	5	2 mL (DCM), 1 mL (MeCN)	1	0	0.03	0.03	0	0	0
**6k (2)**	-40	5	8 mL (DCM), 4 mL (EtCN)	1	0	0	0	0	0	0
**6k (2)**	-30	15	9 mL (DCM), 3 mL (EtCN)	1	0	0	0	0.31	(overlap)	0
**6m (1.5)**	-40	15	8 mL (DCM), 3 mL (EtCN)	1	0.04	0.07	0.05	0	0	0
**6m (2)**	20	5	4 mL (DCM), 1 mL (MeCN)	1	0.12	0.03	0.02	0	0	0
**6m (14)**	-40	15	8 mL (DCM), 3 mL (EtCN)	1	0.22	0	0	0.73	(overlap)	0
**6n (2)**	-40	5	1.5 mL (DCM), 1.5 mL (EtCN)	1	0.27	0.29	0.21	4.12	(overlap)	0.33
**6n (2)**	-40	15	9 mL (DCM), 2 mL (EtCN)	1	0.06	0.03	0.02	0	0	0.03
**6n (2)**	-40	15	9 mL (DCM), 1 mL (EtCN)	1	0.07	0.05	0	0.57	0	0.07
**6n (2)**	-40	30	9 mL (DCM), 1 mL (EtCN)	1	0.1	0.09	0.05	0.38	(overlap)	0.04
**6n (2)**	-20	5	1.5 mL (DCM), 1.5 mL (EtCN)	1	0.12	0.1	0	1.45	(overlap)	0.12
**6o (2)**	-30	15	8 mL (DCM), 4 mL (EtCN)	1	0	0	0	0	0	0
**6p (1.5)**	-20	30	10 mL (DCM), 1 mL (EtCN)	1	0.08	0	0	0.28	0	0
**6p (2)**	-40	30	9 mL (DCM), 1 mL (EtCN)	1	0.06	0.03	0.03	0.12	0.08	0.02
**6p (5)**	-30	5	2 mL (DCM), 2 mL (EtCN)	1	0.15	0.07	0.03	0.29	0.18	0
**6p (5)**	-20	5	2 mL (DCM), 2 mL (EtCN)	1	0.12	0	0	0	0	0

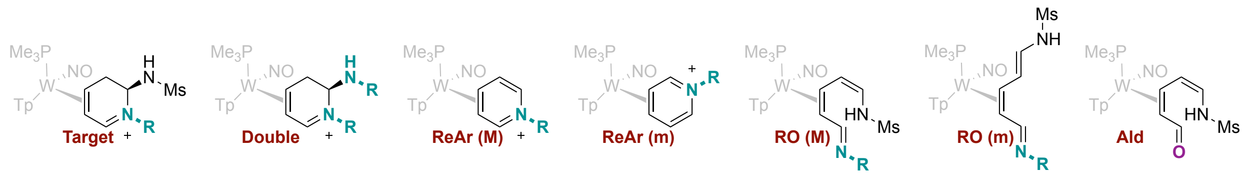

Table S6: A variety of conditions screened and the resulting product ratios for **6q-6u**.

Cmpd. (Eq.)	°C	min.	Solvent(s)	Target	Double	ReAr (M)	ReAr (m)	RO (M)	RO (m)	Ald
**6q (1.5)**	20	5	1 mL (DCM), 0.5 mL (MeCN)	1	0	0	0	0.08	0.04	0
**6q (3)**	-30	15	8 mL (DCM), 4 mL (EtCN)	1	0.04	0.12	0.02	0	0	0
**6r (1.5)**	20	5	1 mL (DCM), 0.5 mL (MeCN)	1	0.08	0.04	0.03	0	0	0
**6r (2)**	-40	5	8 mL (DCM), 4 mL (EtCN)	1	0.03	0.05	0.01	0.64	(overlap)	0.04
**6r (2)**	-40	15	2 mL (DCM)	1	0.19	0.32	0.08	0.88	(overlap)	0
**6r (2)**	-30	15	8 mL (DCM), 4 mL (EtCN)	1	0	0	0	0.45	(overlap)	0
**6r (2)**	-30	15	9 mL (DCM), 3 mL (EtCN)	1	0	0	0	0.23	(overlap)	0.03
**6r (3)**	-15	15	4 mL (DCM), 2 mL (MeCN)	1	0.22	0	0	0	0	0
**6s (1.5)**	-30	15	5 mL (DCM), 0.5 mL (EtCN)	1	0.04	0.05	0.02	0.43	0.33	0.09
**6s (2)**	-30	15	9 mL (DCM), 3 mL (EtCN)	1	0.06	0.02	0	0.15	0.11	0.03
**6s (3)**	-40	15	5 mL (DCM), 1 mL (EtCN)	1	0.09	0.06	0.05	0.29	0.22	0.07
**6t (1)**	-30	15	2 mL (DCM), 1 mL (EtCN)	1	0.06	0.07	0.03	0	0	0.09
**6t (1.5)**	-40	30	9 mL (DCM), 1 mL (EtCN)	1	0.1	0.06	0.03	0.23	0	0
**6t (1.5)**	-30	15	5 mL (DCM), 0.5 mL (EtCN)	1	0.1	0.1	0.05	0	0	0.04
**6t (1.5)**	-20	30	10 mL (DCM), 2 mL (EtCN)	1	0.07	0	0	0	0	0.13
**6t (1.5)**	20	5	0.5 mL DCM	1	0.35	0.03	0.03	0.17	0	0
**6t (2)**	-40	5	3 mL (DCM), 3 mL (EtCN)	1	0.18	0	0	0.76	0	0
**6t (2)**	-40	15	2 mL (DCM). 0.5 mL (EtCN)	1	0.03	0.07	0	0.06	0	0
**6t (2)**	-40	15	4.5 mL (DCM), 1 mL (EtCN)	1	0.16	0.03	0.01	0.12	0	0
**6t (2)**	-40	15	9 mL (DCM), 1 mL (EtCN)	1	0.05	0	0	0	0	0
**6t (2)**	-30	15	8 mL (DCM), 1 mL (EtCN)	1	0.1	0	0	0.29	0	0
**6t (2)**	-20	5	2 mL (DCM), 2 mL (EtCN)	1	0.22	0.04	0.03	0	0	0
**6t (3)**	-40	15	5 mL (DCM), 1 mL (EtCN)	1	0.15	0.21	0.04	0	0	0.05
**6t (3)**	-40	15	5 mL (DCM), 1.5 mL (EtCN)	1	0.08	0.06	0.03	0	0	0.07
**6u (2)**	-40	15	5 mL (DCM), 0.5 mL (EtCN)	1	0.05	0.02	0	0.18	(overlap)	0.05
**6u (2)**	-30	15	4 mL (DCM), 1 mL (EtCN)	1	0.06	0.02	0	0	0	0
**6u (2)**	-30	15	9 mL (DCM), 3 mL (EtCN)	1	0	0	0	0	0	0
**6u (2)**	20	5	2 mL (DCM), 1 mL (MeCN)	1	0.07	0.03	0.02	0	0	0

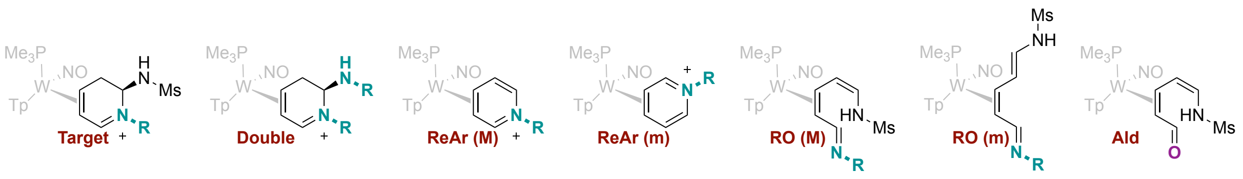

COMPUTATIONAL DETAILS

The conjectured dicationic intermediate **IV** was computationally investigated. The M06 functional and 6-31G(d, p) basis set (with the LANL2DZ effective core potential and basis set on tungsten) were used to model the *N*-methyl congener of the cation, with implicit acetonitrile solvation applied using the SMD model. Thermochemical corrections were applied at 298 K and 1 atm. Two distinct minima (verified to have no imaginary frequencies) were identified (Figure S69): a 'distal' (***d***) conformer wherein the tungsten center is best described as *trihapto*-coordinated to an allyl fragment, and a slightly lower-energy 'proximal' (***p***) conformer (ΔG -1.1 kcal/mol) wherein the tungsten is dihapto-coordinated to a proximal allylic fragment. The barrier between the two conformations was small ($\Delta G^{\ddagger}$ +2.4 kcal/mol), consistent with analogous 'allyl shift' transition states.[3] The transition state was verified to have one imaginary vibration only and was further verified by IRC to connect the correct minima.

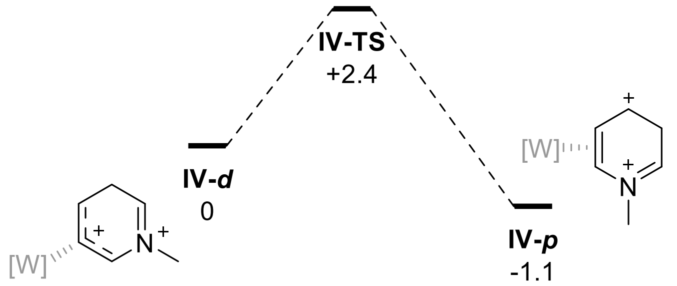

Figure S69. The two conformers of the proposed dicationic intermediate **IV**.

References:

(1) Dabbs, J. D.; Ericson, M. N.; Wilde, J. H.; Lombardo, R. F.; Ashcraft, E. C.; Dickie, D. A.; Harman, W. D. The Tungsten-Promoted Synthesis of Piperidyl-Modified erythro-Methylphenidate Derivatives. *ACS Central Science* **2023**. DOI: 10.1021/acscentsci.3c00556.
(2) Dabbs, J. D.; Ericson, M. N.; Dickie, D. A.; Harman, W. D. Synthesis of 1-Azatriene Complexes of Tungsten: Metal-Promoted Ring-Opening of Dihydropyridine. *Organometallics* **2024**, *43* (9), 1051-1056. DOI: 10.1021/acs.organomet.4c00108 From NLM PubMed-not-MEDLINE.
(3) Harrison, D. P.; Nichols-Nielander, A. C.; Zottig, V. E.; Strausberg, L.; Salomon, R. J.; Trindle, C. O.; Sabat, M.; Gunnoe, T. B.; Iovan, D. A.; Myers, W. H.; Harman, W. D., Hyperdistorted Tungsten Allyl Complexes and Their Stereoselective Deprotonation to Form Dihapto-Coordinated Dienes. *Organometallics* **2011,** *30* (9), 2587-2597.
